# Coumarin‐Tagged Dinuclear Trithiolato‐Bridged Ruthenium(II)⋅Arene Complexes: Photophysical Properties and Antiparasitic Activity

**DOI:** 10.1002/cbic.202000174

**Published:** 2020-06-16

**Authors:** Oksana Desiatkina, Emilia Păunescu, Martin Mösching, Nicoleta Anghel, Ghalia Boubaker, Yosra Amdouni, Andrew Hemphill, Julien Furrer

**Affiliations:** ^1^ Department of Chemistry and Biochemistry University of Bern Freiestrasse 3 3012 Bern Switzerland; ^2^ Institute of Parasitology Vetsuisse Faculty University of Bern Länggass-Strasse 122 3012 Bern Switzerland

**Keywords:** antiprotozoal agents, coumarin, cytotoxicity, ruthenium, sandwich complexes, *Toxoplasma gondii*

## Abstract

The synthesis, characterization, photophysical and biological properties of 13 new conjugate coumarin‐diruthenium(II)⋅arene complexes against *Toxoplasma gondii* are presented. For all conjugate organometallic unit/coumarins, an almost complete loss of fluorescence efficacy was observed. However, the nature of the fluorophore, the type of bonding, the presence and length of a linker between the coumarin dye and the ruthenium(II) moiety, and the number of dye units influenced their biological properties. The *in vitro* activity against a transgenic *T. gondii* strain grown in human foreskin fibroblasts (HFF) leads to IC_50_ values for *T. gondii* β‐gal from 105 to 735 nM. Of note is that nine compounds displayed lower IC_50_ than the standard drug pyrimethamine. One compound applied at its IC_50_ did not affect B‐cell proliferation but had an impact on T‐cell proliferation in murine splenocyte cultures. Transmission electron microscopy of *T. gondii* β‐gal‐infected HFF showed that treatment predominantly affected the parasites’ mitochondrion.

## Graphical abstract

**The (para)site for treatment**: Thirteen coumarin‐diruthenium(II)⋅arene conjugates were evaluated against a transgenic *T. gondii* strain grown in human foreskin fibroblasts (HFF). The IC_50_ values ranged from 105 to 735 nM, with nine compounds displaying a lower IC_50_ than the standard drug pyrimethamine. TEM of *T. gondii*‐infected HFF showed that treatment predominantly affected the parasites’ mitochondrion.

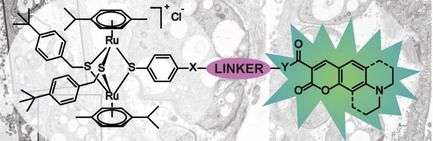

## Introduction

**Figure 1:**
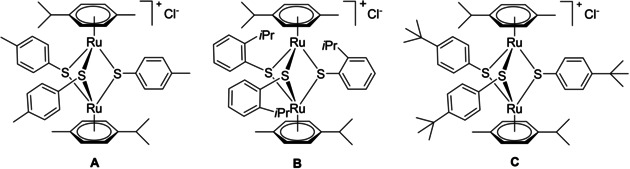
Structure of cationic trithiolato diruthenium(II)⋅arene complexes presenting high cytotoxicity against cancer cells and efficacy against various parasites.

The use of ruthenium complexes as potential chemotherapeutics has been an active area of research for almost two decades.^1^, ^2^, ^3^ Developed initially as a potential alternative to platinum‐based anticancer drugs,^4^, ^5^ ruthenium(II)⋅arene complexes were also considered for other pharmacological properties, particularly as antiparasitic^6^, ^7^, ^8^, ^9^, ^10^, ^11^, ^12^, ^13^ and antibacterial compounds.^14^, ^15^, ^16^, ^17^ A special class of ruthenium(II)⋅arene complexes is constituted by symmetric^18^, ^19^ and “mixed”^20^ cationic trithiolato‐bridged dinuclear ruthenium(II)⋅arene complexes (general formulae [(*η* ^6^‐arene)_2_Ru_2_(*μ*_2_‐SR)_3_]^+^ and [(*η* ^6^‐arene)_2_Ru_2_(*μ*_2_‐SR^1^)_2_(*μ*_2_‐SR^2^)]^+^, respectively). The high cytotoxicity against human cancer cells shown by this type of compounds and, more interestingly, their apparent ability to circumvent platinum‐drug resistance,^21^ encouraged the development of several libraries of complexes containing this scaffold as potential biological active compounds. For example, IC_50_ values as low as 30 nM against A2780 (human ovarian cancer) cells and also against their cisplatin‐resistant variant A2780cisR were measured for compounds **B** and **C** (Figure 1), whereas the less lipophilic complex **A** exhibited lower cytotoxicity (IC_50_ of 130 and 80 nM on A2780 and A2780cisR, respectively).

The mechanisms by which these trithiolato dinuclear ruthenium complexes exert their cytotoxicity are still not clearly determined. Due to their lipophilic nature, these trithiolato dinuclear ruthenium complexes are only sparingly soluble in water. In contrast, the diruthenium trithiolato moiety is stable, neither hydrolyzing nor interacting with biomolecules such as amino acids (other than cysteine), nucleotides, or glucose.^21^ This inertness towards hydrolysis and ligand substitution can be attributed to the lack of the typical very labile Ru^II^Cl bonds or carboxylate ligands that are present in many other Ru^II^⋅arene complexes. Nevertheless, weak binding to various proteins (human serum albumin, (HsA), transferrin (Tf), cytochrome *c* (Cyt *c*), ubiquitin (Ub), and myoglobin (Mb)) was demonstrated, albeit through hydrogen bonds and π–π interactions and not through covalent bonds.^21^ Interestingly, catalytic oxidation of bio‐relevant reducing agents including cysteine and glutathione (GSH) to form cystine and GSSG, respectively, was demonstrated.^22^ Although no correlation between the *in vitro* cytotoxicity and the catalytic activity on the oxidation reaction of glutathione could be observed,^19^, ^22^ it is nevertheless considered that this process can be at least partially responsible for the decreased cancer cell survival.

Recently, inductively coupled plasma mass spectrometry (ICP‐MS) showed that complexes **A** and **C** specifically target the mitochondrion in A2780 (human ovarian cancer) cells, with up to 97 % of the Ru content being found in the mitochondrial fraction.^23^ Complex **C** was taken up by A2780 cells more efficiently than **A**, which parallels with the respective lipophilicity of the two compounds.

Lately, trithiolato diruthenium compounds were reported to exhibit interesting antiparasitic properties against *Toxoplasma gondii*,^24^ *Neospora caninum* 23 and *Trypanosoma brucei*.^25^ Thus, complex **A** presents an IC_50_ value of 34 nM against the apicomplexan parasite *T. gondii* cultured in human foreskin fibroblast (HFF) monolayers,^24^ while compound **B** exhibits an IC_50_ of 4 nM against *T. brucei* bloodstream forms.^25^ Viability of uninfected HFF cells treated with 2.5 μM of complex **A** was decreased to 63 %, however complex **B** did not affect the vitality when applied at the same concentration.^24^ In *Toxoplasma, Neospora* and in *Trypanosoma*, transmission electron microscopy (TEM) demonstrated that trithiolato diruthenium compounds affected the structural integrity of the mitochondrion after few hours of treatment, and led to a more pronounced destruction of tachyzoites at later timepoints.^24^ In *T. brucei*, these compounds altered the mitochondrial membrane potential, while other organelles and structural elements of the parasites remained largely unaffected.^25^

The intracellular fate of these trithiolato dinuclear ruthenium complexes, and how they exert anticancer and antiparasitic effects, are unknown, and thus investigating their cellular traffic and possible mechanisms of action has become a priority. *In vitro*/in vivo tracking of organometallic drugs using traceable compounds represents a promising approach.^26^, ^27^ Various conjugates obtained by anchoring an imaging probe to the biologically active metal‐organic moiety were shown to facilitate the intracellular tracing of the metal‐based drugs.^28^, ^29^, ^30^ However, the modification of a drug with a fluorophore tag can strongly influence its physicochemical properties, and thus activity and behavior. It was already shown that anchoring different fluorophores (coumarin, BODIPY, porphyrin) on the same therapeutic organometallic moiety influences cellular internalization and accumulation.^28^, ^29^, ^30^ Thus, the optimization of the conjugate structure (the organometallic drug onto which the fluorophore probe is already anchored/tethered) should be considered from the beginning.

Microscopy studies of BODIPY,^29^, ^31^ coumarin^32^ and naphthalimide^33^‐tagged ruthenium conjugates demonstrated that these conjugates could be applied for investigating the subcellular localization of half‐sandwich ruthenium(II)⋅arene complexes. Due to their good photophysical properties, chemical stability, and ease of synthetic modifications, coumarin derivatives represent an important class of fluorescent probes for biological imaging.^34^, ^35^ Additionally, compounds presenting a coumarin scaffold display broad pharmacological activities and their potential as anticancer agents has received a lot of interest.^36^, ^37^, ^38^, ^39^, ^40^

**Figure 2:**
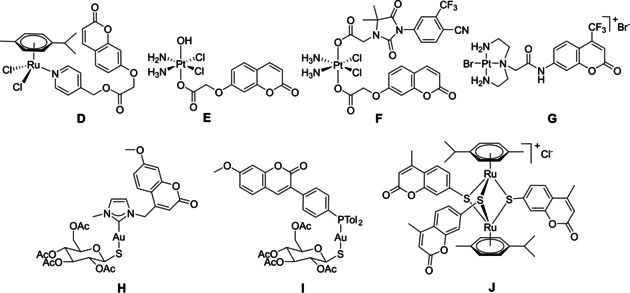
Structures of various organometallic moiety⋅coumarin conjugates.

In the particular case of traceable organometallic drugs, two strategies can be considered: 1) direct coordination of the (coumarin) fluorophore to the metal center^41^, ^42^ or 2) anchoring of the dye to more elaborated ligands.^[32,43, 44]^ The nature of the metal center as well as the type of the ligand can strongly affect the photophysical properties of the hybrid molecule. Some data on the development of trackable anticancer agents based on metal complexes were recently reviewed.^26^, ^45^ Conjugates combining metalorganic units with covalently linked coumarins have been shown to be versatile tools for imaging in the case of various fluorophore‐tagged platinum,^[43,44, 46]^ ruthenium,^32^ gold^[28,47, 46]^ or iridium^48^ complexes. Some examples of coumarin modified organometallic compounds are presented in Figure 2.

Coumarin‐tagged ruthenium(II)⋅arene complex **D** was shown to be useful for bioimaging.^32^ Laser scanning confocal microscope (LSCM) showed that compound **D** was internalized by HCT‐116 cells (human colorectal cancer, IC_50_=66 μM), causing intracellular fluorescence after 3 h of incubation when applied at 30 μM. The process occurred in a concentration‐dependent manner. Similar results were obtained with Pt^IV^ conjugate **E**,^44^ which was considered as potential tracking agent due to the presence of a coumarin moiety. Nevertheless, neither for conjugate **D** nor **E** were physical properties (in terms of fluorescence quantum yield *Φ*_F_) reported.

Compound **F** is a three‐in‐one hybrid containing both an androgen receptor (AR) binding ligand and a coumarin unit connected to a Pt^IV^ moiety.^46^ The fluorescence quantum yield of **F** was weaker than that of the respective coumarin ligand (*Φ*_F_=0.072 *vs* 0.32). **F** was effectively internalized and visualized by LSCM in LNCaP (AR+) cells (androgen‐sensitive human prostate adenocarcinoma cells overexpressing androgen receptor) after 4 h of incubation when applied at 20 μM. Derivative **G**, a Pt^II^ complex of a coumarin modified diethylenetriamine, was developed as a selective probe, fluorescent agent and inorganic medicinal agent.^43^ However, the fluorescence intensity of the coumarin moiety was reduced by 90 % in this conjugate, thus no microscopy studies on cellular uptake were reported. Several gold(I) complexes bearing coumarin units were described.^28^, ^47^, ^49^ For example gold(I) carbene complex **H** displayed poor photophysical properties (fluorescence quantum yield, *Φ*_F_=2 %), the fluorescence quenching being attributed to a photo‐induced electron transfer between the coumarin and the carbene.^47^ Nevertheless fluorescence confocal microscopy demonstrated the uptake of **H** in the nuclei of A2780 (human ovarian cancer) cells (IC_50_=12 μM) at a concentration of 50 μM. In contrast, gold(I) phosphine complex **I** showed interesting photophysical properties (*Φ*_F_=83 %), and two‐photon fluorescence microscopy of MDA‐MB‐231 (human breast adenocarcinoma) cells showed this compounds to accumulate at the plasma membrane as small aggregates.^28^ Interestingly, reports on coumarin trithiolato decorated compounds such as **J** 18, 50 focused only on the anticancer activity of the compounds and not on their photophysical properties.

The easy synthesis of symmetric and mixed trithiolato dinuclear ruthenium complexes presenting functional groups that further allow “chemistry on the complex” opens the possibility to prepare various conjugates with the molecules of interest, for example, fluorophores, drugs, or biomolecules (amino acids, fatty acids, polyamines, carbohydrates). Hybrid molecules with anticancer drug chlorambucil^51^ and short peptides^52^ were already reported as potential strategy to enhance the anticancer activity.

Herein, we report the design and synthesis of a library of new trithiolato‐bridged dinuclear ruthenium(II)⋅arene organometallic conjugates in which coumarin fluorophore moieties were anchored as pendant arms on the bridge thiol(s). A systematic assessment of the influence of various structural features of the conjugates (nature of the fluorophore, type of bonding (ester vs amide), presence and length of a linker between the coumarin dye and the binuclear ruthenium(II) moiety, number of dye units) upon the photophysical and biological properties was performed. All compounds of interest (coumarin‐labeled conjugates, unmodified thiolato‐bridged dinuclear ruthenium(II)⋅arene complexes and free dyes) were evaluated with respect to their activity against the transgenic strain *T. gondii* β‐gal grown in HFF host cells. In addition, the cytotoxicity in uninfected HFF was assessed. For selected compounds, the potential to impair immunity was assessed using B‐ and T‐cell proliferation assays, and TEM was carried out to evaluate structural alterations in treated parasites.

## Results and Discussion

### Synthesis and characterization of the compounds

In this study, the influence of various structural features of the coumarin conjugates (nature of the fluorophore, type of bonding (ester vs amide), presence and length of a linker/spacer between the dye and the diruthenium moiety, number of the fluorophore units) upon the photophysical and biological properties was evaluated.

Coumarin derivatives present a wide structural diversity due to the diﬀerent substitution possibilities within their basic motif that contains a benzene ring fused to an α‐pyrone. Analogues substituted with electron‐donating groups in position 7 such as 7‐methoxy and 7‐diethylamino coumarins are frequently used fluorophores. The two coumarin dyes considered for this study, namely 7‐(diethylamino)‐2‐oxo‐2*H*‐chromene‐3‐carboxylic acid (**Dye1**‐CO_2_H) and 11‐oxo‐2,3,6,7‐tetrahydro‐1*H*,5*H*,11*H*‐pyrano[2,3‐*f*]pyrido[3,2,1‐*ij*]quinoline‐10‐carboxylic acid (also known as butterfly coumarin 343, **Dye2**‐CO_2_H) present easy derivatizable carboxylic group. The use of these particular fluorophores is justified by their good quantum yields, appropriate absorption/emission wavelengths (different emission colors), lipophilicity, lack of net ionic charge, photostability, and relatively small size.^34^, ^35^ It is well known that the photophysical properties of the coumarin derivatives can be tuned with small changes in the substituents and their position.^53^ If these two coumarins present very similar structure, previous reports associated the blockage of the free rotation of the C−N bond in **Dye2**‐CO_2_H, which constrains the nitrogen lone pair to a maximal interaction with the aromatic rings, with an improvement of the photophysical properties compared to **Dye1**‐CO_2_H.^54^, ^55^ Nevertheless, if this interaction results in higher quantum yield in aqueous solution for **Dye2**‐CO_2_H than the diethylamino‐coumarin **Dye1**‐CO_2_H, the coumarin substituent is more bulky and hydrophobic.

A SAR study^56^ performed on a library of trithiolato‐bridged dinuclear ruthenium(II)⋅arene compounds showed that the nature of the substituents in the *para* position of the other two thiol ligands influence the biological properties of the complexes. Some of the compounds were developed also in an analogue series in which the bulky hydrophobic *t*Bu substituents were replaced by hydrophobic polar CF_3_ groups, in order to determine the role of this type of structural modifications upon the biological activity of the conjugates.

**Scheme 1:**
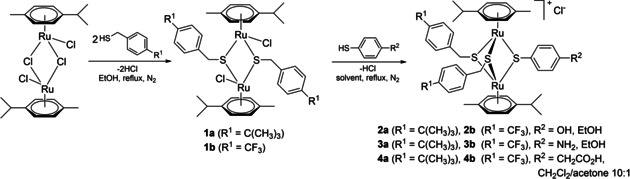
Synthesis of the dinuclear dithiolato **1 a**/**b** and OH‐, NH_2_‐, CH_2_CO_2_H‐functionalized trithiolato ruthenium(II)⋅arene intermediates **2 a**/**b**, **3 a**/**b** and **4 a**/**b**.

The first series of compounds were functionalized with one coumarin unit anchored to one of the bridged thiols, providing a ratio metal‐organic moiety: dye of 1 : 1. To access this type of analogues, the previously reported^56^ dinuclear trithiolato ruthenium(II)⋅arene intermediates, bearing one hydroxy **2 a**/**b**, amino **3 a**/**b** and carboxylic acid group **4 a**/**b**, were prepared starting from the ruthenium dimer ([Ru(**η** ^6^‐*p*‐MeC_6_H_4_ *i*Pr)Cl]_2_Cl_2_) in two synthetic steps (Scheme 1).^20^ Obtaining this type of mixed trithiolato complexes is facilitated by the easy synthesis of the dinuclear dithiolato intermediates **1 a**/**b** which were isolated in high yields. In the case of the carboxylate functionalized trithiolato complexes **4 a**/**b** a mixture of CH_2_Cl_2_/acetone (10 : 1, *v*/*v*) was used as solvent to avoid esterification side reaction catalyzed by the hydrochloric acid resulted during the reaction).

**Scheme 2:**
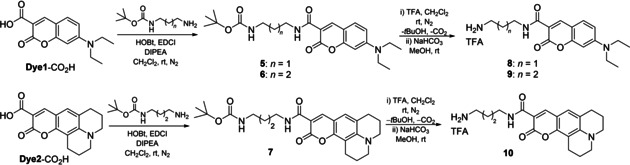
Synthesis of the coumarin precursors containing an amino spacer **8**, **9** and **10**.

**Scheme 3:**
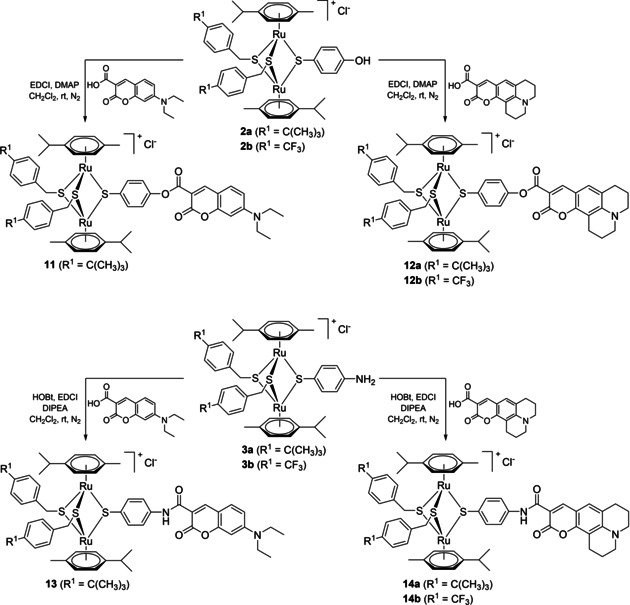
Synthesis of the coumarin‐based ester **11**, **12 a**/**b** (top) and amide **13**, **14 a**/**b** (bottom) conjugates.

If intermediates **2 a**/**b** and **3 a**/**b** could be directly coupled with the two dyes considered **Dye1**‐CO_2_H and **Dye2**‐CO_2_H through ester or amide bonds, respectively (Scheme 3), the carboxylate functionalized trithiolato complexes **4 a**/**b** offer the possibility to introduce linkers of variable length between the organometallic moiety and the dye. To this end, the linker modified coumarins **8**, **9** and **10** were synthesized in two steps staring from the corresponding coumarins (**Dye1**‐CO_2_H or **Dye1**‐CO_2_H) and the appropriate monoprotected diamine (Scheme 2).

**Scheme 4:**
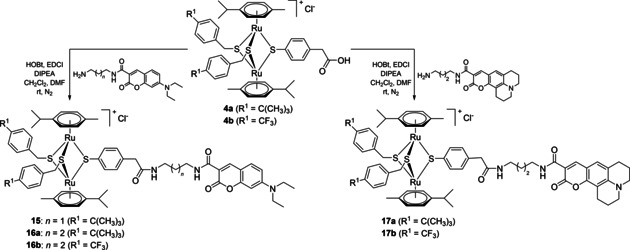
Synthesis of the coumarin conjugates **15**, **16 a**/**b** and **17 a**/**b** containing an amino spacer.

The coupling reactions of **2 a**/**b** and **3 a**/**b** with fluorophores **Dye1**‐CO_2_H and **Dye2**‐CO_2_H afforded the ester **11**, **12 a**/**b** and amide **13**, **14 a**/**b** conjugates in medium to good yields (65–99 %; Scheme 3). The coumarin hybrids **15**, **16 a**/**b** and **17 a**/**b** containing an amino spacer were obtained in reasonable yields (12–69 %) by reacting the carboxylate functionalized trithiolato complexes **4 a**/**b** with the judiciously functionalized fluorophores **8**, **9** and **10** (Scheme 4). The esterification reactions were realized using 1‐ethyl‐3‐(3‐dimethylaminopropyl)carbodiimide (EDCI) as coupling agent and 4‐dimethylaminopyridine (DMAP) as catalyst. For the amide bond formation, the coupling reactions were conducted in the presence of 1‐hydroxybenzotriazole (HOBt) and EDCI as coupling agents and *N,N‐*diisopropylethylamine (DIPEA) as basic catalyst. As previously reported in other conjugation reactions run on the trithiolato diruthenium complexes, the organometallic scaffold is stable to ligand exchange in the reaction conditions used.^51^, ^52^

**Scheme 5:**
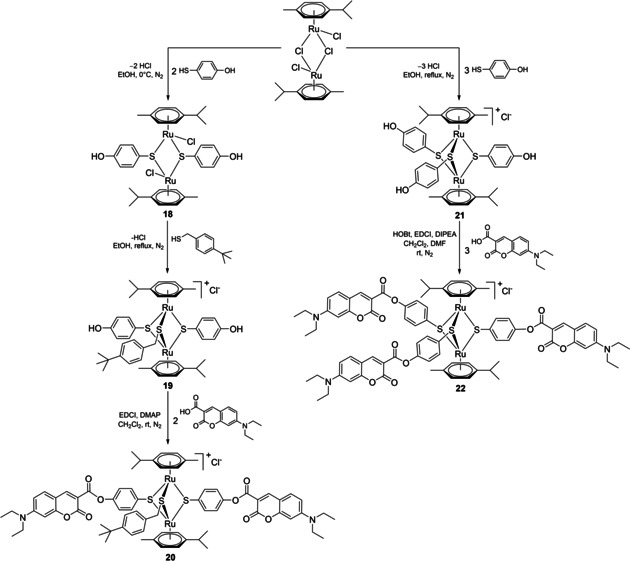
Synthesis of conjugates **20** and **22** bearing two and three fluorophore units, respectively.

A second family of conjugates with organometallic unit: fluorophore (dye) ratios of 1 : 2 and 1 : 3 was also synthesized (Scheme 5) and their photophysical and biological properties were evaluated.

Conjugate **20** functionalized with two coumarin units was synthesized in three steps starting from ([Ru(**η** ^6^‐*p*‐MeC_6_H_4_Pr^*i*^)Cl]_2_Cl_2_). If the synthesis of the dithiolato intermediates **1 a**/**b**, obtained by the complexation of less active benzylic thiols, could be easily controlled by the judicious use of the reagents ratio, the use of more reactive aromatic thiol of 4‐mercaptophenol led to complex mixture of the desired dithiolato product **18**, accompanied by trithiolato and monothiolato complexes, as well as unreacted ruthenium dimer. Nevertheless, the next two steps: 1) complexation of a third thiol with the obtainment of mixed trithiolato compound **19** bearing two hydroxy groups, and 2) subsequent esterification reaction with **Dye1**‐CO_2_H leading to dicoumarin conjugate **20** proceed smoothly, with isolated yields of 96 and 75 %, respectively (Scheme 5).

The trisubstituted conjugate **22** was obtained in two steps, the synthesis of the symmetric trihydroxy intermediate **21** (previously reported^57^) followed by the anchoring of three coumarin dye units, and was isolated in good yield (61 %).

All compounds were characterized by ^1^H, ^19^F (when appropriate), and ^13^C NMR spectroscopy, mass spectrometry, and elemental analysis (see the Supporting Information for full details).

For example, the ^1^H NMR spectra of hydroxy **2 a** and amine **3 a** intermediates differ from those of the respective ester and amide conjugates **11**/**12 a** and **13**/**14 a**. After anchoring the coumarin unit via an ester bond, for **11** and **12 a** the two protons in the α position to the ligand C−O are observed at slightly lower frequencies, by about Δ*δ*_H_ ≈0.05 ppm, whereas the protons in the β position with respect to the C−O bond are shifted towards higher frequencies Δ*δ*_H_ ≈0.32 ppm. In the ^13^C NMR spectra of esters **11** and **12 a** the corresponding C atoms α to the ligand C−O are observed at higher frequencies than in hydroxy intermediate **2 a**, Δ*δ*_C_ ≈5.6 ppm. Following esterification, a strong effect is observed on the ligand C−O, which is shifted to lower frequencies with Δ*δ*_C_ ≈8.6 ppm compared to C−OH **2 a**. In the case of amides **13** and **14 a**, the signal corresponding to the two protons in the *α* position to the ligand C−NH are strongly shifted to higher frequencies, by about Δ*δ*_H_ ≈0.96 ppm, whereas a similar but less important effect of only Δ*δ*_H_ ≈0.25 ppm is observed in the case of the corresponding protons in the β position with respect to the C−NH. The ^13^C NMR spectra of amides **13** and **14 a** shows the C atoms α to the ligand C−NH at higher frequencies than in amine **3 a** with Δ*δ*_C_ ≈4.8 ppm. After amide bond formation, the signal corresponding to the ligand C−NH is strongly shifted to lower frequencies with Δ*δ*_C_ ≈9.6 ppm compared to the respective signal/peak C−NH_2_ in compound **3 a**. Less important shifts are observed in the ^1^H NMR spectra of amides **15**, **16 a** and **17 a** compared to acid intermediate **3 a** for the corresponding protons α to the ligand C−CH_2_CONH due to the presence of the methylene spacer.

Interesting, in the ^1^H NMR spectra of the di‐hydroxy intermediate **19** and of the diester **20**, two sets of signals/peaks corresponding to the two 4‐mercaptophenolic ligands are observed, indicating that they are not equivalent in the NMR time scale measurement. Electrospray ionization mass spectrometry (ESI‐MS) corroborated the spectroscopic data. The spectra of the dithiolato intermediates contain a parent ion peak attributable to the [*M*−Cl]^+^ ion, formed after the loss of one of the labile chlorine ligands. The ESI‐MS of the cationic trithiolato complexes exhibit a strong peak corresponding to [*M*−Cl]^+^ ion.

The compounds containing the trithiolato scaffold (intermediates as well as conjugates) are stable towards ligand exchange in highly complexing solvent as [D_6_]DMSO even after elongated storage at 0 °C (see Figures S2–S4 in the Supporting Information for the spectra corresponding to compounds **2 a**–**4 a** and **11**–**17 a**). This is essential to validate the biological tests for which compounds are used as stock solutions in DMSO.

**Figure 3:**
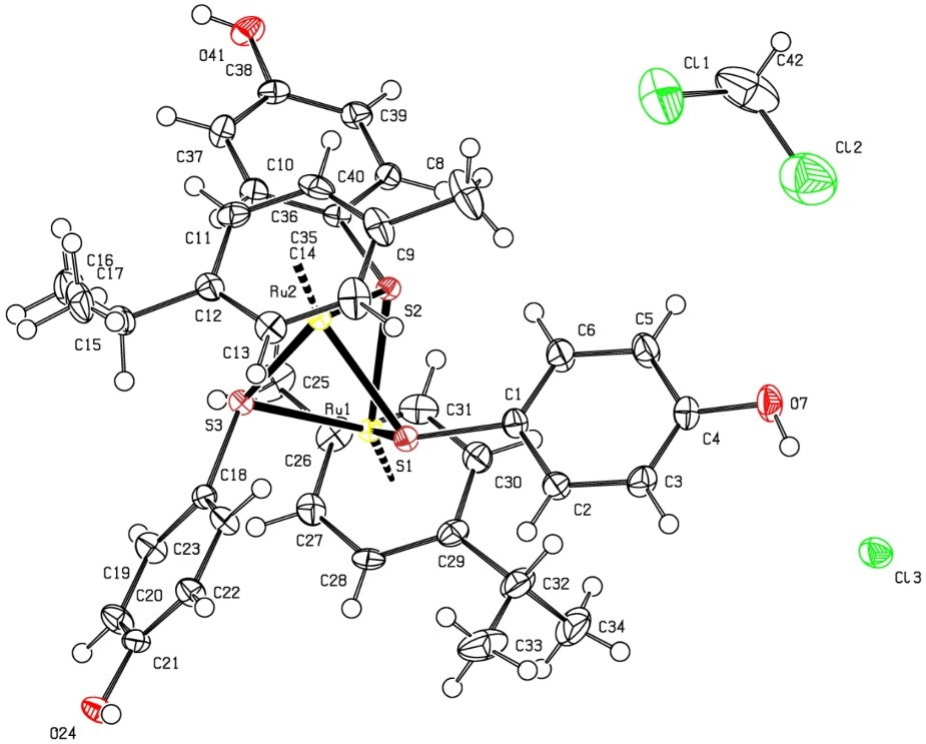
ORTEP representation of complex **21** (thermal ellipsoids are 50 % equiprobability envelopes, and H atoms are spheres of arbitrary diameter; the asymmetric unit contains also one CH_2_Cl_2_ molecule).

The solid‐state structure of symmetric intermediate **21** containing three free hydroxy groups was established by single crystal X‐ray diffraction analysis (Figure 3), confirming the expected molecular structure.

### X‐ray crystallography

The crystal structure of the symmetric complex **21** was established in the solid state by single‐crystal X‐ray diffraction (ORTEP representations shown in Figure 3), confirming the expected structure. Selected bond lengths and angles are presented in Table S2. Data collection and refinement parameters are given in Table S1.

The Ru_2_S_3_ unit forms a trigonal‐bipyramidal framework; no metal–metal bond are present, the corresponding Ru(1)C−Ru(2) distance being 3.341 Å. In the cation, the values of RuC−S bonds as well as the RuC−SC−Ru angles (Table S2) are similar to those found in other symmetric *p*‐cymene derivatives reported previously.^18^

In the network of **21** a complex interplay of H‐bonding interactions involving the chlorine anions and the three OH groups of the complex in observed (Table S3). Each Cl^−^ ion interacts with the OH groups from three different cationic complexes. A dimeric organization is saw, which is mediated by four H‐bonding interactions: two chlorine anions bridge two diruthenium units at the level of their respective two hydroxy groups (Figure S1). The remaining hydroxy group of each of the symmetric diruthenium complexes is involved in H‐bonding interactions with another Cl^−^ ion. Thus, all three hydroxy groups of the symmetric trithiolato di‐nuclear ruthenium(II)⋅arene complex **21** are involved in intermolecular H‐bonding interactions, two of them interact with two chlorine anions and lead to the formation of a dimer with another diruthenium unit, whereas the third OH group interacts through H‐bonds with other Cl^−^ anion present. In network these intermolecular H‐bonding interactions lead to further arrangements, for example, with the formation of cycles involving up to four cationic complexes.

### Photophysical characterization

**Figure 4:**
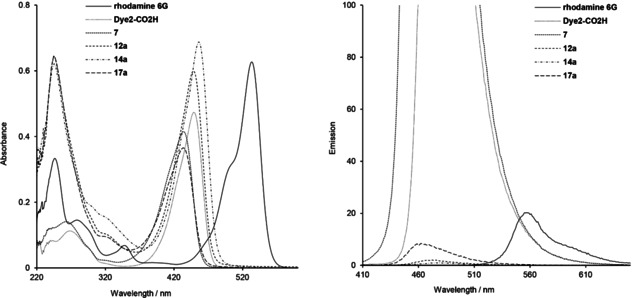
UV/Vis absorption (left) and emission (right) spectra of rhodamine 6G, **Dye2**‐CO_2_H, intermediate **7**, and the corresponding ester **12 a** and amide **14 a**, **17 a** conjugates at 10 μM in CHCl_3_.

**Figure 5:**
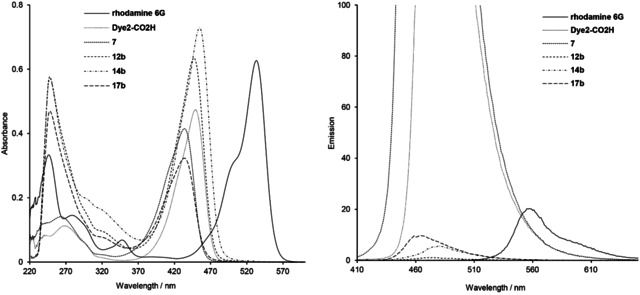
UV/Vis absorption (left) and emission (right) spectra of rhodamine 6G, **Dye2**‐CO_2_H, intermediate **7** and the corresponding ester **12 b** and amide **14 b**, **17 b** conjugates, at 10 μM in CHCl_3_.

The photophysical properties of coumarin containing compounds investigated in this study, namely the starting **Dye1**‐CO_2_H and **Dye2**‐CO_2_H, coumarin‐based amino intermediates **5**–**7**, and ester and amide conjugates with the trithiolato ruthenium(II)‐*p*‐cymene scaffold **11**–**17 a**/**b**, **20** and **22**, were studied in CHCl_3_ and EtOH at room temperature and are summarized in Tables 1 and S4. No solvatochromism was observed. The absorption and emission spectra of representative compounds for 10 μM solutions in CHCl_3_ and/or EtOH are comparatively presented in Figures 4, 5 and S5–S7.

**Table 1:** Photophysical data of compounds **5**–**17 a**/**b**, **20** and **22** in CHCl_3_ at room temperature.

Compound	λabsmax [nm]	*ϵ* [M^−1^ cm^−1^]	λemmax [nm]	Δ*λ* [nm]	*Φ*_F_ [%]
rhodamine 6G^[a]^	533	62 696.6	557	24	75^[a]^
**Dye1**‐CO_2_H	431	43 074.2	458	27	184
**Dye2**‐CO_2_H	449	47 372.9	474	25	158
**5**	418.5	42 971.9	449	30.5	186
**6**	417.5	47 042.6	448	30.5	175
**7**	433.5	41 504.7	463	29.5	170
**11**	431, 245	57 147.6, 64 027.8	455	24	4
**12 a**	448, 245.5	59 950.3, 64 535.9	472	24	1
**12 b**	447, 247.5	57 647.7, 63 481.4	474	27	0.4
**13**	439.5, 246	73 039.4, 68 176.6	458	18.5	0.3
**14 a**	456, 245.5	68 769.4, 62 124.7	478	22	2
**14 b**	454.5, 248	57 340.8, 72 892.3	480	25.5	2
**15**	418.5, 245.5	37 245.8, 63 190.6	454	35.5	3
**16 a**	417, 245.5	39 772.3, 65 900.6	449	32	3
**16 b**	417, 248	50 370.3, 34 021.4	449	32	2
**17 a**	433.5, 245.5	36 532.7, 64 378.6	462	28.5	3
**17 b**	433.5, 248	46 995.0, 32 315.8	465	31.5	3
**20**	431.5, 248.5	105 690.0, 57 402.0	457	25.5	0.3
**22**	432, 246	136 190.0, 55 390.8	459	27	0.4

[a] Values taken from ref. [58].

The absorption spectra of all diruthenium unit/coumarin conjugates **11**–**17 a**/**b**, **20** and **22** present a similar profile (Figures 4, 5 and S5–S7). In both solvents, strong peaks corresponding to the coumarin fragment are observed in the 410–460 nm region. The signals in the 200–300 nm range, associated with the trithiolato dinuclear ruthenium(II)⋅arene moiety, are better resolved in the spectra measured in CHCl_3_ compared to those in EtOH. For **11** and **12 a**/**b**, the direct attachment of the trithiolato diruthenium moiety to coumarins by ester bonds induced no shifts of the absorption peaks, whereas in case of amides **13** and **14 a**/**b** a slight bathochromic shift (Δλ ≈10 nm) was observed. In contrast, the presence of the di‐amino linker in conjugates **15**–**17 a**/**b** led to a slender hypsochromic shift (Δ*λ* ≈12–16 nm).

When excited at 405 nm, all coumarin‐containing compounds **5**–**17 a**/**b**, **20** and **22** emit in the blue range (450–490 nm). The emission spectra of conjugates **11**–**17 a**/**b**, presenting a 1 : 1 ratio organometallic unit: coumarin, show similar profiles (Figures 4, 5 and S5–S7) with almost complete fluorescence quenching, only slightly less pronounced for solutions in CHCl_3_ compared to those in EtOH. This loss of fluorescence efficacy was independent of the nature of coumarin (**Dye1**‐CO_2_H or **Dye2**‐CO_2_H), the type of bond (ester or amide) or presence of a di‐amino linker between the two moieties. From this compound library, the highest calculated fluorescence quantum yields remain very modest (*Φ*_F_ ≈3 %). Of note, similar but less pronounced quenching effects were observed in other organometallic coumarin‐based conjugates.^[26,32, 43]^

For all coumarin conjugates (**11**–**17 a**/**b**, **20** and **22**) a small bathochromic shift of the fluorescence maximum is observed in EtOH compared to CHCl_3_ (Δ*λ* ≈10 nm). The dramatic fluorescence intensity change depending on the presence, or not, of the trithiolato diruthenium unit could be valorized to monitor the stability of the conjugates towards hydrolysis and their behavior *in vitro*. Further structural optimization (as for example the introduction of longer or more rigid spacers between the diruthenium and fluorophore moieties) is required in order for this type of conjugates to be used also as trackable theranostics for the trithiolato di‐nuclear ruthenium(II)⋅arene fragment.

In the case of conjugates **20** and **22** with organometallic moiety/dye ratios of 1 : 2 and 1 : 3, respectively, a proportional increase was observed for the coumarin absorbance signal (maximum at *λ*=431–432 nm), which parallels the number of the attached dye units. No significant effect was observed for the emission signal at 457–459 nm, corresponding to the trithiolato diruthenium(II)⋅arene unit/moiety.

Coumarin intermediates **5**–**10** and conjugates bearing di‐amino linkers **15**–**17 a**/**b** present slightly larger Stokes shifts compared to free **Dye1**‐CO_2_H and **Dye2**‐CO_2_H and other hybrid molecule**s**. Calculated Stokes shifts values range between 22–36 nm for experiments performed using solutions in CHCl_3_, and in 31–49 nm interval when EtOH was used as solvent.

**Figure 6:**
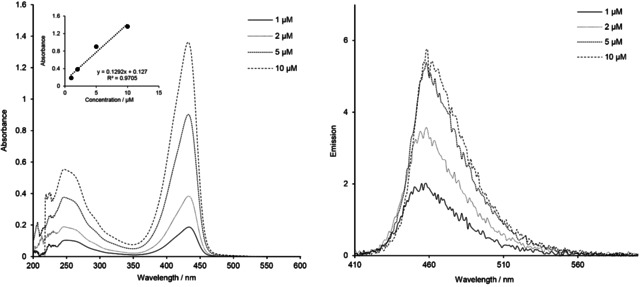
UV/Vis absorption (left) and emission (right) spectra of tricoumarin ester conjugate **22** at various concentrations in CHCl_3_.

In both solvents, CHCl_3_ and EtOH, a linear dependence of the absorbance and emission intensity with concentration was determined (data not shown). This is observed even in the case of the conjugate **22** bearing three **Dye1**‐CO_2_H units (spectra of **22** at various concentrations in CHCl_3_ are summarized in Figure 6 and Figure S7).

### Photostability

As the fluorescence intensity dramatically drops upon the addition of the trithiolato diruthenium unit (coumarin–ruthenium conjugates **11**–**17 a**/**b**, **20** and **22** compared to free **Dye1**‐CO_2_H and **Dye2**‐CO_2_H), we used this difference to monitor the stability of the hybrid molecules. To verify the compounds’ bleaching sensitivity but also stability at room temperature under air, in the presence of a polar solvent that can induce solvolysis, 10 μM **s**olutions of **12 b**, **14 b**, **16 b**, **17 b**, **20** and **22** in EtOH were maintained under indoor light for an extended time period. Absorption and emission spectra were monitored after 24 h, 48 h, and one‐week of light exposure, the measurements being made in the same conditions as previously described. Absorption spectra of all compounds remained unchanged after 48 h. After one week of light exposure a minor decrease of intensity can be observed for the absorbance signal in the 350–480 nm range, corresponding to the coumarin fragment.

**Figure 7:**
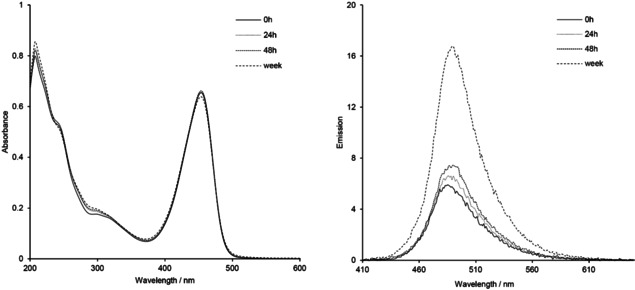
UV/Vis absorption (left) and emission (right) spectra of the trithiolato ruthenium(II)⋅*p*‐cymene complex **14 b** after 0 h, 24 h, 48 h, and one week (168 h) of exposure to indoor light at 10 μM in EtOH.

In the emission spectra, notable changes were observed only for ester conjugates **12 b**, **20**, and **22** (data not shown). A similar effect was noticed for the amide conjugates **14 b** and **17 b** (Figure 7), but only after one week of light exposure, while no spectral changes were observed for amide **16 b**. This increase of the emission signal can be attributed to a partial solvolysis of the ester or amide bonds present on the conjugates with the release of the respective coumarin dyes. Nevertheless, considering the time scale, and seen the very high fluorescence efficacy of **Dye1**‐CO_2_H, **Dye2**‐CO_2_H and corresponding coumarin intermediates **5**–**10** compared to the emission of the conjugates (fluorescence almost entirely quenched in the hybrid molecule), we can conclude that the coumarin‐organometallic conjugates present high stability in the conditions used for this experiment.

### Biological activity of the coumarin conjugates against *Toxoplasma gondii*

Toxoplasmosis, one of the most common global zoonotic diseases, is caused by the protozoan parasite *T. gondii*.^59^ This parasite can infect virtually all warm‐blooded animals on the planet and has very high zoonotic potential. Up to one third of the human population is infected with *T. gondii*, but only a small fraction of infected individuals exhibits clinical signs. The economic impact of *T. gondii* is enormous, in that it causes severe losses in a wide range of wild and domestic animals, including most animals used in food production. In general, *T. gondii* infestation remains without clinical symptoms in immune competent individuals, and no treatment is required. However, in humans, infection has been linked to neuropsychiatric disease, and upon immunosuppression, or primary infection during pregnancy, *T. gondii* can cause toxoplasmosis, a life‐threatening disease affecting both humans and animals that can lead to severe pathology including fetal malformation and abortion.^60^ Current standard treatment options for toxoplasmosis include macrolide antibiotics and sulfonamides,^61^ which inhibit protein biosynthesis and intermediary metabolism in the apicoplast, a prokaryote‐like organelle that is unique to apicomplexans.^62^ However, these treatments are often characterized by adverse side effects and do not act in a parasiticidal manner. The development of novel treatment options that specifically target the parasite is therefore of prime importance.

**Figure 8:**
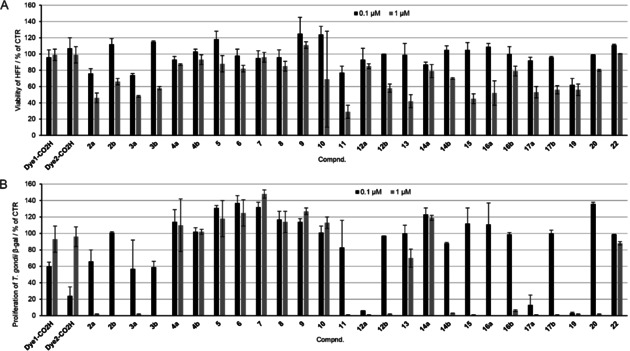
*In vitro* activities of all compounds at 0.1 and 1 μM on A) HFF viability and B) *T. gondii* β‐gal tachyzoites proliferation, in relation to treatments with 0.1 % DMSO. For each assay, standard deviations were calculated from triplicate experiments.

The compounds presented in this study were screened for biological activity *in vitro* against *T. gondii* β‐gal, a transgenic strain that constitutively expresses β‐galactosidase, which is grown in HFF monolayers. In addition, the effects on uninfected HFF host cells were assessed. For the primary screening, cell cultures were exposed during 3 days to 1 and 0.1 μM of each compound (including unmodified thiolato‐bridged dinuclear ruthenium(II)⋅arene complexes **2 a**/**b**, **3 a**/**b**, **4 a**/**b**, coumarin‐labeled conjugates **11**–**17 a**/**b**, **20** and **22**, free dyes **Dye1**‐CO_2_H and **Dye2**‐CO_2_H, and corresponding coumarin‐based intermediates). The viability of HFF cultures following drug treatments was measured by alamarBlue assay, and the proliferation of *T. gondii* was quantified by measuring β‐galactosidase activity. The results of this primary screening are presented as percentage in relation to untreated control cultures in Table S5. The results obtained at concentration of 0.1 and 1 μM of tested compound for *T. gondii* and HFF are presented in Figure 8, in relation to controls (CTR), namely HFF treated with 0.1 % DMSO exhibiting 100 % viability, and *T. gondii* β‐gal tachyzoites treated with 0.1 % DMSO showing 100 % proliferation.

No clear structure‐activity relationship could be identified. The observed activity/cytotoxicity appeared to be the result of an interplay of various structural parameters that influence the cellular internalization and further interactions with biomolecules. The trithiolato dinuclear derivatives are mono‐cationic complexes with a substantial molecular weight (>950 g/mol). Anchoring of hydrophobic coumarin analogues as pendant arms on one of the thiol ligands, and the nature of the substituents present on the other two thiols, influence the physicochemical properties of the molecule.

Compounds **2 a**/**2 b** and **3 a**/**3 b** bearing one hydroxy or amino group presented considerable activity against *T. gondii* β‐gal at 1 μM, but also impaired HFF cell viability. The same was observed in the case of dihydroxy compound **19**. Acid functionalized trithiolato complexes **4 a** and **4 b** did not affect the viability of HFF monolayers, but did also not affect tachyzoite proliferation at 1 μM. None of the coumarin‐based intermediates **5**–**10** (Table S5 and Figure S8) impacted on HFF viability or *T. gondii* β‐gal proliferation.

The nature of the substituents on the other two thiol ligands, hydrophobic‐bulky *tert*‐butyl versus hydrophobic‐polar trifluoromethyl, had a considerable influence in the case of the hydroxyl derivatives **2 a**/**2 b** compared to the amino analogues **3 a**/**3 b**. This structural feature (*t*Bu vs CF_3_) appeared to affect the activities of the ester compounds **12 a**/**12 b** and the amide compounds **14 a**/**14 b** in which the coumarin is directly connected to the thiol ligand. In some cases, (**11** vs **13**, **12 a** vs **14 a**,), the amide conjugates exhibited lower antiparasitic activity compared to the esters for the same coumarin substituent.

**Dye1**‐CO_2_H ‐functionalized compounds **13**, **15**, and **16 a** presented a similar cytotoxicity profile for HFF at 0.1 and 1 μM. The introduction of the linker augmented the measured activity against the parasite. For compounds bearing **Dye2**‐CO_2_H moieties, the introduction of a linker between the diruthenium scaffold and the coumarin led to a substantially increased antiparasitic activity in the case of *t*Bu analogue **17 a** compared to **14 a**; however, a similar effect was not observed for CF_3_ analogues **17 b** and **14 b**. Compounds **13**, **14 a** and **22** are only poorly active against *T. gondii* β‐gal at 1 μM, and the last two compounds did not notably affect HFF cell viability. In the series of **Dye1**‐CO_2_H functionalized ester compounds **11**, **20** and **22** no noteworthy correlation between the number of coumarin units and the measured biological activity was observed. At 1 μM, **11** displayed substantial HFF toxicity, while **20** and **22** did not. As shown in Figure 8, **Dye1**‐CO_2_H and **Dye2**‐CO_2_H did not affect viability of HFF when applied at 0.1 or 1 μM. However, proliferation of *T. gondii* relative to the rate of β‐galactosidase activity was decreased following treatment with 0.1 μM of **Dye1**‐CO_2_H (53 %) and **Dye2**‐CO_2_H (69 %; Table S5). These results might suggest that the *in vitro* anti‐toxoplasma activity of these coumarin dyes may be lost due to solubility issues.

Based on this preliminary screening, 13 compounds were selected for determination of the IC_50_ values against *T. gondii* β‐gal. These were compounds that, when applied at 1 μM, allowed viability values for HFF at 45 % or more, while they also inhibited *T. gondii* proliferation by 94 % or more. The results are shown in Table 2 and Figure S8, and the respective dose–response curves are shown in Figure S9.

**Table 2:** IC_50_ values [μM] against *T. gondii* β‐gal tachyzoite proliferation and effects on HFF viability at 2.5 μM, for selected compounds and pyrimethamine (as positive control).

Compound	IC_50_ [μM]	[LS; LI]^[a]^	SE^[b]^	HFF viability at 2.5 μM [%]^[c]^	SD^[d]^
2 a*	0.117	[0.098; 0.139]	0.0510	56	6
**2 b**	0.336	[0.323; 0.35]	0.0088	72	6
3 a*	0.153	[0.127; 0.185]	0.0488	51	5
**3 b**	0.135	[0.105; 0.174]	0.0562	53	4
12 a*	0.105	[0.099; 0.111]	0.0230	58	4
**12 b**	0.298	[0.292; 0.305]	0.0051	28	1
**14 b**	0.391	[0.351; 0.437]	0.0236	71	5
**16 a**	0.127	[0.170; 0.095]	0.129	4	1
**16 b**	0.735	[0.467; 1.156]	0.1155	73	7
**17 a**	0.243	[0.190; 0.311]	0.0905	25	5
**17 b**	0.203	[0.113; 0.366]	0.1459	54	7
**19**	0.115	[0.098; 0.135]	0.0447	2	4
**20**	0.377	[0.36; 0.39]	0.0137	71	4
pyrimethamine	0.326	[0.288; 0.396]	0.0518	99	6

[a] Values at 95 % confidence interval (CI); LS (limit superior) and LI (limit inferior) are the upper and lower limits of the CI, respectively. [b] The standard error (SE) of the estimate represents the average distance that the observed values fall from the regression line (*T. gondii* β‐gal). [c] Control HFF treated only with 0.25 % DMSO exhibited 100 % viability. [d] The standard deviation of the mean of six replicate experiments (HFF). Asterisks indicate the three compounds selected for assessment of their potential to interfere in splenocyte proliferation *in vitro*.

The most active compound against *T. gondii* β‐gal was **12 a**, with an IC_50_ value of 0.105 μM. This compound was not toxic at 1 μM, but when applied at 2.5 μM it reduced HFF viability to 58 %. Higher levels of HFF viability impairment were noted for compounds **12 b**, **17 a** and especially **19** (98 % reduction in HFF viability). The difference in antiparasitic activity between compounds **12 a** and **12 b** (IC_50_ values of 0.105 and 0.298 μM, respectively) highlights the importance of the physicochemical properties of the substituents present on the other thiol ligands, *t*Bu versus CF_3_. As shown in Table 2, unmodified hydroxy compound **2 a** and amino compounds **3 a** and **3 b**, also presented considerable antiparasitic activities (IC_50_ values of 0.117, 0.153, and 0.135 μM, respectively), furthermore, their impact on HFF viability at 2.5 μM was less pronounced compared to **17 a** and **19**.

**Figure 9:**
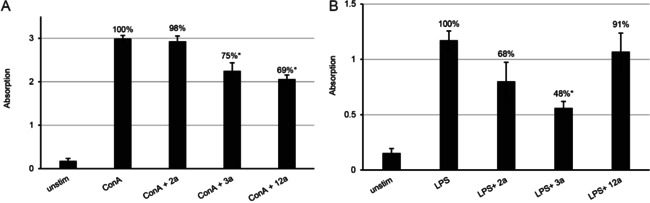
Inhibitory effect of selected compounds **2 a**, **3 a** and **12 a** on A) ConA‐ and B) LPS‐induced proliferative activity of mouse splenocytes. Bars represent standard deviation from the mean of four replicates. 100 % proliferation is attributed to the control (ConA or LPS); values are percentage proliferation compared to control, * *p* <0.01 in relation to controls.

Three selected compounds (**2 a**, **3 a** and **12 a**), applied at their respective IC_50_ against *T. gondii*, were further assessed with respect to their potential to interfere in splenocyte proliferation *in vitro*. Isolated murine splenocytes from healthy mice were stimulated with concanavalin A (ConA) to induce T‐cell proliferation or with bacterial lipopolysaccharide (LPS) to induce B‐cell proliferation, either in the presence or absence of tested compounds **2 a**, **3 a** and **12 a**. Measurements of proliferation were done using an assay that quantifies the incorporation of 5‐bromo‐2’‐deoxy‐uridine (BrdU) into the DNA of replicating cells. As seen in Figure 9, **3 a** and **12 a** significantly interfered with T‐cell proliferative responses, which resulted in a reduction of BrdU incorporation by 25 and 31 %, respectively, whereas **2 a** did not affect T‐cell proliferation. Compounds **2 a** and **3 a** significantly impacted the proliferation of B cell (only 48 and 68 % BrdU incorporation, respectively, compared to the control), whereas **12 a** did not exhibit significant proliferation inhibition of antibody producing cells. Thus, **3 a** affected both B‐ and T‐cell proliferation, whereas **2 a** and **12 a** impaired the proliferative capacity of only one type of immune cells; B cells for **2 a** (humoral immunity) and T cells for **12 a** (cellular immunity).

**Figure 10:**
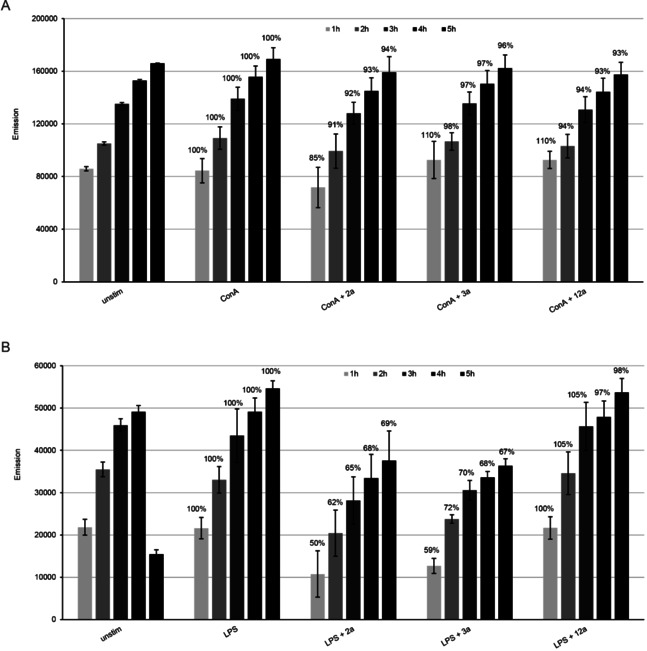
Determination of cytotoxic effects of **2 a**, **3 a** and **12 a** in A) ConA‐ and B) LPS‐induced splenocytes by alamarBlue assay. Bars represent the mean emission of four replicates ± standard deviation. In both experiments and for each time point, 100 % metabolic activity was attributed to the control sample (splenocytes induced with ConA or LPS), then the percentage of metabolic activity in relation to the controls was calculated, and is indicated in the graph for each sample. Significance with *p* <0.001 was observed between LPS and LPS+**2 a**, and LPS+**3 a** for all five time points.

A reduction in the capacity of T and B cells to respond to external signals by proliferative responses could also indicate a potential risk of impaired immunity. However, a reduction in cellular proliferation does not automatically imply a reduced metabolic activity or reduction in cellular viability. Thus, in addition to proliferative responses of B and T cells upon LPS and ConA stimulation, we also assessed the effects of **2 a**, **3 a** and **12 a** on the viability of splenocytes employing the alamarBlue assay, which allows, similar to what we have tested in HFF, to quantify metabolic activity (Figure 10). Measurements taken each hour during the first 5 h after adding the substrate resazurin showed that none of the compounds impaired the metabolic activity of ConA‐stimulated T cells, but compounds **2 a** and **3 a** significantly impaired the viability of B cells upon stimulation with LPS. **12 a** did not show any interference in the metabolic activity of either B or T cells. The lack of viability impairment of splenocytes due to exposure with **12 a** observed here indicates that this compound is a promising drug candidate for future *in vivo* studies in the mouse model, to combat *T. gondii* infection without impairing the cellular and humoral immune response.

**Figure 11:**
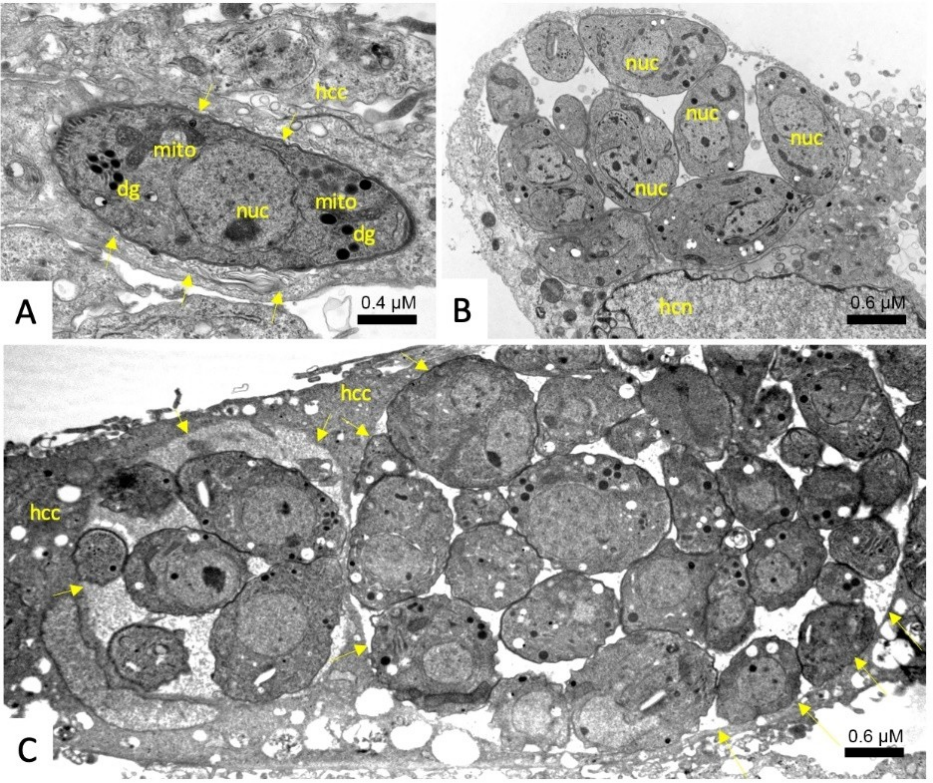
TEM of untreated *T. gondii*‐β‐gal tachyzoites fixed at A) 6, B) 24, and C) 48 h post infection. (A) shows a single tachyzoite, located within a PV that is delineated by a PVM (arrows). (B) Proliferation of tachyzoites takes place within the vacuole, which occupies a substantial part of the host‐cell cytoplasm. (C) shows two neighboring PVs, delineated by arrows, located within a HFF host cell, both containing numerous newly formed tachyzoites. Note the mitochondrion (mito) with an electron dense matrix in (A); nuc=tachyzoite nucleus, dg=dense granule, hcc=host cell cytoplasm; hcn=host cell nucleus.

**Figure 12:**
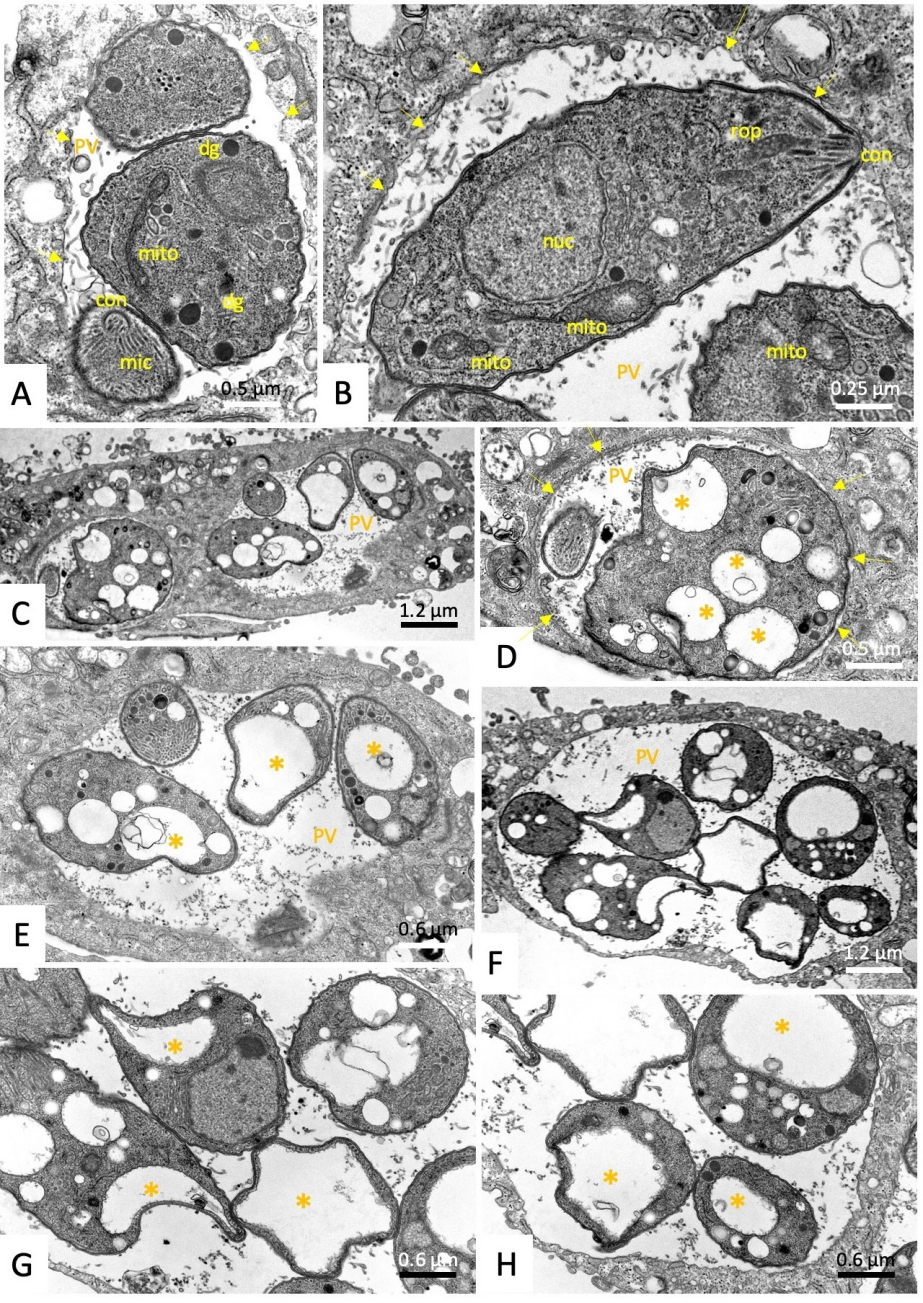
TEM of *T. gondii*‐β‐gal tachyzoites treated with 500 nM of **12 a** for A), B) 6, C)–E) 24, and F)–H) 48 h. No alterations (A) or only very slight changes in the electron dense mitochondrial matrix (mito in (B)) are detected after 6 h. (C)–(E) show that alterations were much more pronounced after 24 h of treatment; (C) is a low‐magnification overview, (D) and (E) represent distinct parts of (C) shown at higher magnification. (F) is a low‐magnification view of a PV of tachyzoites treated for 48 h, (G) and (H) are high magnifications. Note the increased vacuolization (marked with *) and the absence of any mitochondrial matrix after 24–48 h of treatment. con=conoid; rop=rhoptries; mic=microneme; arrows point towards the PVM.

The ultrastructural changes induced by compounds **12 a** (IC_50_ of 0.105 μM against *T. gondii*, HFF viability of 58 % at 2.5 μM) and **17 a** (IC_50_ of 0.243 μM against *T. gondii*, HFF viability of 24 % at 2.5 μM) were further studied by TEM. HFF monolayers were infected with *T. gondii* β‐gal tachyzoites and after 24 h of drug treatment (500 nM of each compound, a concentration that did not notably affect the host cell) were initiated. Samples were fixed and processed after 6, 24, and 48 h. Untreated control cultures are shown in Figure 11. Part A shows a sample fixed 6 h post‐invasion, B and C were fixed 36 and 60 h post‐invasion, respectively, and the increase in number of parasites illustrates the proliferation that takes place within the host cell. Once invaded, *T. gondii* tachyzoites are localized within a parasitophorous vacuole (PV), surrounded by a parasitophorous vacuole membrane (PVM), which is essentially derived from host cell‐surface membrane modified by the parasite following invasion. The mitochondrion exhibits a characteristic electron‐dense matrix containing numerous cristae. Tachyzoites treated with 500 nM **12 a** are shown in Figure 12. At 6 h after initiation of treatment, parasites do not exhibit massive alterations, however, PVs usually contained only 1–3 tachyzoites. The PVM was still clearly discernible, and the secretory organelles such as rhoptries, micronemes, and dense granules, as well as the mitochondria remained largely unaltered (Figure 12 A, B). At 24 h, first ultrastructural changes were noted within the mitochondrial matrix, which started to lose its characteristic electron‐dense matrix. These mitochondrial alterations became progressively more pronounced at 24 (Figure 12 D and E) and 48 h (Figure 12 F, G), resulting in tachyzoites that were completely devoid of a mitochondrion, but exhibited large, seemingly empty, vacuoles instead. Although it is not clear whether these effects were reversible, it is conceivable that this extensive vacuolization would eventually lead to parasite death. Similar results were seen for **17 a**, (Figure S10), although the effects after 24 and 48 h were slightly less pronounced. Overall, these findings mirror previously reported structural alterations induced by ruthenium complexes reported in *T. gondii*, *N. caninum* and in *T. brucei*, and in the latter it was recently shown that active complexes strongly impaired the mitochondrial membrane potential;^23^, ^24^, ^25^ this indicates that ruthenium complexes interfere in the energy metabolism of these parasites. However, although oxidative phosphorylation resulting in the generation of ATP is the major function of mitochondria, these organelles are also involved in other crucial processes, including cell‐cycle regulation, tRNA and protein import, mitochondrial protein translation, alternative oxidase, acetate production for cytosolic and mitochondrial fatty acid biosynthesis, amino acid metabolism, and calcium homeostasis, they are also involved in the steps leading to programmed cell death. How, and to what extent, these compounds actually target mitochondrial functions or whether other targets are also involved remain to be elucidated

In order to assess the potential use of the new conjugates coumarin–diruthenium unit as cellular trackable probes fluorescence microscopy assays were performed of HFF cells treated with either 20 μM **Dye2**‐CO_2_H or 20 μM of **12 a**, both counterstained with an antibody directed against tubulin and NucRed, a fluorescent nuclear label (see Results and Experimental in the Supporting Information). However, whereas antitubulin readily stained the cytoskeleton of HFF and NucRed indicated the nucleus, no labeling could be observed with either **Dye2**‐CO_2_H or **12 a**. In order to obtain trithiolato dinuclear ruthenium conjugates suitable to be used as theranostics, the attachment of other fluorophores should be considered.

## Conclusion

A library of 13 new trithiolato‐bridged dinuclear ruthenium(II)⋅arene organometallic conjugates in which coumarin moieties were anchored to the bridged thiol(s), has been designed and synthesized.

Irrespective to the structural variations considered, for all conjugates organometallic unit: coumarin an almost complete loss of fluorescence efficacy was observed. However, the nature of the fluorophore, the type of bonding, the presence and length of a linker between the coumarin dye and the dinuclear ruthenium(II) moiety, and the number of dye units influenced the biological properties of these compounds. These modifications also affect the toxicity of these compounds against human fibroblasts, and impact the activity against the apicomplexan parasite *T. gondii*, grown in these cells *in vitro*. For selected compounds (**2 a**, **3 a** and **12 a**), applied at their *T. gondii*‐IC_50_, the effect on the proliferative responses of splenocytes upon ConA (for T cell) and LPS (for B cell) stimulation was investigated, and the influence on the viability/metabolic activity of B and T cells *in vitro* was assessed. That the IC_50_ values range from 105 to 735 nM and nine compounds displayed lower IC_50_ values than the standard drug pyrimethamine suggests that these class of compounds is promising. In particular, compound **12 a** did not affect the metabolic activity of B or T cells *in vitro*, is therefore not expected to impair immunity, and thus represents a promising compound for future *in vivo* assessment in toxoplasmosis mouse models.

## Experimental Section

**General**: The chemistry experimental part, with full description of experimental procedures and characterization data for all compounds, is presented in the Supporting Information.

**Crystal‐structure determination**: A crystal of C_39_H_45_Cl_3_O_3_Ru_2_S_3_ was mounted in air at ambient conditions. All measurements were made on a RIGAKU Synergy S area‐detector diffractometer^63^ using mirror optics monochromated Cu_Kα_ radiation (*λ*=1.54184 Å).^64^ The unit cell constants and an orientation matrix for data collection were obtained from a least‐squares refinement of the setting angles of reflections in the range 6.266°<2*θ*<155.814°. A total of 4310 frames were collected by using ω scans, with 0.05 s exposure time, a rotation angle of 0.5° per frame, a crystal‐detector distance of 31.0 mm, at *T*=173(2) K.

Data reduction was performed using the CrysAlisPro^63^ program. The intensities were corrected for Lorentz and polarization effects, and an absorption correction based on the multiscan method by using SCALE3 ABSPACK in CrysAlisPro^63^ was applied. Data collection and refinement parameters are given in Table S1.

The structure was solved by direct methods using SHELXT,^65^ which revealed the positions of all non‐hydrogen atoms of the title compound. All non‐hydrogen atoms were refined anisotropically. H‐atoms were assigned in geometrically calculated positions and refined using a riding model where each H‐atom was assigned a fixed isotropic displacement parameter with a value equal to 1.5Ueq of its parent atom (for methyl groups.

Refinement of the structure was carried out on *F* ^*2*^ using full‐matrix least‐squares procedures, which minimized the function Σ*w*(*F*_o_^2^−*F*_c_^2^)^2^. The weighting scheme was based on counting statistics and included a factor to down weight the intense reflections. All calculations were performed using the SHELXL‐2014/7^66^ program in OLEX2.^67^

**Photophysical measurements**: UV/Vis spectra were recorded on a Thermo Scientific Evolution 201 UV/Vis spectrophotometer. Fluorescence emission spectra were measured on an Agilent Cary Eclipse fluorescence spectrophotometer.

The UV/Vis absorption spectra of compounds **5**–**17 a**/**b**, **20** and **22** were recorded in the range 200–1100 nm at room temperature by using solutions of 1, 2, 5 and 10 μM in CHCl_3_ and in EtOH. Emission spectra were recorded in the range 405–650 nm after excitation at 405 nm (excitation and emission filters: auto, excitation and emission slit=2.5 nm), using 1, 2, 5 and 10 μM solutions in CHCl_3_ and in EtOH. All the experiments were studied at r.t., the solvent absorption was deducted as background.

**Determination of quantum yields**: Relative quantum yields for solutions in CHCl_3_ and EtOH at rt were calculated by a relative method according to Equation (1) and rhodamine 6G (*Φ*_F_=0.75 in CHCl_3_, *Φ*_F_=0.94 in EtOH) as standard.^58^, ^68^ The absorption of rhodamine 6G was adjusted to the same value (abs <1) as that of fluorescent molecules. Excitation was chosen at 405 nm; the emission spectra were corrected and integrated for the area under the emission curve.

(1) $$\Phi_{F}\left(x\right)=\frac{A_{s}}{A_{x}}\times \frac{F_{x}}{F_{s}}{\times \left(\frac{n_{x}}{n_{s}}\right)}^{2}\times \Phi_{F}\left(s\right)\;$$

where *A* is the absorbance at the excitation wavelength, *F* is the integration of emission intensity, *n* is the refractive index of the solvents (at 20 °C) used in measurements (*n*=1.446 for CHCl_3_, *n*=1.3611 for EtOH), and the subscripts *s* and *x* represent standard and unknown, respectively.

(2) Δλ=λemmax-λabsmax

Stokes shifts were calculated from Equation (2) as the difference between the values of maxima of the intense bands in the fluorescence and absorption spectra

### Experimental biology

*In vitro culture of parasites and host cells*: If not stated otherwise, all tissue culture media were purchased from Gibco‐BRL, and biochemical reagents were from Sigma‐Aldrich). Human foreskin fibroblasts (HFF) were maintained in DMEM containing 10 % fetal calf serum (FCS; Gibco‐BRL) and antibiotics as described earlier.^10^ *T. gondii* β‐gal (transgenic *T. gondii* RH expressing the β‐galactosidase gene from *E. coli* 69 were maintained in HFF cells, and were isolated and separated from their host cells as described.^10^

*In vitro assessment of drug efficacy*: To study the effects of compounds against *T. gondii* tachyzoites *in vitro*, 1 mM stock solutions of complexes were prepared in DMSO, and stored at −20 °C. For assessment of drug efficacy against *T. gondii* tachyzoites, parasites were isolated and assays were performed using HFF as host cells as previously described.^10^ In short, 5×10^3^ HFF cells per well were grown to confluence in a 96‐well plate in phenol‐red free culture medium at 37 °C with 5 % CO_2_. Cultures were infected with freshly isolated *T. gondii* β‐gal tachyzoites (1×10^3^ per well), and drugs were added concomitantly with infection. Initial assessments of drug efficacy were done by exposing parasite cultures to 0.1 and 1 μM of each compound for a period of three days, or 0.1 % DMSO was added as a control. For IC_50_ determinations, compounds were added at 6 concentrations: 0.03, 0.06, 0.12, 0.25, 0.5, and 1 μM. After three days of incubation at 37 °C, 5 % CO_2_, medium was removed, and cell cultures were permeabilized by using 90 μL PBS containing 0.05 % Triton‐X‐100. After addition of 10 μL of 5 mM chlorophenol red‐β‐d‐galactopyranoside (CPRG; Roche Diagnostics) dissolved in PBS, the absorption shift was measured at 570 nm wavelength at various time points on a VersaMax multiplate reader (Bucher Biotec, Basel, Switzerland). For the initial screening at 0.1 and 1 μM, the activity, measured as the release of chlorophenol red over time, was calculated as percentage from DMSO control, which represented 100 % of *T. gondii* β‐gal growth. For the IC_50_ assays, the activity measured as the release of chlorophenol red over time was proportional to the number of live parasites down to 50 per well as determined in pilot assays. IC_50_ values were calculated after the logit‐log‐transformation of relative growth and subsequent regression analysis. All calculations were performed using the corresponding software tool contained in the Excel software package (Microsoft).

Cytotoxicity assays on uninfected confluent HFF were performed also in 96 well plates by exposing HFF to a concentration range of 0.1, 1, and 2.5 μM of each compound, and assessment of the viability by alamarBlue assay as described.^70^

**Isolation of murine splenocytes**: Female BALB/c mice were purchased from Charles River Laboratories (Sulzfeld, Germany) and were maintained in a common room under controlled temperature and a 14 h dark/10 h light cycle according to the standards set up by the animal welfare legislation of the Swiss Veterinary Office. The experimental protocol was approved by the Commission for Animal Experimentation of the Canton of Bern, Switzerland (Animal license no. BE101/17). Mice were euthanized with isoflurane and CO_2_, and spleens were aseptically removed from euthanized mice. Single‐cell suspension was prepared by gently mincing spleen tissue and passing it through sterile 40 μm cell‐strainer. Erythrocytes were depleted from cell suspension using RBC lysis buffer (Invitrogen, Thermo Fisher Scientific) for 5 min. The viability of isolated cells was determined using Trypan Blue dye exclusion test, and preparations were only used when >99 % of viable cell were counted.in a Neubauer hemocytometer. The spleen cell preparation containing T cells, dendritic cells, B cells and macrophages, was then suspended in RPMI 1640 medium including 10 % FCS, 0.05 mM 2‐mercaptoethanol, 2 mM l‐glutamine, and 100 U of penicillin plus 50 mg of streptomycin per mL. Cell suspensions were distributed in polystyrene 96 well flat bottom sterile plastic plates (Greiner Bio‐One; HuberLab) at 2×10^5^ cells/100 μL/well.

**Splenocyte proliferation assay**: Isolated primary splenocytes were either left unstimulated or were stimulated with ConA (5 μg/mL), lipopolysaccharide (LPS, 10 μg/mL), ConA plus compound or LPS plus compound. Compounds **2 a**, **3 a** and **12 a** were added at their respective IC_50_ value. Experiments were performed in quadruplicate wells, 200 μL/well, and cultures were maintained in a 37 °C humidified chamber containing 5 % CO_2_ for a total incubation period of 72 h. Proliferative responses of splenocytes were measured using a BrdU cell proliferation kit (QIA58, Merck Millipore). Briefly, BrdU label was added to the cultures 18 h prior the end of the incubation period. Incorporated BrdU into the newly synthetized DNA was measured by ELISA using anti‐BrdU monoclonal antibody. Immediately after stopping the reaction, the absorbance was measured at 450/540 nm, in an EnSpire multilabel reader (Perkin Elmer, Waltham). Data are presented as mean±SD for the indicated numbers. Data comparisons between groups were examined using a student's t‐test (significant when *p* <0.01).

**Determination of cytotoxic effects in splenocytes**: To determine whether compounds **2 a**, **3 a** and **12 a** exhibit an effect on the metabolism of ConA‐ and LPS‐induced splenocytes, the alamarBlue assay was performed. Isolated splenocytes were seeded in 96‐well plates at a density of 1×10^6^ cells/mL with a final volume of 100 μL/well. Cells were either left unstimulated or stimulated with ConA (5 μg/mL), LPS (10 μg/mL), ConA plus compound or LPS plus compound. Each compound was tested at its IC_50_ against *T. gondii* β‐gal, in quadruplicate wells at a volume of 200 μl/well. Cultures were maintained in a 37 °C humidified chamber containing 5 % CO_2_ for a total incubation period of 72 h. Resazurin (0.1 mg/mL) was added, and the fluorescence intensity was measured at 530 nm excitation wavelength and a 590 nm emission wavelength using an EnSpire multilabel reader (Perkin Elmer). Measurements were done at different time points *T*=0, 1, 2, 3, 4, or 5 h. Differences were calculated by subtracting T0 values from each time point. Data are presented as mean of emission±SD for the indicated numbers. Data comparisons between groups were examined using a student's t‐test (significant when *p* <0.001).

**Transmission electron microscopy**: HFF (5×10^5^ per inoculum) grown to confluence in T‐25 tissue culture flasks were infected with 10^5^ *T. gondii* Me49 tachyzoites, and 500 nM of **12 a** or **17 a** were added at 24 h post‐infection. After 6, 24 or 48 h, cells were harvested by using a cell scraper, and placed into the primary fixation solution (2.5 % glutaraldehyde in 100 mM sodium cacodylate buffer pH 7.3) for 2 h. Specimens were then washed twice in cacodylate buffer and were post‐fixed in 2 % OsO_4_ in cacodylate buffer for 2 h, followed by washing in water, prestaining in saturated uranyl acetate solution, and step wise dehydration in ethanol. They were then embedded in Epon 812‐resin, and processed for TEM as described.^24^ Specimens were viewed on a CM12 transmission electron microscope operating at 80 kV.

Deposition number 1985451 (for C_39_H_45_Cl_3_O_3_Ru_2_S_3_ (**21**)) contains the supplementary crystallographic data for this paper. These data are provided free of charge by the joint Cambridge Crystallographic Data Centre and Fachinformationszentrum Karlsruhe Access Structures service www.ccdc.cam.ac.uk/structures.

**Abbreviations**: IC_50_: concentration at which 50 % of the tachyzoite growth is inhibited (median inhibitory concentration, a measure of tachyzoite inhibition). *T. gondii* β‐gal: transgenic *Toxoplasma gondii* tachyzoites constitutively expressing *Escherichia coli* β‐galactosidase, grown in HFF monolayers.

## Conflict of interest

The authors declare no conflict of interest.

## Supporting information

As a service to our authors and readers, this journal provides supporting information supplied by the authors. Such materials are peer reviewed and may be re‐organized for online delivery, but are not copy‐edited or typeset. Technical support issues arising from supporting information (other than missing files) should be addressed to the authors.

Supplementary
