# Supplementary material for: Coumarin‐Tagged Dinuclear Trithiolato‐Bridged Ruthenium(II)⋅Arene Complexes: Photophysical Properties and Antiparasitic Activity
Source: Chembiochem. 2020 Jun 16;21(19):2818–35. doi: 10.1002/cbic.202000174 (PMC7586963; doi:10.1002/cbic.202000174)
Supplement: Supplementary file 1 — Supplementary [file CBIC-21-2818-s001.pdf]

ChemBioChem

Supporting Information

Coumarin-Tagged Dinuclear Trithiolato-Bridged Ruthenium(II)·Arene Complexes: Photophysical Properties and Antiparasitic Activity

Oksana Desiatkina, Emilia Păunescu,* Martin Mösching, Nicoleta Anghel, Ghalia Boubaker, Yosra Amdouni, Andrew Hemphill,* and Julien Furrer*

Contents of supporting information	Page
**X-ray crystallography**	S3
**Table S1**. Crystal data and structure refinement for **21**.	S3
**Table S2.** Key bond lengths (Å) and angles (°) of **21**.	S4
**Table S3.** Intramolecular H-bonding interactions for complex **21**.	S4
**Figure S1.** Packing in the crystal of **21** dictated by the intermolecular H-bonding interactions.	S5
**Stability in DMSO-d_6**	S6
**Figure S2.** ^{1}H NMR Spectra of **2a**, **11** and **12a** recorded in DMSO-d_6 at 25 °C.	S6
**Figure S3.** ^{1}H NMR spectra of **3a**, **13** and **14a** recorded in DMSO-d_6 at 25 °C.	S7
**Figure S4.** ^{1}H NMR spectra of **4a**, **15**, **16a** and **17a** recorded in DMSO-d_6 at 25 °C.	S8
**Photophysical Characterization**	S9
**Table S4.** Photophysical data of compounds **5-17a/b**, **20** and **22** in EtOH at r.t.	S9
**Figure S5.** UV-Vis absorption (left) and emission spectra (right) of rhodamine 6G, **Dye2-CO$_2$H**, intermediate **7**, **10** and the corresponding ester **12a** and amide **14a**, **17a** conjugates, at 10 µM in EtOH.	S10
**Figure S6.** UV-Vis absorption (left) and emission spectra (right) of rhodamine 6G, **Dye2-CO$_2$H**, intermediate **7**, **10** and the corresponding ester **12b** and amide **14b**, **17b** conjugates, at 10 µM in EtOH.	S10
**Figure S7.** UV-Vis absorption (left) and emission spectra (right) of rhodamine 6G, **Dye1-CO$_2$H** and ester conjugates **11**, **20**, **22**, at 10 µM in EtOH.	S11
**Biological activity**	S12
**Table S5.** Primary efficacy/cytotoxicity screening of the non-modified trithiolato di-ruthenium compounds and corresponding coumarin conjugates.	S12
**Figure S8.** IC$_{50}$ values in *T. gondii* β-gal (µM) and HFF viability at 2.5 µM determined for selected trithiolato-bridged dinuclear ruthenium(II)-arene complexes and pyrimethamine.	S13
**Figure S9.** Dose response curves for the selected compounds for which the IC$_{50}$ against *T. gondii* was determined. Bars represent standard deviations. Six concentrations between 1 and 0.0312 µM were tested, each in six replicate wells.	S14
**Figure S10.** TEM of *T. gondii*-β-gal tachyzoites treated with 500 nM of **17a** at 6 h, 24 h and 48 h after initiation of treatment.	S15
**Figure S11.** Fluorescence microscopy of HFF treated with 20 µM **Dye2-CO$_2$H** or 20 µM of **12a**.	S16
**Experimental**	S17
Chemistry	S17
General	S17
Synthesis and characterization	S18
Biology	S42
Structures of compounds	S43
**References**	S46

X-ray crystallography

**Table S1.** Crystal data and structure refinement for **21 (19JF005_M029f(21-30))**	
**Compound**	**21 (19JF005_M029f(21-30))**
Formula	$C_{39}H_{45}Cl_3O_3Ru_2S_3$
F.W. ($g{\cdot}mol^{-1}$)	966.42
Temperature (K)	173.01(10)
Crystal system	monoclinic
Space group	$P2_1/c$
a (Å)	10.17797(4)
b (Å)	21.05828(9)
c (Å)	19.56601(8)
α (°)	90
β (°)	103.8437(4)
γ (°)	90
V (Å^3)	4071.77(3)
Z	4
D_{calc} ($g{\cdot}cm^{-3}$)	1.576
μ (mm^{-1})	9.537
F(000)	1960.0
Crystal size (mm^3)	0.25 × 0.15 × 0.1
Radiation	CuKα (λ = 1.54184)
2Θ range for data collection (°)	6.266 to 155.814
Index ranges	
h	-12 / 12
k	-26 / 24
l	-24 / 24
Reflns. collected	64148
Independent reflns.	8560 [R_{int} = 0.0727, R_{sigma} = 0.0347]
Data/restraints/parameters	8560/0/460
GoodF^2	1.037
R1 [I>=2σ (I)]	0.0383
wR_2	0.0993
R1 [all data]	0.0398
wR_2	0.1006
Largest diff. peak/hole (Å^{-3})	0.95 and -1.55 e

**Table S2.** Comparison of key bond lengths (Å) and angles (°) of **21** (**19JF005_M029f(21-30)**).	
	**19JF005_M029f(21-30)**
**Ru-S**	Ru(1)-S(1) 2.4091(7)<br>Ru(1)-S(2) 2.3834(7)<br>Ru(1)-S(3) 2.4006(7)<br>Ru(2)-S(1) 2.4182(7)<br>Ru(2)-S(2) 2.3688(7)<br>Ru(2)-S(3) 2.4117(7)
**Ru-*η*6**	Ru(1)-cent(C26-C31) 1.691<br>Ru(2)-cent(C9-C14) 1.699
**S-Ru-S**	S(1)-Ru(1)-S(2) 76.18(2)<br>S(1)-Ru(1)-S(3) 76.00(2)<br>S(2)-Ru(1)-S(3) 78.38(2)<br>S(1)-Ru(2)-S(2) 76.27(2)<br>S(1)-Ru(2)-S(3) 75.62(2)<br>S(2)-Ru(2)-S(3) 78.44(2)
**Ru-S-Ru**	Ru(1)-S(1)-Ru(2) 87.59(2)<br>Ru(1)-S(2)-Ru(2) 89.34(2)<br>Ru(1)-S(3)-Ru(2) 87.93(2)
**Ru-cent(S-S-S)-Ru**	Ru(1)-cent(S1-S3)-Ru(2) 176.46
**cent *η*6-cent(S-S-S)-cent *η*6**	cent(C26-C31)-cent(S1-S3)-cent(C9-C14) 175.83

**Table S3.** Intramolecular H-bonding interactions for complex **19JF005_M029f(21-30)**.					
**Compnd.**	**Contact**	**Distance (Å)**			**Angle (°)**
	**D-H···A**	***D*-H**	**H···*A***	***D*···*A***	***D*-H···*A***
**21**	O24-H24…Cl3	0.820	2.325	3.144	177.62
**19JF005_M029f(21-30)**	O7-H7…Cl3	0.820	2.239	3.032	162.66
	O41-H41…Cl3	0.820	2.325	3.102	158.19

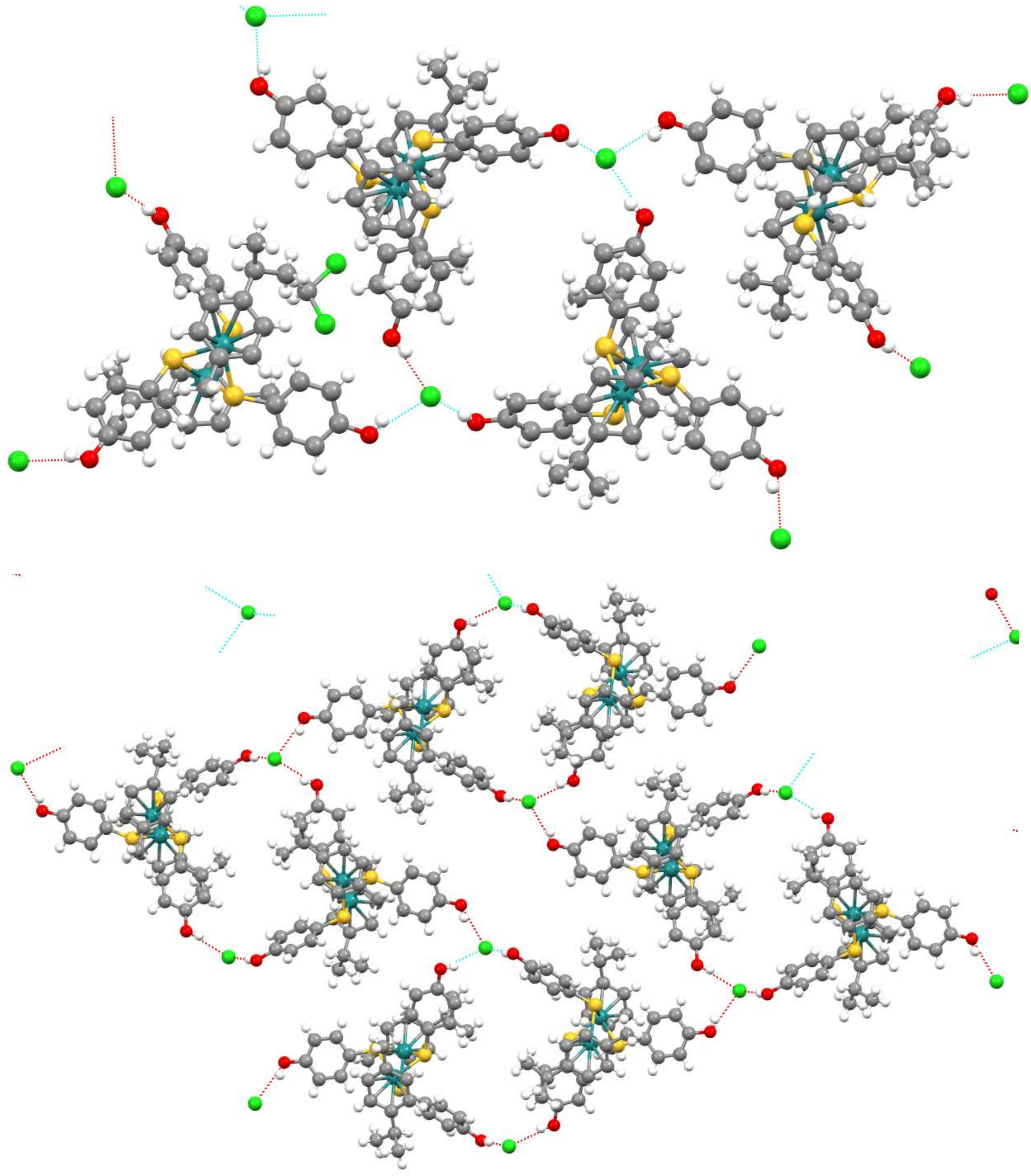

Figure S1. Intermolecular H-bonding interactions in the crystal of **19JF005_M029f(21-30)** with the formation of dimers; four H-bonds interconnect four hydroxy groups from two di-ruthenium complexes via two chorine anion bridges, (contacts D-H···A correspond to O-H···Cl^-, image produced using Mercury CCDC 4.1.2, see bond parameters in Table 2).

Stability in DMSO-d_6

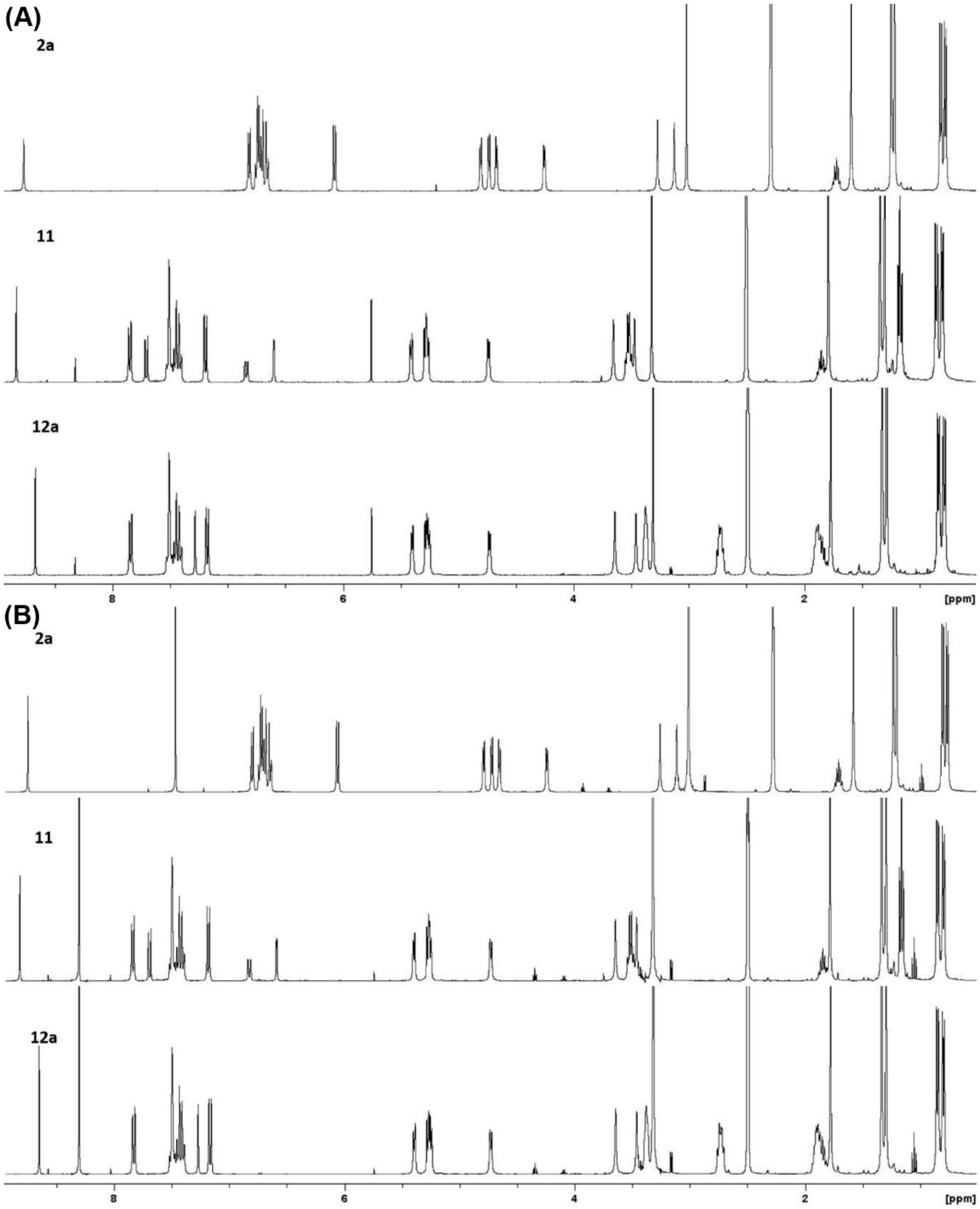

Figure S2. ^{1}H NMR Spectra of **2a**, **11** and **12a** recorded in DMSO-d_6 at 25°C; (A) recorded 5 min after sample preparation, and (B) sample after > 365 days storage at 0-5°C in the dark.

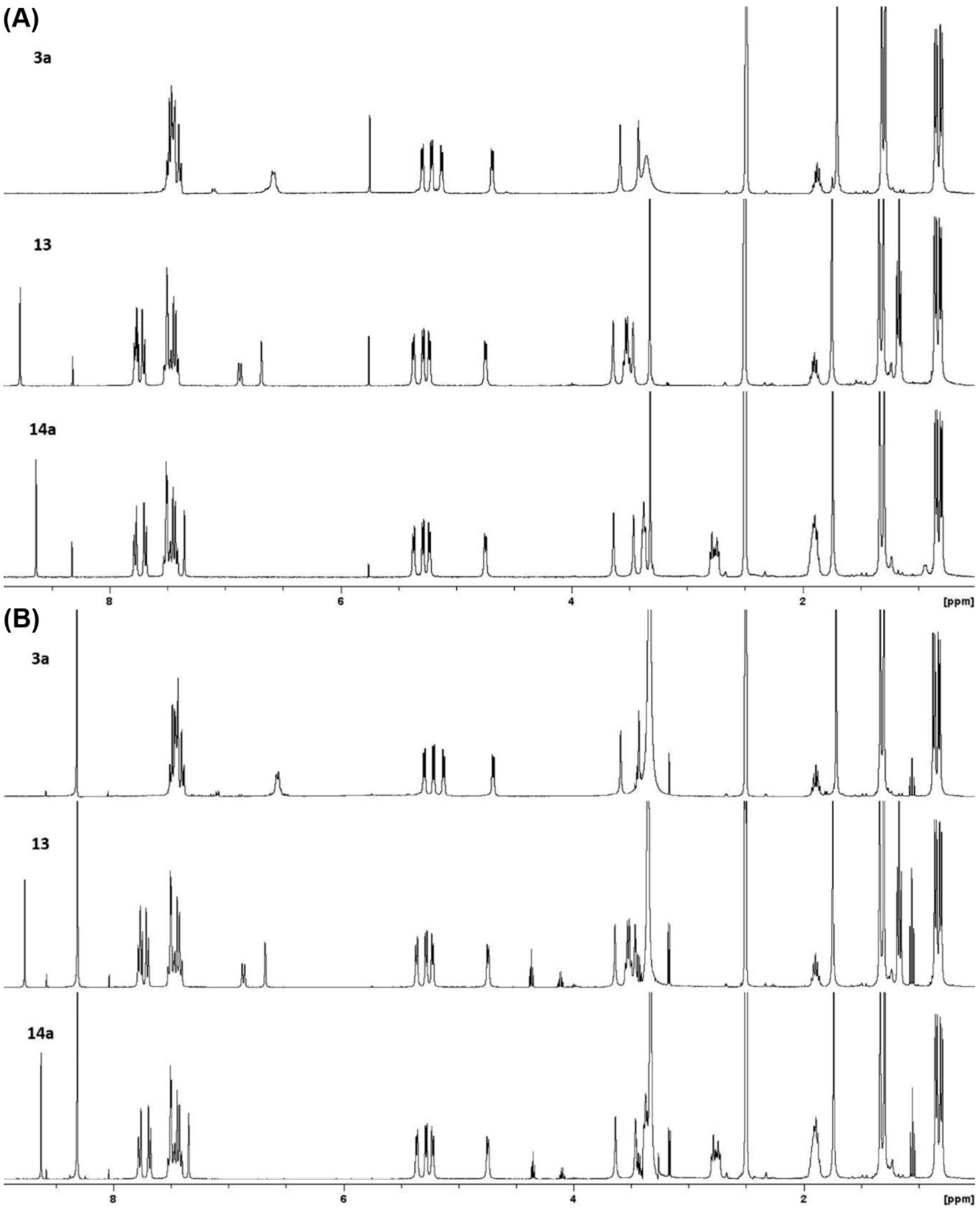

Figure S3. ^{1}H NMR spectra of **3a**, **13** and **14a** recorded in DMSO-d$_6$ at 25°C; (A) recorded 5 min after sample preparation, and (B) sample after > 365 days storage at 0-5°C in the dark.

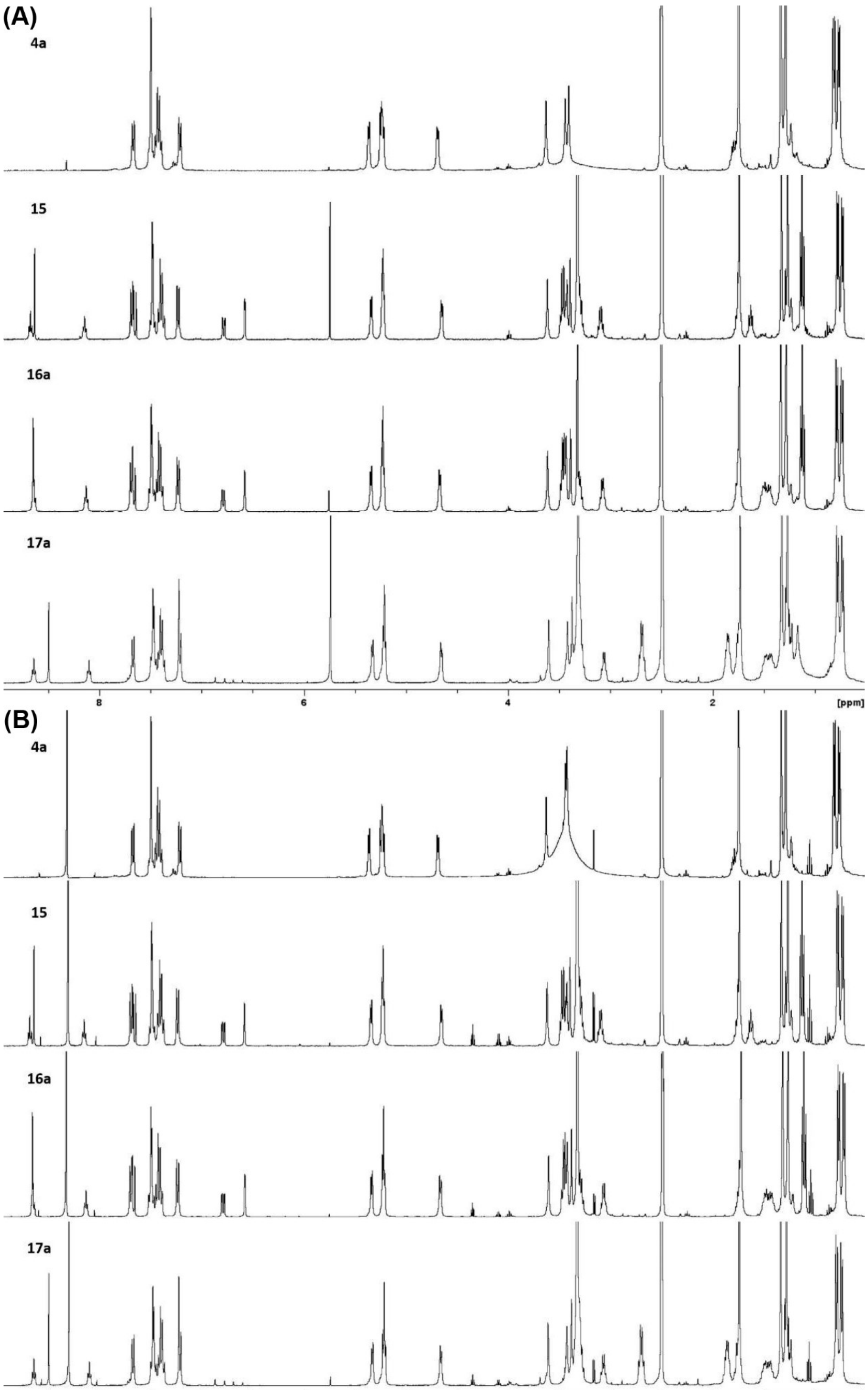

Figure S4. ^{1}H NMR spectra of **4a**, **15**, **16a** and **17a** recorded in DMSO-d$_6$ at 25°C; (A) recorded 5 min after sample preparation, and (B) sample after > 365 days storage at 0-5°C in the dark.

Photophysical Characterization

Table S4. Photophysical data of compounds **5-17a/b**, **20** and **22** in EtOH at r.t..

Compound	λ_{max}^{abs} (nm)	ε ($M^{-1}cm^{-1}$)	λ_{max}^{em} (nm)	$\Delta\lambda$ (nm)	Φ_F (%)
**rhodamine 6G***	529.5	91583.4	555	25.5	94*
**Dye1-CO_2H**	423.5	33814.8	457	33.5	13
**Dye2-CO_2H**	445	30558.6	486	41	140
**5**	417.5	43561.9	464	46.5	12
**6**	417.5	42765.0	466	48.5	12
**7**	435.5	34320.6	480	44.5	130
**8**	420.5	15501.3	468	47.5	11
**9**	419.5	24175.6	466	46.5	11
**10**	437	45453.2	480	43	124
**11**	426.5	53185.9	458	31.5	0.2
**12a**	445	55210.5	481	36	1.7
**12b**	445.5	59061.7	480	34.5	0.6
**13**	436	66782.6	-	-	0
**14a**	453.5	64679.7	489	35.5	0.4
**14b**	453.5	65408.8	485	31.5	1.9
**15**	418.5	36376.6	459	40.5	0.8
**16a**	418.5	39905.5	459	40.5	0.9
**16b**	418.5	32988.9	460	41.5	1.0
**17a**	436	32712.9	479	43	1.8
**17b**	436.5	29334.5	479	42.5	2.4
**20**	428	97373.8	466	38	0.06
**22**	428.5	146466.0	465	36.5	0.1

*Values taken from ref.[1]

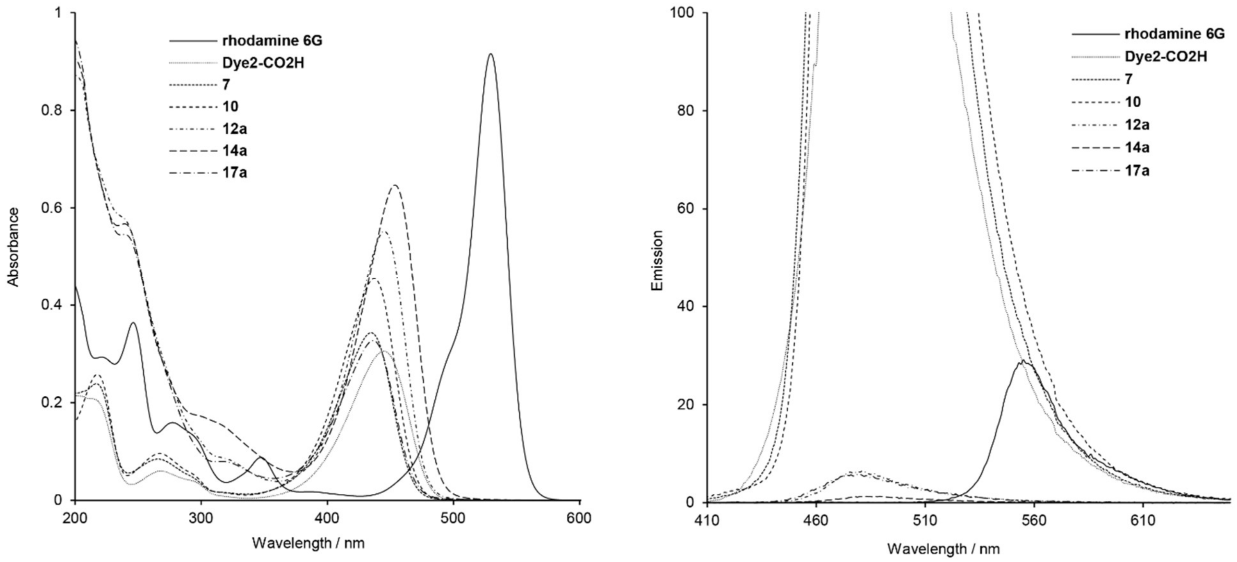

Figure S5. UV-Vis absorption (left) and emission spectra (right) of rhodamine 6G, **Dye2-CO₂H**, intermediate **7**, **10** and the corresponding ester **12a** and amide **14a**, **17a** conjugates, at 10 μM in EtOH.

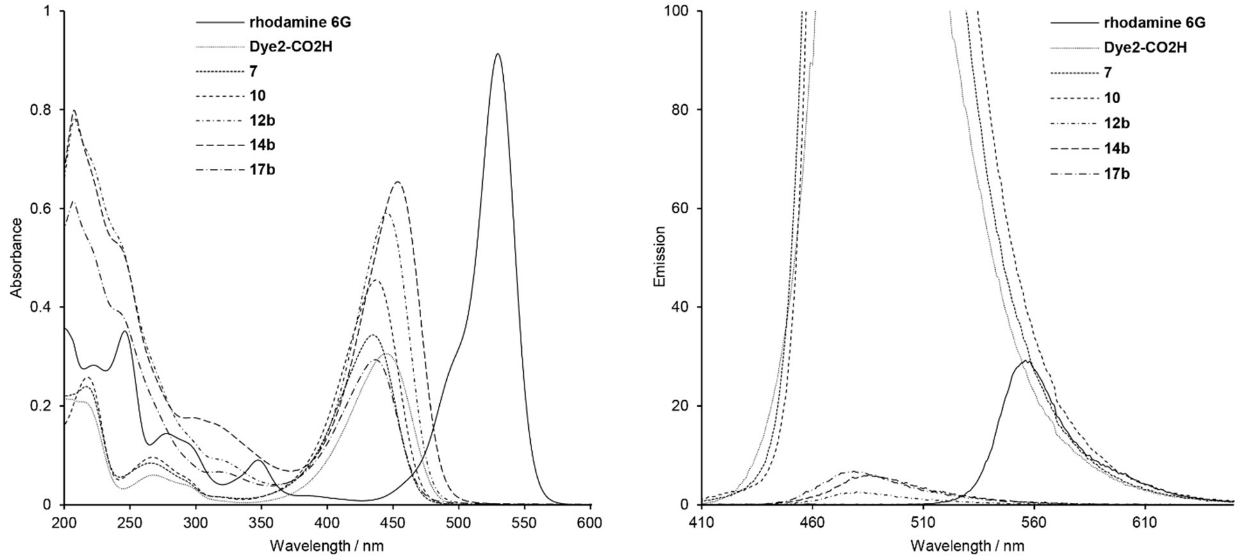

Figure S6. UV-Vis absorption (left) and emission spectra (right) of rhodamine 6G, **Dye2-CO₂H**, intermediate **7**, **10** and the corresponding ester **12b** and amide **14b**, **17b** conjugates, at 10 μM in EtOH.

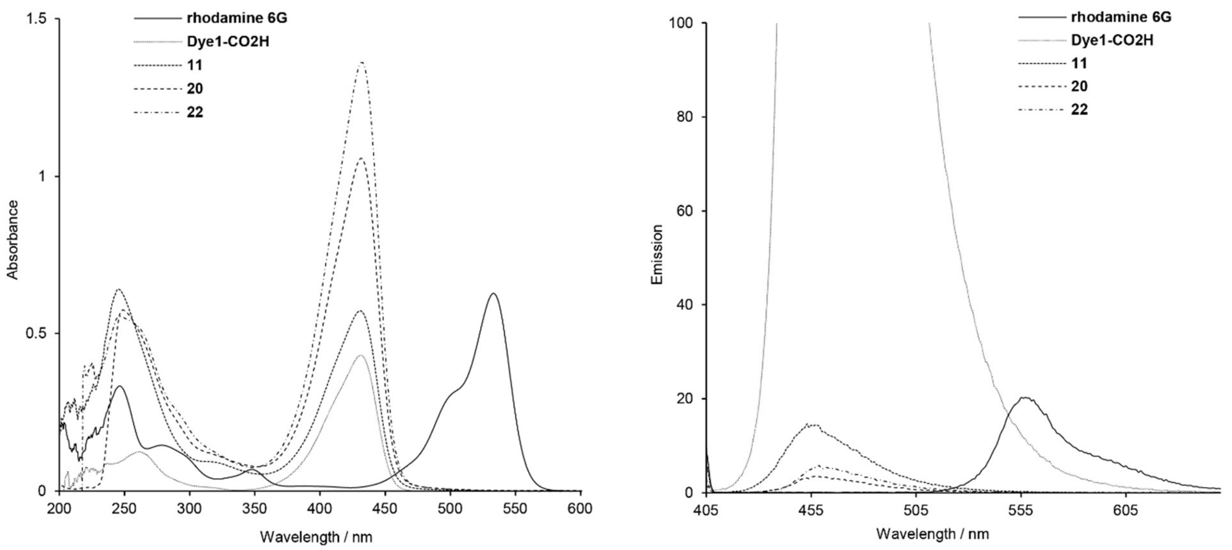

Figure S7. UV-Vis absorption (left) and emission spectra (right) of rhodamine 6G, **Dye1-CO₂H** and ester conjugates **11**, **20**, **22**, at 10 µM in EtOH.

Biological activity

Table S5. Primary efficacy/cytotoxicity screening of the non-modified trithiolato di-ruthenium compounds and corresponding coumarin conjugates. Those compounds selected for determination of IC_{50} values against *T. gondii* β-gal are marked with a *. Symmetric trihydroxy thiolato-bridged dinuclear ruthenium(II)-arene compound **21** was evaluated previously.[2, 3]

**Compound**	**HFF viability (%)**		***T. gondii* β-gal proliferation (%)**	
	**0.1 µM**	**1 µM**	**0.1 µM**	**1 µM**
**Dye1-CO_2H**	103 ± 1	105 ±1	53 ± 12	118 ± 6
**Dye2-CO_2H**	106 ± 1	105 ± 0	69 ± 11	92 ± 6
**2a***	76 ± 6	46 ± 6	66 ± 14	2 ± 0
**2b***	112 ± 7	66 ± 4	101 ± 1	0 ± 0
**3a***	74 ± 2	48 ± 1	57 ± 1	2 ± 0
**3b***	115 ± 1	58 ± 2	59 ± 7	0 ± 0
**4a**	93 ± 4	87 ± 1	114 ± 15	110 ± 32
**4b**	103 ± 3	93 ± 6	102 ± 5	102 ± 3
**5**	118 ± 10	88 ± 10	131 ± 3	118 ± 22
**6**	98 ± 8	82 ± 4	137 ± 9	125 ± 16
**7**	95 ± 9	96 ± 6	132 ± 6	148 ± 5
**8**	96 ± 9	85 ± 6	117 ± 10	114 ± 13
**9**	125 ± 20	111 ± 4	114 ± 4	127 ± 4
**10**	124 ± 5	69 ± 9	101 ± 8	113 ± 7
**12a***	93 ± 14	85 ± 3	6 ± 0	1 ± 0
**12b***	100 ± 0	58 ± 5	97 ± 0	2 ± 0
**13**	99 ± 14	42 ± 8	100 ± 10	70 ± 11
**14a**	87 ± 3	79 ± 8	123 ± 8	119 ± 3
**14b***	105 ± 5	70 ± 1	88 ± 1	3 ± 0
**15**	105 ± 9	45 ± 6	112 ± 19	1 ± 0
**16a***	109 ± 4	52 ± 15	111 ± 26	0 ± 0
**16b***	100 ± 9	79 ± 6	99 ± 2	6 ± 1
**17a***	92 ± 4	53 ± 7	13 ± 12	1 ± 0
**17b***	96 ± 1	56 ± 5	100 ± 4	1 ± 0
**19***	62 ± 8	56 ± 7	3 ± 1	2 ± 0
**20***	99 ± 0	80 ± 1	136 ± 2	2 ±0
**22**	111 ± 1	101 ± 1	99 ±0	88 ±2

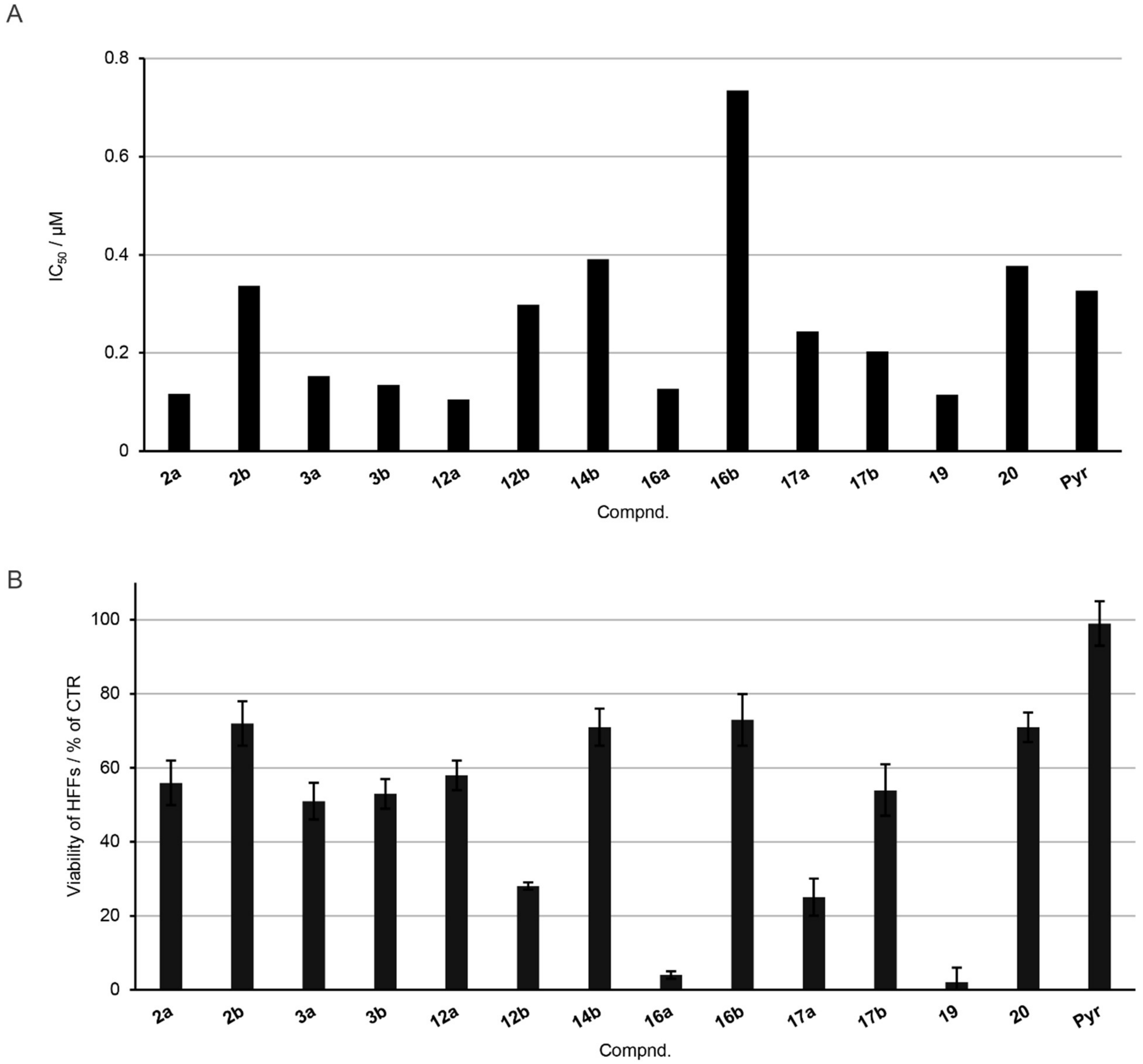

Figure S8. IC_{50} values on *T. gondii* β-gal (A) and HFF viability at 2.5 µM (B) determined for selected trithiolato-bridged dinuclear ruthenium(II)-arene complexes and pyrimethamine. *T. gondii* β-gal proliferation and HFF viability were quantified by β-galactosidase and Alamar Blue assay, respectively.

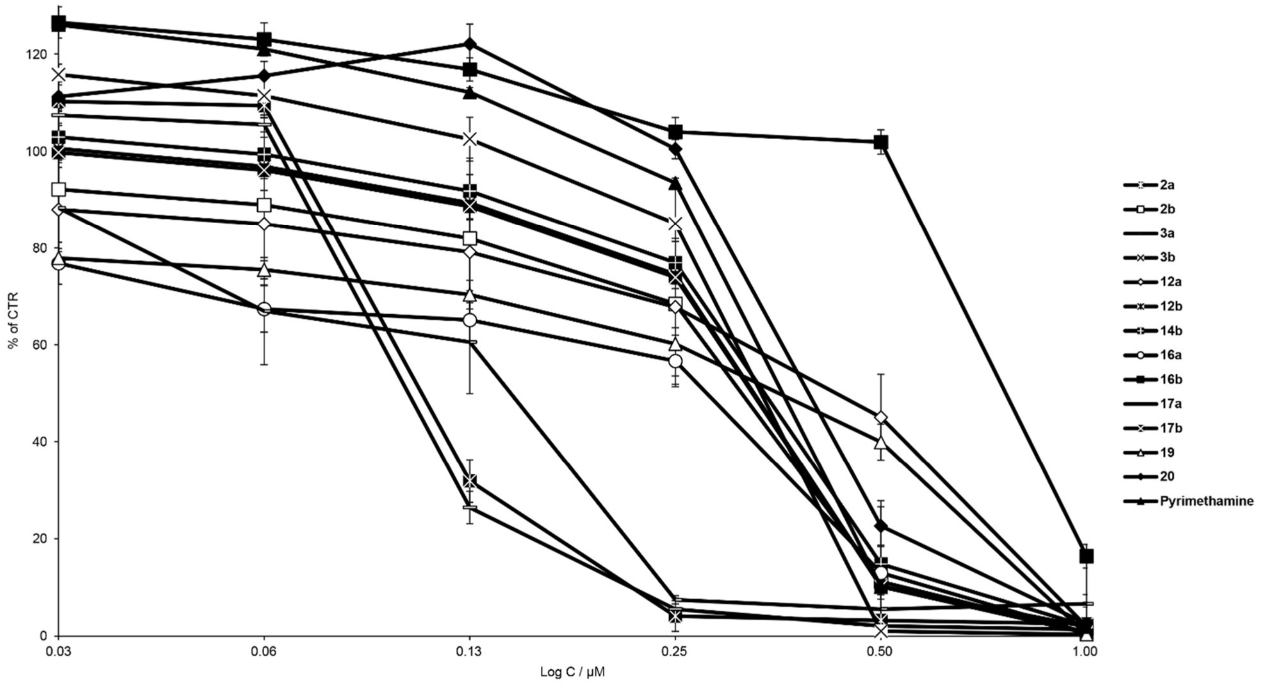

Figure S9. Dose response curves for the selected compounds for which the IC_{50} against *T. gondii* β-gal was determined. Bars represent standard deviations. Six concentrations between 1 µM and 0.0312 µM were tested, each in six replicate wells.

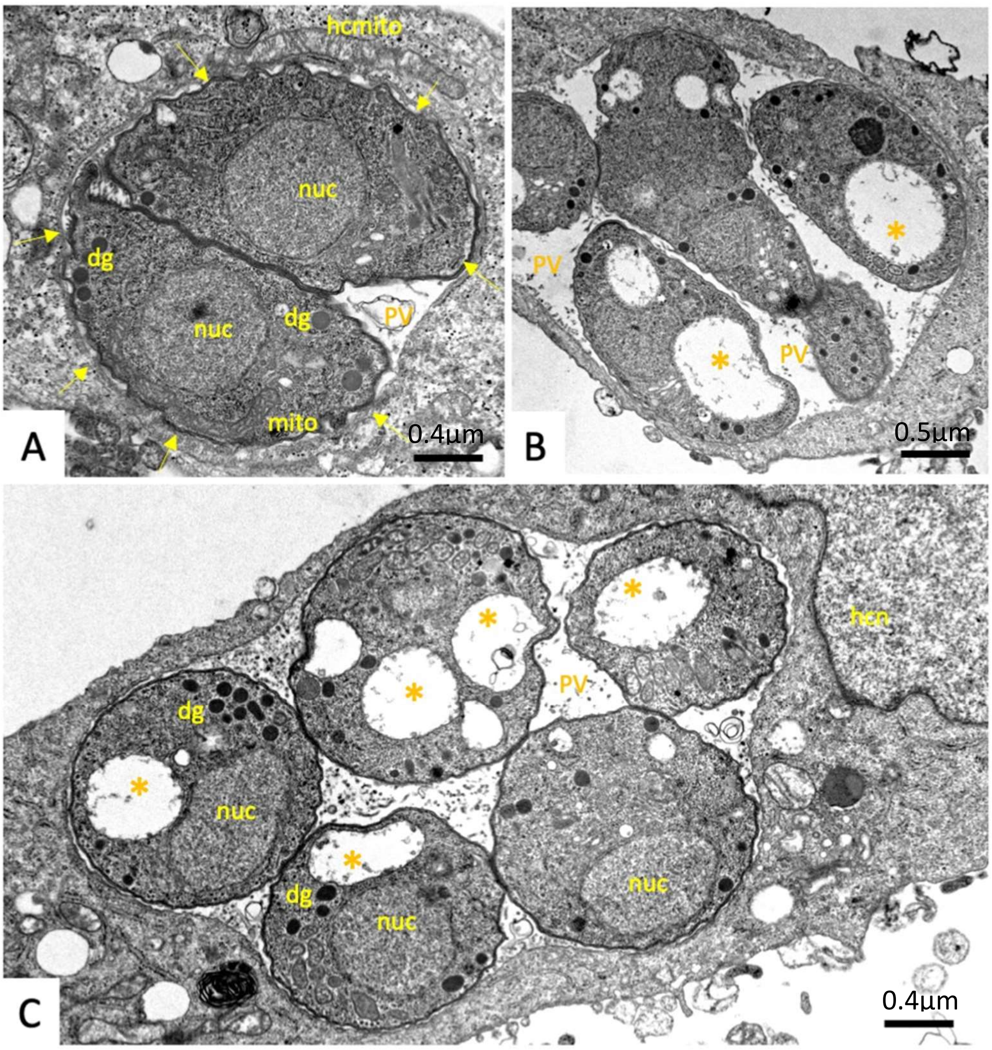

Figure S10. TEM of *T. gondii* ME49 tachyzoites treated with 500 nM of **17a** after 6 h (A), 24 h (B) and 48 h (C) after initiation of treatment. No effects are detected at 6 h, but clear vacuolization (marked with *) and a lack discernible mitochondria are seen at 24 to 48 h of treatment. Nuc = nucleus, hcmito = host cell mitochondrium; dg = dense granule; PV = parasitophorous vacuole; arrows point towards the parasitophorous vacuole membrane.

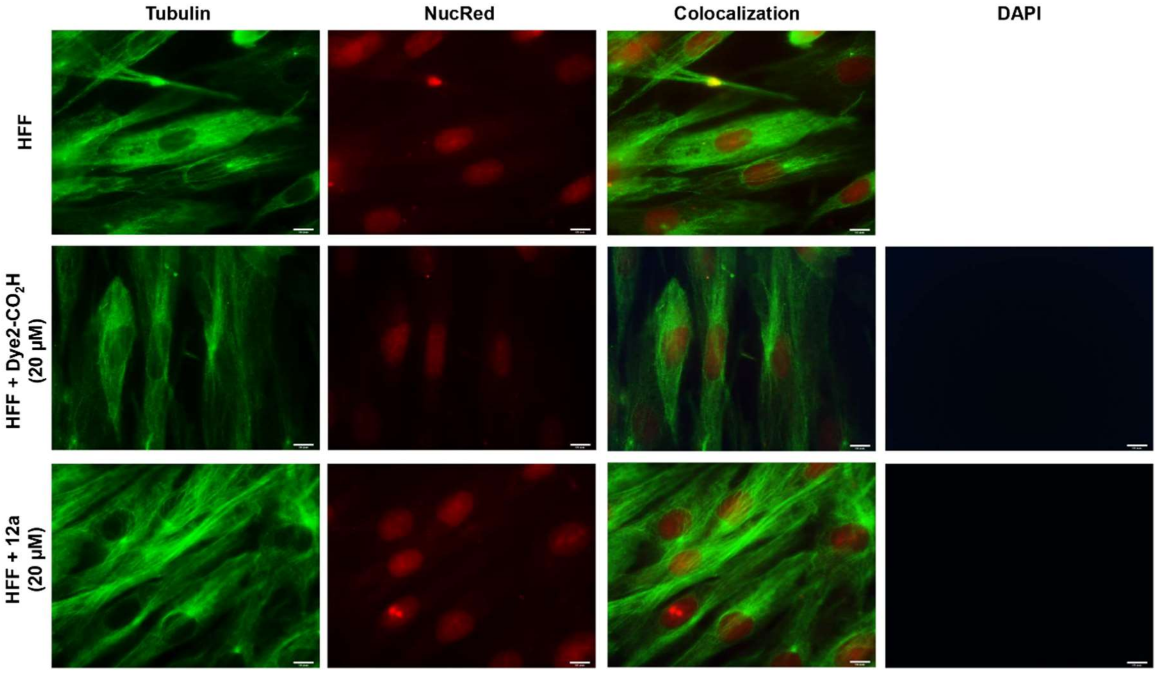

Figure S11. Fluorescence microscopy of HFF treated with 20 µM of **Dye2-CO2H**, **12a** or 0.5% DMSO, for 1 h at 37°C. **Dye2-CO2H**, and compound **12a** were visualized with the DAPI filter (excitation wavelength 461 nm). Cells were also stained with clone B-5-1-2 (monoclonal mouse anti-tubulin/anti-mouse fluorescein isothiocyanate, FITC) and NucRed reagent.

EXPERIMENTAL
Chemistry

General

$RuCl_3 \cdot 3H_2O$ was obtained from Fluorochem, and all other chemicals were purchased from Aldrich, Alfa Aesar, Acros, ABCR, or TCI Chemicals and used without further purification. Amberlyst® A21 free base was purchased from Aldrich and washed with MeOH before use. Reactions were performed under an inert atmosphere of N_2 using Schlenk techniques with dry solvents preserved on molecular sieves dried (Across Organics). The dimer [Ru(η^6-*p*-cymene)Cl]$_2$Cl$_2$ was prepared and purified according to literature procedures.[4] ^{1}H (400.13 MHz) and ^{13}C (100.62 MHz) NMR spectra were recorded on a Bruker Avance II 400 spectrometer at 298 K. ^{19}F (282.40 MHz) spectra were recorded on a Bruker Avance III 300 spectrometer at 298 K. The chemical shifts are reported in parts per million (ppm) and referenced to residual solvent peaks[5] ($CDCl_3$, ^{1}H δ 7.26, ^{13}C{^{1}H} δ 77.16 ppm; MeOD-d$_4$, ^{1}H δ 3.31, ^{13}C{^{1}H} δ 49.00 ppm, DMSO-d$_6$, ^{1}H δ 2.50, ^{13}C{^{1}H} δ 39.52 ppm) and coupling constants (J) are reported in hertz (Hz). High resolution electrospray ionization mass spectra (HRESI-MS) were carried out by the Mass Spectrometry and Protein Analyses Services at DCB and were obtained on a LTQ Orbitrap XL ESI (Thermo) operated in positive ion mode. Thermal elemental analyses were carried out by the Mass Spectrometry and Protein Analyses Services at DCB and were obtained on a Flash 2000 Organic Elemental Analyzer (Thermo Scientific). Reactions were monitored by TLC using Macherey-Nagel TLC silica gel coated aluminium sheets Alugram® Xtra SIL G/UV$_{254}$ and visualized with UV at 254 nm and 366 nm (coumarin functionalized compounds). Preparative TLC purifications were performed using Macherey-Nagel TLC silica gel glass pre-coated TCL plates SIL G-25 UV$_{254}$, and silica extracts were filtered on Macherey-Nagel disposable syringe filters Chromafil® Xtra PTFE-20-25 (pore size 0.20 µm). Compounds were purified by column flash chromatography on silica gel Sigma-Aldrich (60 Å, 230-400 mesh) using the elution systems indicated.

Abbreviations

DIPEA - *N,N*-Diisopropylethylamine
DMAP - 4-(Dimethylamino)-pyridine
DMF - Dimethylformamide
Dye1-CO$_2$H - 7-(diethylamino)-2-oxo-2*H*-chromene-3-carboxylic acid
Dye2-CO$_2$H - 11-oxo-2,3,6,7-tetrahydro-1*H*,5*H*,11*H*-pyrano[2,3-*f*]pyrido[3,2,1-*ij*]quinoline-10-carboxylic acid (coumarin 343)
EDCI - *N*-(3-Dimethylaminopropyl)-*N'*-ethylcarbodiimide hydrochloride
EtOAc – Ethyl acetate
Hex – n-Hexane
HOBt·H$_2$O - 1-Hydroxybenzotriazole hydrate
TEA – Triethylamine

For the description of the NMR spectra: *Ar* – arene, *Coum* – coumarin.

Synthesis and characterization

The dithiolato intermediates **1a** and **1b** were prepared and purified by adapting previously reported procedure.[6]

Synthesis of [(η^6-*p*-$MeC_6H_4Pr^i$)$_2$$Ru_2$($\mu_2$-S-$CH_2$-*p*-$C_6H_4Bu^t$)$Cl_2$] (1a)

To a solution of dimer ([Ru(η^6-*p*-$MeC_6H_4Pr^i$)Cl]$_2$$Cl_2$) (3.00 g, 4.899 mmol, 1 equiv) in EtOH (300 mL) at 0°C under inert atmosphere (N_2) was added dropwise a solution of 4-*tert*-butyl-benzene-methanethiol (1.767 g, 9.797 mmol, 2 equiv) in EtOH (10 mL). The reaction mixture was stirred at 0°C for further 4 h and then concentrated at 40°C under reduced pressure to small volume. The product was precipitated with Et_2O (100 mL), and then the solid was washed with Et_2O (3 x 50 mL) and dried to afford **1a** as an orange solid (4.03 g, 4.475 mmol, yield 91%).

***R*f** (CH_2Cl_2/CH_3OH 9.5:0.5) = 0.434; **^{1}H-NMR ($CDCl_3$) δ_H, ppm:** 7.49 (4H, d, 4xCH_2-(*Ar*)C-CH-C$\underline{H}$-C-C(CH_3)$_3$, $^3J_{H,H}$ = 8.1 Hz), 7.32 (4H, d, 4xCH_2-(*Ar*)C-C$\underline{H}$-CH-C-C(CH_3)$_3$, $^3J_{H,H}$ = 8.1 Hz), 4.93 (2H, m, 2xCH_3-(*Ar*)C-CH-C$\underline{H}$-C), 4.80 (4H, m, 2xCH_3-(*Ar*)C-CH-C$\underline{H}$-C, 2xCH_3-(*Ar*)C-C$\underline{H}$-CH-C), 4.11 (2H, d, 2xS-C$\underline{H}_2$-(*Ar*)C-CH-CH-C-C(CH_3)$_3$, $^2J_{H,H}$ = 11.3 Hz), 3.88 (2H, m, 2xCH_3-(*Ar*)C-C$\underline{H}$-CH-C), 3.32 (2H, d, 2xS-C$\underline{H}_2$-(*Ar*)C-CH-CH-C-C(CH_3)$_3$, $^2J_{H,H}$ = 11.3 Hz), 2.82 (2H, sept, 2x(*Ar*)C-CH-CH-C-C$\underline{H}$(CH_3)$_2$, $^3J_{H,H}$ = 6.8 Hz), 1.90 (6H, s, 2xC$\underline{H}_3$-(*Ar*)C-CH-CH-C), 1.33 (18H, s, 2xS-CH_2-(*Ar*)C-CH-CH-C-C(C$\underline{H}_3$)$_3$), 1.11-1.25 (12H, m, 2x(*Ar*)C-CH-CH-C-CH(C$\underline{H}_3$)$_2$); **^{13}C-NMR ($CDCl_3$) δ_C, ppm:** 150.1 (2C, 2xS-CH_2-(*Ar*)C-CH-CH-$\underline{C}$-C(CH_3)$_3$), 137.9 (2C, 2xS-CH_2-(*Ar*)$\underline{C}$-CH-CH-C-C(CH_3)$_3$), 130.3 (4C, 4xS-CH_2-(*Ar*)C-$\underline{C}$H-CH-C-C(CH_3)$_3$), 124.6 (4C, 4xS-CH_2-(*Ar*)C-CH-$\underline{C}$H-C-C(CH_3)$_3$), 105.9 (2C, 2xCH_3-(*Ar*)C-CH-CH-$\underline{C}$), 97.3 (2C, 2xCH_3-(*Ar*)$\underline{C}$-CH-CH-C), 85.4 (2C, 2xCH_3-(*Ar*)C-$\underline{C}$H-CH-C), 84.1 (2C, 2xCH_3-(*Ar*)C-CH-$\underline{C}$H-C), 83.0 (2C, 2xCH_3-(*Ar*)C-$\underline{C}$H-CH-C), 79.5 (2C, 2xCH_3-(*Ar*)C-CH-$\underline{C}$H-C), 36.6 (2C, 2xS-$\underline{C}H_2$-(*Ar*)C-CH-CH-C-C(CH_3)$_3$), 34.7 (2C, 2xS-CH_2-(*Ar*)C-CH-CH-C-$\underline{C}$(CH_3)$_3$), 31.6 (6C, 2xS-CH_2-(*Ar*)C-CH-CH-C-C($\underline{C}H_3$)$_3$), 30.0 (2C, 2x(*Ar*)CH-CH-C-$\underline{C}$H(CH_3)$_2$), 23.4 (2C, (*Ar*)CH-CH-C-CH($\underline{C}H_3$)$_2$), 21.6 (2C, (*Ar*)CH-CH-C-CH($\underline{C}H_3$)$_2$), 18.8 (2C, 2x$\underline{C}H_3$-(*Ar*)C-CH-CH); **HRMS (ESI(+)):** *m/z* calcd for $C_{42}H_{58}ClRu_2S_2^+$: 865.1750 [M-Cl]$^+$; found: 865.1779 (the isotopic pattern corresponds well to the calculated one).

Synthesis of [(η^6-*p*-$MeC_6H_4Pr^i$)$_2$$Ru_2$($\mu_2$-S-$CH_2C_6H_4$-*p*-$CF_3$)$Cl_2$]) (1b)

The dithiolato intermediate **1b** was prepared and purified by adapting previously reported procedure.[6] To a solution of dimer ([Ru(η^6-*p*-$MeC_6H_4Pr^i$)Cl]$_2$$Cl_2$) (0.600 g, 0.979 mmol, 1 equiv) in CH_2Cl_2 (35 mL) at 0°C under inert atmosphere (N_2) was added dropwise a solution of (4-(trifluoromethyl)phenyl)methanethiol (0.377 g, 1.960 mmol, 2 equiv) in CH_2Cl_2 (15 mL). The reaction mixture was concentrated under reduced pressure and the obtained product could be used for the next step without further purification; **1b** was isolated as an orange solid (quant. yield).

***R*f** (CH_2Cl_2/CH_3OH 9.5:0.5) = 0.455; **^{1}H-NMR ($CDCl_3$) δ_H, ppm:** 7.70 (4H, d, 4xS-CH_2-(*Ar*)C-C$\underline{H}$-CH-C-CF_3, $^3J_{H,H}$ = 8.1 Hz), 7.57 (4H, d, 4xS-CH_2-(*Ar*)C-CH-C$\underline{H}$-C-CF_3, $^3J_{H,H}$ = 8.1 Hz), 4.97 (2H, m, 2xCH_3-(*Ar*)C-CH-C$\underline{H}$-C), 4.90 (2H, m, 2xCH_3-(*Ar*)C-C$\underline{H}$-CH-C), 4.81 (2H, m, 2xCH_3-(*Ar*)C-CH-C$\underline{H}$-C), 4.28 (2H, d, 2xS-C$\underline{H}_2$-(*Ar*)C-CH-CH-C-CF_3, $^2J_{H,H}$ = 11.1 Hz), 3.80 (2H, m, 2xCH_3-(*Ar*)C-C$\underline{H}$-CH-C), 3.31 (2H, d, 2xS-C$\underline{H}_2$-(*Ar*)C-CH-CH-C-CF_3, $^2J_{H,H}$ = 11.1 Hz), 2.84 (2H, sept, 2x(*Ar*)C-CH-CH-C-C$\underline{H}$(CH_3)$_2$, $^3J_{H,H}$ = 6.9 Hz), 1.90 (6H, s, 2xC$\underline{H}_3$-(*Ar*)C-CH-CH-C), 1.17-1.25 (12H, m, 2x(*Ar*)C-CH-CH-C-CH(C$\underline{H}_3$)$_2$); **^{13}C-NMR ($CDCl_3$) δ_C, ppm:** 145.2 (2C, 2xS-CH_2-(*Ar*)$\underline{C}$-CH-CH-C-CF_3), 131.0 (4C, 4xS-CH_2-(*Ar*)C-$\underline{C}$H-CH-C-CF_3), 129.2 (2C, quat., 2xS-CH_2-(*Ar*)C-CH-CH-$\underline{C}$-

CF$_3$, $^2J_{C,F}$ = 32 Hz), 124.7 (4C, m, 4xS-CH$_2$-(*Ar*)C-CH-$\underline{C}$H-C-CF$_3$, $^3J_{C,F}$ = 4 Hz), 124.4 (2C, qvart, 2xS-CH$_2$-(*Ar*)C-CH-CH-C-$\underline{C}$F$_3$, $^1J_{C,F}$ = 272 Hz), 106.3 (2C, 2xCH$_3$-(*Ar*)C-CH-CH-$\underline{C}$), 97.4 (2C, 2xCH$_3$-(*Ar*)$\underline{C}$-CH-CH-C), 86.1 (2C, 2xCH$_3$-(*Ar*)C-CH-$\underline{C}$H-C), 83.8 (2C, 2xCH$_3$-(*Ar*)C-$\underline{C}$H-CH-C), 83.3 (2C, 2xCH$_3$-(*Ar*)C-$\underline{C}$H-CH-C), 79.3 (2C, 2xCH$_3$-(*Ar*)C-CH-$\underline{C}$H-C), 36.2 (2C, 2xS-$\underline{C}$H$_2$-(*Ar*)C-CH-CH-C-CF$_3$), 30.1 (2C, 2x(*Ar*)CH-CH-C-$\underline{C}$H(CH$_3$)$_2$), 23.6 (2C, (*Ar*)CH-CH-C-CH($\underline{C}$H$_3$)$_2$), 21.3 (2C, (*Ar*)CH-CH-C-CH($\underline{C}$H$_3$)$_2$), 18.9 (2C, 2x$\underline{C}$H$_3$-(*Ar*)C-CH-CH); **^{19}F-NMR (CDCl$_3$) δ_F, ppm:** -62.15 (6F, 2xC$\underline{F_3}$); **HRMS (ESI(+)):** *m/z* calcd for $C_{36}H_{40}ClF_6Ru_2S_2^+$: 889.0246 [M-Cl]$^+$; found: 889.0263 (the isotopic pattern corresponds well to the calculated one).

The mixed trithiolato ruthenium(II)-arene complexes **2a/b**, **3a/b** and **4a/b** were prepared and purified by adapting a formerly published protocol.[7]

Synthesis of [(η^6-*p*-MeC$_6$H$_4$^{i}Pr)$_2$Ru$_2$(SCH$_2$-*p*-C$_6$H$_4$*t*Bu)$_2$(SC$_6$H$_4$-*p*-OH)]Cl (2a)

To a solution of **1a** (1.290 g, 1.433 mmol, 1 equiv) in refluxing EtOH (150 mL) was added dropwise a solution of 4-hydroxy-benzenethiol (0.542 g, 4.300 mmol, 3 equiv) in EtOH (20 mL). The reaction mixture was further heated at reflux under inert atmosphere (N$_2$) for 24 h. The reaction evolution was verified by TLC and then the reaction mixture was concentrated to dryness under reduced pressure. Purification by column chromatography using CH$_2$Cl$_2$/CH$_3$OH mixture afforded **2a** as an orange solid (1.353 g, 1.366 mmol, yield 95%).

***R*f** (CH$_2$Cl$_2$/CH$_3$OH 10:1) = 0.467; **^{1}H-NMR (CDCl$_3$) δ_H, ppm:** 10.36 (1H, s, OH), 7.38-7.48 (10H, m, 2xS-(*Ar*)C-C$\underline{H}$-CH-C-OH, 4xCH$_2$-(*Ar*)C-C$\underline{H}$-CH-C-C(CH$_3$)$_3$, 4xCH$_2$-(*Ar*)C-CH-C$\underline{H}$-C-C(CH$_3$)$_3$), 7.24 (2H, m, 2xS-(*Ar*)C-CH-C$\underline{H}$-C-OH, $^3J_{H,H}$ = 8.7 Hz), 4.98 (2H, d, 2xCH$_3$-(*Ar*)C-CH-C$\underline{H}$-C, $^3J_{H,H}$ = 5.8 Hz), 4.88 (2H, d, 2xCH$_3$-(*Ar*)C-CH-C$\underline{H}$-C, $^3J_{H,H}$ = 5.8 Hz), 4.70 (2H, d, 2xCH$_3$-(*Ar*)C-C$\underline{H}$-CH-C, $^3J_{H,H}$ = 5.7 Hz), 4.58 (2H, d, 2xCH$_3$-(*Ar*)C-C$\underline{H}$-CH-C, $^3J_{H,H}$ = 5.8 Hz), 3.54 (2H, s, S-C$\underline{H_2}$-(*Ar*)C-CH-CH-C-C(CH$_3$)$_3$), 3.36 (2H, s, S-C$\underline{H_2}$-(*Ar*)C-CH-CH-C-C(CH$_3$)$_3$), 1.97 (2H, sept, 2x(*Ar*)C-CH-CH-C-C$\underline{H}$(CH$_3$)$_2$, $^3J_{H,H}$ = 6.9 Hz), 1.69 (6H, s, 2xC$\underline{H_3}$-(*Ar*)C-CH-CH-C), 1.36 (9H, s, S-CH$_2$-(*Ar*)C-CH-CH-C-C(C$\underline{H_3}$)$_3$), 1.34 (9H, s, S-CH$_2$-(*Ar*)C-CH-CH-C-C(C$\underline{H_3}$)$_3$), 0.97 (6H, d, (*Ar*)C-CH-CH-C-CH(C$\underline{H_3}$)$_2$, $^3J_{H,H}$ = 6.9 Hz), 0.93 (6H, d, (*Ar*)C-CH-CH-C-CH(C$\underline{H_3}$)$_2$, $^3J_{H,H}$ = 6.9 Hz); **^{13}C-NMR (CDCl$_3$) δ_C, ppm:** 160.1 (1C, S-(*Ar*)C-CH-CH-$\underline{C}$-OH), 151.9, 151.8 (2C, 2xS-CH$_2$-(*Ar*)C-CH-CH-$\underline{C}$-C(CH$_3$)$_3$), 137.0, 136.7 (2C, 2xS-CH$_2$-(*Ar*)$\underline{C}$-CH-CH-C-C(CH$_3$)$_3$), 133.4 (2C, 2xS-(*Ar*)C-$\underline{C}$H-CH-C-OH), 129.3, 129.1 (4C, 4xS-CH$_2$-(*Ar*)C-$\underline{C}$H-CH-C-C(CH$_3$)$_3$), 125.7, 125.6 (4C, 4xS-CH$_2$-(*Ar*)C-CH-$\underline{C}$H-C-C(CH$_3$)$_3$), 124.1 (1C, S-(*Ar*)$\underline{C}$-CH-CH-C-OH), 117.3 (2C, 2xS-(*Ar*)C-CH-$\underline{C}$H-C-OH), 107.5 (2C, 2xCH$_3$-(*Ar*)C-CH-CH-$\underline{C}$), 100.2 (2C, 2xCH$_3$-(*Ar*)$\underline{C}$-CH-CH-C), 84.1 (2C, 2xCH$_3$-(*Ar*)C-CH-$\underline{C}$H-C),83.6 (2C, 2xCH$_3$-(*Ar*)C-$\underline{C}$H-CH-C), 83.5 (2C, 2xCH$_3$-(*Ar*)C-CH-$\underline{C}$H-C), 82.2 (2C, 2xCH$_3$-(*Ar*)C-$\underline{C}$H-CH-C), 39.9 (1C, S-$\underline{C}$H$_2$-(*Ar*)C-CH-CH-C-C(CH$_3$)$_3$), 39.2 (1C, S-$\underline{C}$H$_2$-(*Ar*)C-CH-CH-C-C(CH$_3$)$_3$), 34.91 (1C, S-CH$_2$-(*Ar*)C-CH-CH-C-$\underline{C}$(CH$_3$)$_3$), 34.88 (1C, S-CH$_2$-(*Ar*)C-CH-CH-C-$\underline{C}$(CH$_3$)$_3$), 31.55 (3C, S-CH$_2$-(*Ar*)C-CH-CH-C-C($\underline{C}$H$_3$)$_3$), 31.54 (3C, S-CH$_2$-(*Ar*)C-CH-CH-C-C($\underline{C}$H$_3$)$_3$), 31.0 (2C, 2x(*Ar*)CH-CH-C-$\underline{C}$H(CH$_3$)$_2$), 23.2 (2C, (*Ar*)CH-CH-C-CH($\underline{C}$H$_3$)$_2$), 22.9 (2C, (*Ar*)CH-CH-C-CH($\underline{C}$H$_3$)$_2$), 18.1 (2C, 2x$\underline{C}$H$_3$-(*Ar*)C-CH-CH); **HRMS (ESI(+)):** *m/z* calcd for $C_{48}H_{63}ORu_2S_3^+$: 955.2123 [M-Cl]$^+$; found: 955.2102 (the isotopic pattern corresponds well to the calculated one); **elemental analysis** calcd (%) for $C_{48}H_{63}ClORu_2S_3\cdot 2CH_3OH$: C 56.98, H 6.79; found C 56.91, H 6.78.

Synthesis of [(η^6-*p*-$MeC_6H_4Pr^i$)$_2Ru_2$(μ_2-$SCH_2C_6H_4$-*p*-CF_3)$_2$(μ_2-SC_6H_4-*p*-OH)]Cl (2b)

To a solution of **1b** (0.300 g, 0.325 mmol, 1 equiv) in refluxing EtOH (100 mL) was added dropwise a solution of 4-hydroxy-benzenethiol (0.123 g, 0.975 mmol, 3 equiv) in EtOH (20 mL). The reaction mixture was further heated at reflux under inert atmosphere (N_2) for 24 h. The reaction evolution was verified by TLC and then the reaction mixture was concentrated to dryness under reduced pressure. Purification by column chromatography using CH_2Cl_2/CH_3OH mixture as eluent afforded **2b** as an orange solid (0.252 g, 0.248 mmol, yield 76%).

***R*f** (CH_2Cl_2/CH_3OH 9.5:0.5) = 0.262; **^{1}H-NMR ($CDCl_3$) δ_H, ppm:** 7.66-7.76 (8H, m, 4xS-CH_2-(*Ar*)C-C$\underline{H}$-CH-C-CF_3, 4xS-CH_2-(*Ar*)C-CH-C$\underline{H}$-C-CF_3, $^3J_{H,H}$ = 8.4 Hz), 7.52 (2H, d, 2xS-(*Ar*)C-C$\underline{H}$-CH-C-OH, $^3J_{H,H}$ = 8.5 Hz), 7.21 (2H, d, 2xS-(*Ar*)C-CH-C$\underline{H}$-C-OH, $^3J_{H,H}$ = 8.5 Hz), 5.12 (2H, d, 2xCH_3-(*Ar*)C-CH-C$\underline{H}$-C, $^3J_{H,H}$ = 5.7 Hz), 5.06 (2H, d, 2xCH_3-(*Ar*)C-CH-C$\underline{H}$-C, $^3J_{H,H}$ = 5.8 Hz), 4.85 (2H, d, 2xCH_3-(*Ar*)C-C$\underline{H}$-CH-C, $^3J_{H,H}$ = 5.7 Hz), 4.77 (2H, d, 2xCH_3-(*Ar*)C-C$\underline{H}$-CH-C, $^3J_{H,H}$ = 5.8 Hz), 3.71 (2H, s, S-C$\underline{H_2}$-(*Ar*)C-CH-CH-C-CF_3), 3.56 (2H, s, S-C$\underline{H_2}$-(*Ar*)C-CH-CH-C-CF_3), 1.94 (2H, sept, 2x(*Ar*)C-CH-CH-C-C$\underline{H}$(CH_3)$_2$, $^3J_{H,H}$ = 6.8 Hz), 1.69 (6H, s, 2xC$\underline{H_3}$-(*Ar*)C-CH-CH-C), 0.99 (6H, d, (*Ar*)C-CH-CH-C-CH(C$\underline{H_3}$)$_2$, $^3J_{H,H}$ = 6.8 Hz), 0.91 (6H, d, (*Ar*)C-CH-CH-C-CH(C$\underline{H_3}$)$_2$, $^3J_{H,H}$ = 6.8 Hz); **^{13}C-NMR ($CDCl_3$) δ_C, ppm:** 159.9 (1C, S-(*Ar*)C-CH-CH-$\underline{C}$-OH), 144.3, 144.2 (2C, 2xS-CH_2-(*Ar*)$\underline{C}$-CH-CH-C-CF_3), 133.7 (2C, 2xS-(*Ar*)C-$\underline{C}$H-CH-C-OH), 130.7 (1C, m, S-CH_2-(*Ar*)C-CH-CH-$\underline{C}$-CF_3, $^2J_{C,F}$ = 32 Hz), 130.4 (1C, m, S-CH_2-(*Ar*)C-CH-CH-$\underline{C}$-CF_3, $^2J_{C,F}$ = 32 Hz), 130.1 (2C, 2xS-CH_2-(*Ar*)C-$\underline{C}$H-CH-C-CF_3), 129.8 (2C, 2xS-CH_2-(*Ar*)C-$\underline{C}$H-CH-C-CF_3), 125.8 (2C, 2xS-CH_2-(*Ar*)C-CH-$\underline{C}$H-C-CF_3, $^3J_{C,F}$ = 4 Hz), 125.6 (2C, 2xS-CH_2-(*Ar*)C-CH-$\underline{C}$H-C-CF_3, $^3J_{C,F}$ = 4 Hz), 124.2 (1C, S-(*Ar*)$\underline{C}$-CH-CH-C-OH), 124.1 (2C, qvart, 2xS-CH_2-(*Ar*)C-CH-CH-C-$\underline{C}F_3$, $^1J_{C,F}$ = 272 Hz), 117.2 (2C, 2xS-(*Ar*)C-CH-$\underline{C}$H-C-OH), 107.8 (2C, 2xCH_3-(*Ar*)C-CH-CH-$\underline{C}$), 100.3 (2C, 2xCH_3-(*Ar*)$\underline{C}$-CH-CH-C), 84.4 (2C, 2xCH_3-(*Ar*)C-CH-$\underline{C}$H-C), 84.0 (2C, 2xCH_3-(*Ar*)C-$\underline{C}$H-CH-C), 83.5 (2C, 2xCH_3-(*Ar*)C-CH-$\underline{C}$H-C), 82.2 (2C, 2xCH_3-(*Ar*)C-$\underline{C}$H-CH-C), 39.9 (1C, S-$\underline{C}H_2$-(*Ar*)C-CH-CH-C-CF_3), 39.5 (1C, S-$\underline{C}H_2$-(*Ar*)C-CH-CH-C-CF_3), 31.0 (2C, 2x(*Ar*)CH-CH-C-$\underline{C}$H(CH_3)$_2$), 23.2 (2C, (*Ar*)CH-CH-C-CH($\underline{C}H_3$)$_2$), 22.7 (2C, (*Ar*)CH-CH-C-CH($\underline{C}H_3$)$_2$), 18.1 (2C, 2x$\underline{C}H_3$-(*Ar*)C-CH-CH); **^{19}F-NMR ($CDCl_3$) δ_F, ppm:** -62.30, -62.34 (6F, 2xC$\underline{F_3}$); **HRMS (ESI(+)):** *m/z* calcd for $C_{42}H_{45}F_6ORu_2S_3^+$: 979.0618 [M-Cl]$^+$; found: 979.0640 (the isotopic pattern corresponds well to the calculated one); **elemental analysis** calcd (%) for $C_{42}H_{45}ClF_6ORu_2S_3$·2.5$CH_3OH$: C 48.87, H 5.07; found C 48.99, H 5.08.

Synthesis of [(η^6-*p*-$MeC_6H_4Pr^i$)$_2Ru_2$(μ_2-$SCH_2C_6H_4$-p-Bu^t)$_2$(μ_2-SC_6H_4-*p*-NH_2)]Cl (3a)

To as solution of **1a** (2.00 g, 2.221 mmol, 1 equiv) in refluxing EtOH (140 mL) was added dropwise a solution of 4-amino-benzenethiol (0.809 g, 6.663 mmol, 3 equiv) in EtOH (10 mL). The reaction mixture was further heated at reflux under inert atmosphere (N_2) for 24 h. The reaction evolution was verified by TLC and then the reaction mixture was concentrated to dryness under reduced pressure. Purification by column chromatography using CH_2Cl_2/CH_3OH 10:1 mixture afforded **3a** as an orange solid (2.10 g, 2.126 mmol, yield 96%).

***R*f** (CH_2Cl_2/CH_3OH 10:1) = 0.518; **^{1}H-NMR ($CDCl_3$) δ_H, ppm:** 7.39-7.52 (10H, m, 2xS-(*Ar*)C-C$\underline{H}$-CH-C-NH_2, 4xCH_2-(*Ar*)C-C$\underline{H}$-CH-C-C(CH_3)$_3$, 4xCH_2-(*Ar*)C-CH-C$\underline{H}$-C-C(CH_3)$_3$), 6.75 (2H, m, 2xS-(*Ar*)C-CH-C$\underline{H}$-C-NH_2, $^3J_{H,H}$ = 8.5 Hz), 5.01 (2H, d, 2xCH_3-(*Ar*)C-CH-C$\underline{H}$-C, $^3J_{H,H}$ = 5.6 Hz), 4.90 (2H, d, 2xCH_3-(*Ar*)C-CH-C$\underline{H}$-C, $^3J_{H,H}$ = 5.7 Hz), 4.74 (2H, d, 2xCH_3-(*Ar*)C-C$\underline{H}$-CH-C, $^3J_{H,H}$ = 5.7 Hz), 4.58 (2H, d, 2xCH_3-(*Ar*)C-C$\underline{H}$-CH-C, $^3J_{H,H}$ = 5.8 Hz), 3.54 (2H, s, S-C$\underline{H_2}$-(*Ar*)C-CH-

CH-C-C(CH$_3$)$_3$), 3.37 (2H, s, S-C*H$_2$*-(*Ar*)C-CH-CH-C-C(CH$_3$)$_3$), 1.97 (2H, sept, 2x(*Ar*)C-CH-CH-C-C*H*(CH$_3$)$_2$, $^3J_{H,H}$ = 6.9 Hz), 1.71 (6H, s, 2xC*H$_3$*-(*Ar*)C-CH-CH-C), 1.36 (9H, s, S-CH$_2$-(*Ar*)C-CH-CH-C-C(C*H$_3$*)$_3$), 1.34 (9H, s, S-CH$_2$-(*Ar*)C-CH-CH-C-C(C*H$_3$*)$_3$), 0.98 (6H, d, (*Ar*)C-CH-CH-C-CH(C*H$_3$*)$_2$, $^3J_{H,H}$ = 6.9 Hz), 0.93 (6H, d, (*Ar*)C-CH-CH-C-CH(C*H$_3$*)$_2$, $^3J_{H,H}$ = 6.9 Hz); **^{13}C-NMR (CDCl$_3$) δ$_C$, ppm:** 151.85, 151.77 (2C, 2xS-CH$_2$-(*Ar*)C-CH-CH-*C*-C(CH$_3$)$_3$), 148.8 (1C, S-(*Ar*)C-CH-CH-*C*-NH$_2$), 137.0, 136.8 (2C, 2xS-CH$_2$-(*Ar*)*C*-CH-CH-C-C(CH$_3$)$_3$), 133.6 (2C, 2xS-(*Ar*)C-*C*H-CH-C-NH$_2$), 129.3, 129.1 (4C, 4xS-CH$_2$-(*Ar*)C-*C*H-CH-C-C(CH$_3$)$_3$), 125.7, 125.5 (4C, 4xS-CH$_2$-(*Ar*)C-CH-*C*H-C-C(CH$_3$)$_3$), 122.9 (1C, S-(*Ar*)*C*-CH-CH-C-NH$_2$), 115.6 (2C, 2xS-(*Ar*)C-CH-*C*H-C-NH$_2$), 107.3 (2C, 2xCH$_3$-(*Ar*)C-CH-CH-*C*), 100.2 (2C, 2xCH$_3$-(*Ar*)*C*-CH-CH-C), 84.1 (2C, 2xCH$_3$-(*Ar*)C-CH-*C*H-C), 83.64 (2C, 2xCH$_3$-(*Ar*)C-*C*H-CH-C), 83.56 (2C, 2xCH$_3$-(*Ar*)C-CH-*C*H-C), 82.2 (2C, 2xCH$_3$-(*Ar*)C-*C*H-CH-C), 39.9 (1C, S-*C*H$_2$-(*Ar*)C-CH-CH-C-C(CH$_3$)$_3$), 39.2 (1C, S-*C*H$_2$-(*Ar*)C-CH-CH-C-C(CH$_3$)$_3$), 34.91 (1C, S-CH$_2$-(*Ar*)C-CH-CH-C-*C*(CH$_3$)$_3$), 34.88 (1C, S-CH$_2$-(*Ar*)C-CH-CH-C-*C*(CH$_3$)$_3$), 31.6 (6C, 2xS-CH$_2$-(*Ar*)C-CH-CH-C-C(*C*H$_3$)$_3$), 31.0 (2C, 2x(*Ar*)CH-CH-C-*C*H(CH$_3$)$_2$), 23.2 (2C, (*Ar*)CH-CH-C-CH(*C*H$_3$)$_2$), 22.9 (2C, (*Ar*)CH-CH-C-CH(*C*H$_3$)$_2$), 18.2 (2C, 2x*C*H$_3$-(*Ar*)C-CH-CH); **HRMS (ESI(+)):** *m/z* calcd for $C_{48}H_{64}NRu_2S_3^+$: 954.2282 [M-Cl]$^+$; found: 954.2291 (the isotopic pattern corresponds well to the calculated one); **elemental analysis** calcd (%) for $C_{48}H_{64}ClNRu_2S_3$·3.5CH$_3$OH: C 56.18, H 7.14, N 1.27; found C 56.17, H 7.33, N 1.19.

Synthesis of [(η^6-*p*-MeC$_6$H$_4$Pr^i)$_2$Ru$_2$(μ$_2$-SCH$_2$C$_6$H$_4$-*p*-CF$_3$)$_2$(μ$_2$-SC$_6$H$_4$-*p*-NH$_2$)]Cl (3b)

To a solution of **1b** (0.275 g, 0.298 mmol, 1 equiv) in refluxing EtOH (100 mL) was added dropwise a solution of 4-amino-benzenethiol (0.112 g, 0.894 mmol, 3 equiv) in EtOH (20 mL). The mixture was further heated at reflux under inert atmosphere (N$_2$) for 24 h. The reaction evolution was verified by TLC and then the reaction mixture was concentrated to dryness under reduced pressure. Purification by column chromatography using CH$_2$Cl$_2$/CH$_3$OH mixture afforded **3b** as an orange solid (0.258 g, 0.255 mmol, yield 85%).

***R*f** (CH$_2$Cl$_2$/CH$_3$OH 9.5:0.5) = 0.215; **^{1}H-NMR (CDCl$_3$) δ$_H$, ppm:** 7.66-7.76 (8H, m, 4xCH$_2$-(*Ar*)C-C*H*-CH-C-CF$_3$, 4xCH$_2$-(*Ar*)C-CH-C*H*-C-CF$_3$, $^3J_{H,H}$ = 8.5 Hz), 7.46 (2H, d, 2xS-(*Ar*)C-C*H*-CH-C-NH$_2$, $^3J_{H,H}$ = 8.4 Hz), 6.76 (2H, d, 2xS-(*Ar*)C-CH-C*H*-C-NH$_2$, $^3J_{H,H}$ = 8.4 Hz), 5.15 (2H, d, 2xCH$_3$-(*Ar*)C-CH-C*H*-C, $^3J_{H,H}$ = 5.7 Hz), 5.09 (2H, d, 2xCH$_3$-(*Ar*)C-CH-C*H*-C, $^3J_{H,H}$ = 5.8 Hz), 4.88 (2H, d, 2xCH$_3$-(*Ar*)C-C*H*-CH-C, $^3J_{H,H}$ = 5.7 Hz), 4.77 (2H, d, 2xCH$_3$-(*Ar*)C-C*H*-CH-C, $^3J_{H,H}$ = 5.8 Hz), 3.71 (2H, s, S-C*H$_2$*-(*Ar*)C-CH-CH-C-CF$_3$), 3.59 (2H, s, S-C*H$_2$*-(*Ar*)C-CH-CH-C-CF$_3$), 1.93 (2H, sept, 2x(*Ar*)C-CH-CH-C-C*H*(CH$_3$)$_2$, $^3J_{H,H}$ = 6.8 Hz), 1.72 (6H, s, 2xC*H$_3$*-(*Ar*)C-CH-CH-C), 1.00 (6H, d, (*Ar*)C-CH-CH-C-CH(C*H$_3$*)$_2$, $^3J_{H,H}$ = 6.8 Hz), 0.93 (6H, d, (*Ar*)C-CH-CH-C-CH(C*H$_3$*)$_2$, $^3J_{H,H}$ = 6.8 Hz); **^{13}C-NMR (CDCl$_3$) δ$_C$, ppm:** 148.3 (1C, S-(*Ar*)C-CH-CH-*C*-NH$_2$), 144.3 (2C, 2xS-CH$_2$-(*Ar*)*C*-CH-CH-C-CF$_3$), 133.8 (2C, 2xS-(*Ar*)C-*C*H-CH-C-NH$_2$), 130.4 (1C, m, S-CH$_2$-(*Ar*)C-CH-CH-*C*-CF$_3$, $^2J_{C,F}$ = 32 Hz), 130.1 (1C, m, S-CH$_2$-(*Ar*)C-CH-CH-*C*-CF$_3$, $^2J_{C,F}$ = 32 Hz), 130.1 (2C, 2xS-CH$_2$-(*Ar*)C-*C*H-CH-C-CF$_3$), 129.9 (2C, 2xS-CH$_2$-(*Ar*)C-*C*H-CH-C-CF$_3$), 125.8 (2C, 2xS-CH$_2$-(*Ar*)C-CH-*C*H-C-CF$_3$, $^3J_{C,F}$ = 4 Hz), 125.6 (2C, 2xS-CH$_2$-(*Ar*)C-CH-*C*H-C-CF$_3$, $^3J_{C,F}$ = 4 Hz), 124.2 (1C, qvart, S-CH$_2$-(*Ar*)C-CH-CH-C-*C*F$_3$, $^1J_{C,F}$ = 272 Hz), 124.1 (1C, qvart, S-CH$_2$-(*Ar*)C-CH-CH-C-*C*F$_3$, $^1J_{C,F}$ = 272 Hz), 123.2 (1C, S-(*Ar*)*C*-CH-CH-C-NH$_2$), 115.8 (2C, 2xS-(*Ar*)C-CH-*C*H-C-NH$_2$), 107.8 (2C, 2xCH$_3$-(*Ar*)C-CH-CH-*C*), 100.3 (2C, 2xCH$_3$-(*Ar*)*C*-CH-CH-C), 84.4 (2C, 2xCH$_3$-(*Ar*)C-CH-*C*H-C), 84.0 (2C, 2xCH$_3$-(*Ar*)C-*C*H-CH-C), 83.6 (2C, 2xCH$_3$-(*Ar*)C-CH-*C*H-C), 82.2 (2C, 2xCH$_3$-(*Ar*)C-*C*H-CH-C), 40.0 (1C, S-*C*H$_2$-(*Ar*)C-CH-CH-C-CF$_3$), 39.5 (1C, S-*C*H$_2$-(*Ar*)C-CH-CH-C-CF$_3$), 31.0 (2C, 2x(*Ar*)CH-CH-C-*C*H(CH$_3$)$_2$), 23.2 (2C, (*Ar*)CH-CH-C-CH(*C*H$_3$)$_2$), 22.7 (2C,

(*Ar*)CH-CH-C-CH(*C*H3)2), 18.1 (2C, 2x*C*H3-(*Ar*)C-CH-CH); **^{19}F-NMR (CDCl3) δ_F, ppm:** -62.32, -62.37 (6F, 2xC*F3*); **HRMS (ESI(+)):** *m/z* calcd for $C_{42}H_{46}F_6NRu_2S_3^+$: 978.0778 [M-Cl]$^+$; found: 978.0803 (the isotopic pattern corresponds well to the calculated one); **elemental analysis** calcd (%) for $C_{42}H_{46}ClF_6NRu_2S_3$·2.5CH3OH: C 48.91, H 5.17, N 1.28; found C 48.88, H 5.19, N 2.71.

Synthesis of [(η^6-*p*-MeC6H4Pr^i)2Ru2(μ_2-SCH2C6H4-*p*-Bu^t)2(μ_2-SC6H4-*p*-CH2CO2H)]Cl (4a)
To a solution of **1a** (2.500 g, 2.778 mmol, 1 equiv) in refluxing CH_2Cl_2 (200 mL) was added dropwise under inert atmosphere (N_2) a solution of 4-mercaptophenylacetic acid (0.934 g, 5.555 mmol, 2 equiv) in acetone (20 mL). The reaction mixture was further heated at reflux under inert atmosphere (N_2) for 24 h. The reaction evolution was verified by TLC and then the reaction mixture was concentrated to dryness under reduced pressure. Purification by column chromatography using CH_2Cl_2/CH_3OH mixture as eluent afforded **4a** as an orange solid (2.693 g, 2.610 mmol, yield 94%). ***R*f** (CH_2Cl_2/CH_3OH 10:1) = 0.364; **^{1}H-NMR (CDCl3) δ_H, ppm:** 7.62 (2H, d, 2xS-(*Ar*)C-CH-C*H*-C-CH2-CO2H, $^3J_{H,H}$ = 7.8 Hz), 7.38-7.52 (10H, m, 2xS-(*Ar*)C-C*H*-CH-C-CH2-CO2H, 4xCH2-(*Ar*)C-C*H*-CH-C-C(CH3)3, 4xCH2-(*Ar*)C-CH-C*H*-C-C(CH3)3), 5.02 (2H, d, 2xCH3-(*Ar*)C-CH-C*H*-C, $^3J_{H,H}$ = 5.5 Hz), 4.89 (2H, d, 2xCH3-(*Ar*)C-CH-C*H*-C, $^3J_{H,H}$ = 5.6 Hz), 4.80 (2H, d, 2xCH3-(*Ar*)C-C*H*-CH-C, $^3J_{H,H}$ = 4.8 Hz), 4.58 (2H, d, 2xCH3-(*Ar*)C-C*H*-CH-C, $^3J_{H,H}$ = 5.6 Hz), 3.93 (2H, s, S-(*Ar*)C-CH-CH-C-C*H2*-CO2H), 3.57 (2H, s, S-C*H2*-(*Ar*)C-CH-CH-C-C(CH3)3), 3.36 (2H, s, S-C*H2*-(*Ar*)C-CH-CH-C-C(CH3)3), 1.90 (2H, sept, 2x(*Ar*)C-CH-CH-C-C*H*(CH3)2, $^3J_{H,H}$ = 6.8 Hz), 1.69 (6H, s, 2xC*H3*-(*Ar*)C-CH-CH-C), 1.35 (9H, s, S-CH2-(*Ar*)C-CH-CH-C-C(C*H3*)3), 1.32 (9H, s, S-CH2-(*Ar*)C-CH-CH-C-C(C*H3*)3), 0.94 (6H, d, (*Ar*)C-CH-CH-C-CH(C*H3*)2, $^3J_{H,H}$ = 6.8 Hz), 0.89 (6H, d, (*Ar*)C-CH-CH-C-CH(C*H3*)2, $^3J_{H,H}$ = 6.8 Hz); **^{13}C-NMR (CDCl3) δ_C, ppm:** 173.9 (1C, S-(*Ar*)C-CH-CH-C-CH2-*C*O2H), 151.91, 151.86 (2C, 2xS-CH2-(*Ar*)C-CH-CH-*C*-C(CH3)3), 137.1 (1C, S-(*Ar*)C-CH-CH-*C*-CH2-CO2H), 136.8, 136.6 (2C, 2xS-CH2-(*Ar*)*C*-CH-CH-C-C(CH3)3), 135.1 (1C, S-(*Ar*)*C*-CH-CH-C-CH2-CO2H), 132.3 (2C, 2xS-(*Ar*)C-CH-*C*H-C-CH2-CO2H), 130.8 (2C, 2xS-(*Ar*)C-*C*H-CH-C-CH2-CO2H), 129.4, 129.1 (4C, 4xS-CH2-(*Ar*)C-*C*H-CH-C-C(CH3)3), 125.7, 125.6 (4C, 4xS-CH2-(*Ar*)C-CH-*C*H-C-C(CH3)3), 107.4 (2C, 2xCH3-(*Ar*)C-CH-CH-*C*), 100.7 (2C, 2xCH3-(*Ar*)*C*-CH-CH-C), 83.92 (2C, 2xCH3-(*Ar*)C-CH-*C*H-C), 83.86 (2C, 2xCH3-(*Ar*)C-CH-*C*H-C), 83.6 (2C, 2xCH3-(*Ar*)C-*C*H-CH-C), 82.4 (2C, 2xCH3-(*Ar*)C-*C*H-CH-C), 42.3 (1C, S-(*Ar*)C-CH-CH-C-*C*H2-CO2H), 40.0 (1C, S-*C*H2-(*Ar*)C-CH-CH-C-C(CH3)3), 39.4 (1C, S-*C*H2-(*Ar*)C-CH-CH-C-C(CH3)3), 34.91 (1C, S-CH2-(*Ar*)C-CH-CH-C-*C*(CH3)3), 34.88 (1C, S-CH2-(*Ar*)C-CH-CH-C-*C*(CH3)3), 31.6 (6C, 2xS-CH2-(*Ar*)C-CH-CH-C-C(*C*H3)3), 31.0 (2C, 2x(*Ar*)CH-CH-C-*C*H(CH3)2), 23.2 (2C, (*Ar*)CH-CH-C-CH(*C*H3)2), 22.8 (2C, (*Ar*)CH-CH-C-CH(*C*H3)2), 18.2 (2C, 2x*C*H3-(*Ar*)C-CH-CH); **HRMS (ESI(+)):** *m/z* calcd for $C_{50}H_{65}O_2Ru_2S_3^+$: 997.2228 [M-Cl]$^+$; found: 997.2227 (the isotopic pattern corresponds well to the calculated one); **elemental analysis** calcd (%) for $C_{50}H_{65}ClO_2Ru_2S_3$·2.5CH2Cl2·CH3OH: C 50.35, H 5.84; found C 50.31; H 5.86.

Synthesis of [(η^6-*p*-MeC6H4Pr^i)2Ru2(μ_2-SCH2C6H4-*p*-CF3)2(μ_2-SC6H4-*p*-CH2CO2H)]Cl (4b)
To a solution of **1b** (0.330 g, 0.357 mmol, 1 equiv) in CH_2Cl_2 (130 mL) was added dropwise under inert atmosphere (N_2) a solution of 4-mercaptophenylacetic acid (0.120 g, 0.714 mmol, 2 equiv) in acetone (20 mL). The reaction mixture was further heated at reflux under inert atmosphere (N_2) for 24 h. The reaction evolution was verified by TLC and then the reaction mixture was concentrated to dryness under reduced pressure. Purification by column chromatography using CH_2Cl_2/CH_3OH mixture afforded **4b** as an orange solid (0.224 g, 0.212 mmol, yield 59%).

***R*f** (CH_2Cl_2/CH_3OH 9:1) = 0.459; **^{1}H-NMR ($CDCl_3$) δ_H, ppm:** 7.65-7.79 (10H, m, 2xS-(*Ar*)C-CH-C$\underline{H}$-C-CH_2-(C=O), 4xCH_2-(*Ar*)C-C$\underline{H}$-CH-C-CF_3, 4xCH_2-(*Ar*)C-CH-C$\underline{H}$-C-CF_3), 7.39 (2H, d, 2xS-(*Ar*)C-CH-C$\underline{H}$-C-CH_2-(C=O), $^3J_{H,H}$ = 7.9 Hz), 5.18 (2H, d, 2xCH_3-(*Ar*)C-CH-C$\underline{H}$-C, $^3J_{H,H}$ = 5.4 Hz), 5.09 (2H, d, 2xCH_3-(*Ar*)C-C$\underline{H}$-CH-C, $^3J_{H,H}$ = 5.5 Hz), 4.96 (2H, d, 2xCH_3-(*Ar*)C-CH-C$\underline{H}$-C, $^3J_{H,H}$ = 5.4 Hz), 4.80 (2H, d, 2xCH_3-(*Ar*)C-C$\underline{H}$-CH-C, $^3J_{H,H}$ = 5.6 Hz), 3.90 (2H, s, S-(*Ar*)C-CH-CH-C-C$\underline{H_2}$-(C=O)), 3.75 (2H, s, S-C$\underline{H_2}$-(*Ar*)C-CH-CH-C-CF_3), 3.59 (2H, s, S-C$\underline{H_2}$-(*Ar*)C-CH-CH-C-CF_3), 1.84 (2H, sept, 2x(*Ar*)C-CH-CH-C-C$\underline{H}$(CH_3)$_2$, $^3J_{H,H}$ = 6.8 Hz), 1.73 (6H, s, 2xC$\underline{H_3}$-(*Ar*)C-CH-CH-C), 0.97 (6H, d, (*Ar*)C-CH-CH-C-CH(C$\underline{H_3}$)$_2$, $^3J_{H,H}$ = 6.8 Hz), 0.90 (6H, d, (*Ar*)C-CH-CH-C-CH(C$\underline{H_3}$)$_2$, $^3J_{H,H}$ = 6.8 Hz); **^{13}C-NMR ($CDCl_3$) δ_C, ppm:** 172.9 (1C, S-(*Ar*)C-CH-CH-C-CH_2-($\underline{C}$=O)), 144.1 (2C, 2xS-CH_2-(*Ar*)$\underline{C}$-CH-CH-C-CF_3), 136.6 (1C, S-(*Ar*)C-CH-CH-$\underline{C}$-CH_2-(C=O)), 135.1 (1C, S-(*Ar*)$\underline{C}$-CH-CH-C-CH_2-(C=O)), 132.5 (2C, 2xS-(*Ar*)C-CH-$\underline{C}$H-C-CH_2-(C=O)), 130.7 (2C, 2xS-(*Ar*)C-$\underline{C}$H-CH-C-CH_2-(C=O)), 130.5 (1C, m, S-CH_2-(*Ar*)C-CH-CH-$\underline{C}$-CF_3), 130.3 (1C, m, S-CH_2-(*Ar*)C-CH-CH-$\underline{C}$-CF_3), 130.2 (2C, 2xS-CH_2-(*Ar*)C-$\underline{C}$H-CH-C-CF_3), 129.9 (2C, 2xS-CH_2-(*Ar*)C-$\underline{C}$H-CH-C-CF_3), 125.9 (2C, 2xS-CH_2-(*Ar*)C-CH-$\underline{C}$H-C-CF_3, $^3J_{C,F}$ = 4 Hz), 125.7 (2C, 2xS-CH_2-(*Ar*)C-CH-$\underline{C}$H-C-CF_3, $^3J_{C,F}$ = 4 Hz), 124.1 (2C, qvart, 2xS-CH_2-(*Ar*)C-CH-CH-C-$\underline{C}F_3$, $^1J_{C,F}$ = 272 Hz), 107.7 (2C, 2xCH_3-(*Ar*)C-CH-CH-$\underline{C}$), 100.8 (2C, 2xCH_3-(*Ar*)$\underline{C}$-CH-CH-C), 84.1 (2C, 2xCH_3-(*Ar*)C-CH-$\underline{C}$H-C), 84.0 (2C, 2xCH_3-(*Ar*)C-CH-$\underline{C}$H-C), 83.9 (2C, 2xCH_3-(*Ar*)C-$\underline{C}$H-CH-C), 82.5 (2C, 2xCH_3-(*Ar*)C-$\underline{C}$H-CH-C), 41.9 (1C, S-(*Ar*)C-CH-CH-C-$\underline{C}H_2$-(C=O)), 40.1 (1C, S-$\underline{C}H_2$-(*Ar*)C-CH-CH-C-CF_3), 39.8 (1C, S-$\underline{C}H_2$-(*Ar*)C-CH-CH-C-CF_3), 31.0 (2C, 2x(*Ar*)CH-CH-C-$\underline{C}$H(CH_3)$_2$), 23.3 (2C, (*Ar*)CH-CH-C-CH($\underline{C}H_3$)$_2$), 22.6 (2C, (*Ar*)CH-CH-C-CH($\underline{C}H_3$)$_2$), 18.1 (2C, 2x$\underline{C}H_3$-(*Ar*)C-CH-CH); **^{19}F-NMR ($CDCl_3$) δ_F, ppm:** -62.29, -62.34 (6F, 2xC$\underline{F_3}$); **HRMS (ESI(+)):** *m/z* calcd for $C_{44}H_{47}F_6O_2Ru_2S_3^+$: 1021.0724 $[M-Cl]^+$; found: 1021.0756 (the isotopic pattern corresponds well to the calculated one); **elemental analysis** calcd (%) for $C_{44}H_{47}ClF_6O_2Ru_2S_3$: C 50.06, H 4.49; found C 51.55, H 4.50.

Synthesis of *tert*-butyl(3-(7-(diethylamino)-2-oxo-2*H*-chromene-3-carboxamido)propyl) carbamate (5)

To a solution of **Dye1-CO_2H** (0.500 g, 1.914 mmol, 1 equiv) in CH_2Cl_2 (100 mL) were added HOBt·H_2O (0.778 g, 4.594 mmol, 2.4 equiv) and DIPEA (0.84 mL, 4.785 mmol, 2.5 equiv). After 10 min were added successively EDCI (1.101 g, 5.742 mmol, 3 equiv), *tert*-butyl(3-aminopropyl)carbamate (0.333 g, 1.914 mmol, 1 equiv), and DIPEA (0.84 mL, 4.785 mmol, 2.5 equiv). The reaction mixture was stirred at room temperature for 24 h in the absence of light, and the reaction evolution was verified by TLC. The mixture was diluted with CH_2Cl_2 (50 mL) and washed with H_2O (150 mL). The isolated organic phase was further washed with brine (150 mL), dried over anhydrous Na_2SO_4, filtered, and concentrated under reduced pressure. Purification by flash column chromatography using an EtOAc/Hex mixture as eluent afforded **5** as a yellow solid (0.772 g, 1.850 mmol, yield 97%).

***R*f** (EtOAc/Hex 6:4) = 0.369; **^{1}H-NMR ($CDCl_3$) δ_H, ppm:** 8.86 (1H, t br, (C=O)-N$\underline{H}$-(CH_2)$_3$-NHBoc, $^3J_{H,H}$ = 5.4 Hz), 8.69 (1H, s, (*Coum*)O-C-C-C$\underline{H}$-C-(C=O)), 7.42 (1H, d, (*Coum*)O-C-C-C$\underline{H}$-CH-C-N(CH_2-CH_3)$_2$, $^3J_{H,H}$ = 9.0 Hz), 6.64 (1H, dd, (*Coum*)O-C-C-CH-C$\underline{H}$-C-N(CH_2-CH_3)$_2$, $^3J_{H,H}$ = 9.0 Hz, $^4J_{H,H}$ = 2.3 Hz), 6.49 (1H, d, (*Coum*)O-C-C$\underline{H}$-C-N(CH_2-CH_3)$_2$, $^4J_{H,H}$ = 2.3 Hz), 5.23 (1H, m, N$\underline{H}$Boc), 3.50 (2H, qvart, (C=O)NH-C$\underline{H_2}$-(CH_2)$_2$-NHBoc, $^3J_{H,H}$ = 6.4 Hz), 3.45 (4H, qvart, N(C$\underline{H_2}$-CH_3)$_2$, $^3J_{H,H}$ = 7.1 Hz), 3.18 (2H, qvart, (C=O)NH-(CH_2)$_2$-C$\underline{H_2}$-NHBoc, $^3J_{H,H}$ = 6.2 Hz), 1.75 (2H, qvin, (C=O)NH-CH_2-C$\underline{H_2}$-CH_2-NHBoc, $^3J_{H,H}$ = 6.5 Hz), 1.44 (9H, s, O-C(C$\underline{H_3}$)$_3$), 1.23 (6H, t, N(CH_2-C$\underline{H_3}$)$_2$, $^3J_{H,H}$ = 7.1 Hz); **^{13}C-NMR ($CDCl_3$) δ_C, ppm:** 163.9 (1C, (*Coum*)O-C-C-CH-C-($\underline{C}$=O)), 162.9

(1C, (*Coum*)O-C-C-CH-C-($\underline{C}$=O)-NH), 157.8 (1C, (*Coum*)O-$\underline{C}$-C-CH-C-(C=O)), 156.2 (1C, NH-($\underline{C}$=O)-O-C(CH_3)$_3$), 152.7 (1C, (*Ar*)$\underline{C}$-N(CH_2-CH_3), 148.3 (1C, (*Coum*)O-C-C-$\underline{C}$H-C-(C=O)), 131.3 (1C, (*Coum*)O-C-C-$\underline{C}$H-CH-C-N(CH_2CH_3)$_2$), 110.3 (1C, (*Coum*)O-C-$\underline{C}$-CH-C-(C=O)), 110.1 (1C, (*Coum*)O-C-C-CH-$\underline{C}$H-C-N(CH_2CH_3), 108.5 (1C, (*Coum*)O-C-C-CH-$\underline{C}$-(C=O)), 96.7 (1C, (*Coum*)O-C-$\underline{C}$H-C-N(CH_2CH_3)$_2$), 79.1 (1C, O-$\underline{C}$(CH_3)$_3$), 45.2 (2C, N($\underline{C}H_2$-CH_3)$_2$), 37.6 (1C, (C=O)-NH-(CH_2)$_2$-$\underline{C}H_2$-NHBoc), 36.7 (1C, (C=O)-NH-$\underline{C}H_2$-(CH_2)$_2$-NHBoc),30.4 (1C, (C=O)-NH-CH_2-$\underline{C}H_2$-CH_2-NHBoc), 28.6 (3C, O-C($\underline{C}H_3$)$_3$), 12.6 (2C, N(CH_2-$\underline{C}H_3$)$_2$); **HRMS (ESI(+)):** *m*/*z* calcd for $C_{22}H_{32}N_3O_5^+$: 418.2336 [M+H]$^+$, and for $C_{22}H_{31}N_3NaO_5^+$: 440.2156 [M+Na]$^+$; found: 418.2324, 440.2147 (the isotopic pattern corresponds well to the calculated one); **elemental analysis** calcd (%) for $C_{22}H_{31}N_3O_5$: C, 63.29; H, 7.48; N, 10.06; found: C 63.93, H 10.96, N 10.06.

Synthesis of *tert*-butyl (4-(7-(diethylamino)-2-oxo-2*H*-chromene-3-carboxamido)butyl) carbamate (6)

To a solution of **Dye1-CO_2H** (0.500 g, 1.914 mmol, 1 equiv) in CH_2Cl_2 (100 mL) were added HOBt·H_2O (0.778 g, 4.594 mmol, 2.4 equiv) and DIPEA (0.84 mL, 4.785 mmol, 2.5 equiv). After 10 min were added successively EDCI (1.101 g, 5.742 mmol, 3 equiv), *tert*-butyl(4-aminobutyl)carbamate (0.360 g, 1.914 mmol, 1 equiv), and DIPEA (0.84 mL, 4.785 mmol, 2.5 equiv). The reaction mixture was stirred at room temperature for 24 h in the absence of light, and the reaction evolution was verified by TLC. The mixture was diluted with CH_2Cl_2 (50 mL) and washed with H_2O (150 mL). The isolated organic phase was further washed with brine (150 mL), dried over anhydrous Na_2SO_4, filtered, and concentrated under reduced pressure. Purification by flash column chromatography using an EtOAc/Hex mixture as eluent afforded **6** as a yellow solid (0.793 g, 1.839 mmol, yield 96%).

***R*f** (EtOAc/Hex 6:4) = 0.328; **^{1}H NMR (CDCl$_3$) δ_H, ppm:** 8.81 (1H, t br, (C=O)-N$\underline{H}$-(CH_2)$_4$-NHBoc, $^3J_{H,H}$ = 5.5 Hz), 8.69 (1H, s, (*Coum*)O-C-C-C$\underline{H}$-C-(C=O)), 7.41 (1H, d, (*Coum*)O-C-C-C$\underline{H}$-CH-C-N(CH_2-CH_3)$_2$, $^3J_{H,H}$ = 9.0 Hz), 6.63 (1H, dd, (*Coum*)O-C-C-CH-C$\underline{H}$-C-N(CH_2-CH_3)$_2$, $^3J_{H,H}$ = 9.0 Hz, $^4J_{H,H}$ = 2.4 Hz), 6.49 (1H, d, (*Coum*)O-C-C$\underline{H}$-C-N(CH_2CH_3)$_2$, $^4J_{H,H}$ = 2.3 Hz), 4.59 (1H, m, N$\underline{H}$Boc), 3.45 (6H, qvart, N(C$\underline{H_2}$-CH_3)$_2$, (C=O)-NH-C$\underline{H_2}$-(CH_2)$_2$-NHBoc, $^3J_{H,H}$ = 7.1 Hz), 3.16 (2H, qvart, (C=O)-NH-(CH_2)$_2$-C$\underline{H_2}$-NHBoc, $^3J_{H,H}$ = 6.1 Hz), 1.60-1.66 (2H, m, (C=O)-NH-CH_2-C$\underline{H_2}$-(CH_2)$_2$-NHBoc), 1.54-1.60 (2H, m, (C=O)-NH-(CH_2)$_2$-C$\underline{H_2}$-CH_2-NHBoc), 1.43 (9H, s, O-C(C$\underline{H_3}$)$_3$), 1.23 (6H, t, N(CH_2-C$\underline{H_3}$)$_2$, $^3J_{H,H}$ = 7.1 Hz); **^{13}C NMR (CDCl$_3$) δ_C, ppm:** 163.9 (1C, (*Coum*)O-C-C-CH-C-($\underline{C}$=O)), 162.9 (1C, (*Coum*)O-C-C-CH-C-($\underline{C}$=O)-NH), 157.7 (1C, (*Coum*)O-$\underline{C}$-C-CH-C-(C=O)), 156.1 (1C, NH-($\underline{C}$=O)-O-C(CH_3)$_3$), 152.6 (1C, (*Ar*)$\underline{C}$-N(CH_2-CH_3), 148.2 (1C, (*Coum*)O-C-C-$\underline{C}$H-C-(C=O)), 131.2 (1C, (*Coum*)O-C-C-$\underline{C}$H-CH-C-N(CH_2-CH_3)$_2$), 110.5 (1C, (*Coum*)O-C-$\underline{C}$-CH-C-(C=O)), 110.1 (1C, (*Coum*)O-C-C-CH-$\underline{C}$H-C-N(CH_2-CH_3), 108.5 (1C, (*Coum*)O-C-C-CH-$\underline{C}$-(C=O)), 96.7 (1C, (*Coum*)O-C-$\underline{C}$H-C-N(CH_2-CH_3)$_2$), 79.2 (1C, O-$\underline{C}$(CH_3)$_3$), 45.2 (2C, N($\underline{C}H_2$-CH_3)$_2$), 40.4 (1C, (C=O)-NH-(CH_2)$_3$-$\underline{C}H_2$-NHBoc), 39.4 (1C, (C=O)-NH-$\underline{C}H_2$-(CH_2)$_3$-NHBoc), 28.6 (3C, O-C($\underline{C}H_3$)$_3$), 27.7 (1C, (C=O)-NH-(CH_2)$_2$-$\underline{C}H_2$-CH_2-NHBoc), 27.2 (1C, (C=O)-NH-CH_2-$\underline{C}H_2$-(CH_2)$_2$-NHBoc), 12.6 (2C, N(CH_2-$\underline{C}H_3$)$_2$); **HRMS (ESI(+)):** *m*/*z* calcd for $C_{23}H_{34}N_3O_5^+$: 432.2493 [M+H]$^+$, and for $C_{23}H_{33}N_3NaO_5^+$: 454.2312 [M+Na]$^+$; found: 432.2481, 454.2302 (the isotopic pattern corresponds well to the calculated one); **elemental analysis** calcd (%) for $C_{23}H_{33}N_3O_5$: C 64.02, H 7.71, N 9.74; found: C 64.19, H, 11.45, N 9.74.

Synthesis of *tert*-butyl(4-(11-oxo-2,3,6,7-tetrahydro-1*H*,5*H*,11*H*-pyrano[2,3-*f*]pyrido[3,2,1-*ij*]quinoline-10-carboxamido)butyl)carbamate (7)

To a solution of **Dye2-CO2H** (0.460 g, 1.612 mmol, 1 equiv) in CH_2Cl_2 (100 mL) were added HOBt·H_2O (0.655 g, 3.869 mmol, 2.4 equiv) and DIPEA (0.71 mL, 4.030 mmol, 2.5 equiv). After 10 min were added successively EDCI (0.927 g, 4.836 mmol, 3 equiv), *tert*-butyl(4-aminobutyl)carbamate (0.304 g, 1.612 mmol, 1 equiv), and DIPEA (0.71 mL, 4.030 mmol, 2.5 equiv). The reaction mixture was stirred at room temperature for 24 h in the absence of light, and the reaction evolution was verified by TLC. The mixture was diluted with CH_2Cl_2 (50 mL) and washed with H_2O (150 mL). The isolated organic phase was further washed with brine (150 mL), dried over anhydrous Na_2SO_4, filtered, and concentrated under reduced pressure. Purification by flash column chromatography using an EtOAc/Hex mixture as eluent afforded **7** as a yellow solid (0.526 g, 1.156 mmol, yield 72%).

***R*f** (EtOAc/Hex 8:2) = 0.407; **^{1}H-NMR ($CDCl_3$) δ_H, ppm:** 8.87 (1H, t br, (C=O)-N*H*-(CH_2)$_4$-NHBoc, $^3J_{H,H}$ = 5.5 Hz), 8.59 (1H, s, (*Coum*)O-C-C-C*H*-C-(C=O)), 7.00 (1H, s, (*Coum*)O-C-C-C*H*-C-CH_2), 3.44 (2H, qvart, NH-C*H2*-(CH_2)$_3$-NHBoc, $^3J_{H,H}$ = 6.1 Hz), 3.33 (2H, t, (*Coum*)O-C-C-CH-C-(CH_2)$_2$-C*H2*-N, $^3J_{H,H}$ = 4.8 Hz), 3.31 (2H, t, (*Coum*)O-C-C-(CH_2)$_2$-C*H2*-N, $^3J_{H,H}$ = 4.4 Hz), 3.15 (2H, qvart br, NH-(CH_2)$_3$-C*H2*-NHBoc, $^3J_{H,H}$ = 6.1 Hz), 2.88 (2H, t, (*Coum*)O-C-C-CH-C-C*H2*-(CH_2)$_2$-N, $^3J_{H,H}$ = 6.4 Hz), 2.77 (2H, t, (*Coum*)O-C-C-C*H2*-(CH_2)$_2$-N, $^3J_{H,H}$ = 6.1 Hz), 1.93-2.01 (4H, m, (*Coum*)O-C-C-CH-C-CH_2-C*H2*-CH_2-N, (*Coum*)O-C-C-CH_2-C*H2*-CH_2-N), 1.54-1.68 (4H, m, NH-CH_2-C*H2*-(CH_2)$_2$-NHBoc, NH-(CH_2)$_2$-C*H2*-CH_2-NHBoc), 1.43 (9H, s, O-C(C*H3*)$_3$); **^{13}C-NMR ($CDCl_3$) δ_C, ppm:** 163.7 (1C, (*Coum*)O-C-C-CH-C-(*C*=O)-NH), 163.2 (1C, (*Coum*)O-C-C-CH-C-(*C*=O)), 156.1 (1C, NH-(*C*=O)-O-C(CH_3)$_3$), 152.8 (1C, (*Coum*)O-*C*-C-CH-C-(C=O)), 148.20 (1C, (*Ar*)*C*-N-CH_2-CH_2), 148.16 (1C, (*Coum*)O-C-C-*C*H-C-(C=O)-NH), 127.1 (1C, (*Coum*)O-C-C-*C*H-C-(CH_2)$_3$-N), 119.7 (1C, (*Coum*)O-C-*C*-(CH_2)$_3$-N), 109.3 (1C, (*Coum*)O-C-C-CH-*C*-(C=O)-NH), 108.4 (1C, (*Coum*)O-C-*C*-CH-C-(C=O)-NH), 105.8 (1C, (*Coum*)O-C-C-CH-*C*-(CH_2)$_3$-N), 79.2 (1C, O-*C*(CH_3)$_3$), 50.4 (1C, (*Coum*)O-C-C-CH-C-(CH_2)$_2$-*C*H_2-N), 49.9 (1C, (*Coum*)O-C-C-(CH_2)$_2$-*C*H_2-N), 40.4 (1C, NH-*C*H_2-(CH_2)$_3$-NHBoc), 39.3 (1C, NH-(CH_2)$_3$-*C*H_2-NHBoc), 28.6 (3C, O-C(*C*H_3)$_3$), 27.7 (1C, NH-CH_2-*C*H_2-(CH_2)$_2$-NHBoc), 27.6 (1C, (*Coum*)O-C-C-CH-C-*C*H_2-(CH_2)$_2$-N), 27.2 (1C, NH-(CH_2)$_2$-*C*H_2-CH_2-NHBoc), 21.3 (1C, (*Coum*)O-C-C-CH-C-CH_2-*C*H_2-CH_2-N), 20.4 (1C, (*Coum*)O-C-C-*C*H_2-(CH_2)$_2$-N), 20.3 (1C, (*Coum*)O-C-C-CH_2-*C*H_2-CH_2-N); **HRMS (ESI(+)):** *m*/*z*, calcd for $C_{25}H_{34}N_3O_5^+$: 456.2493 $[M+H]^+$, and $C_{25}H_{33}N_3O_5Na^+$: 478.2312 $[M+Na]^+$; found: 456.2485, 478.2303 (the isotopic pattern corresponds well to the calculated one); **elemental analysis** calcd (%) for $C_{25}H_{33}N_3O_5$·0.5CH_3OH: C 64.95, H 7.48, N 8.91; found: C 64.98, H 10.50, N 8.91.

The NH-Boc group in compounds **5**, **6** and **7** were deprotected by adapting formerly published protocols.[8]

Synthesis of 3-(7-(diethylamino)-2-oxo-2*H*-chromene-3-carboxamido)propan-1-aminium-2,2,2-trifluoroacetate (8)

TFA (5 mL) was dropwise added to a stirred solution of **5** (0.722 g, 1.729 mmol) in 15 mL dry CH_2Cl_2 at room temperature and the reaction mixture was stirred at room temperature for 2 h in the absence of light. The reaction evolution was verified by TLC, the solvent was removed under reduced pressure and the crude was resolubilized in MeOH (3 × 50 mL) and reconcentrated. To a stirred solution of the crude in MeOH (50 mL) Na_2CO_3 (2 g) was added in small portions, the solution was recovered by filtration and concentrated under reduced pressure. Purification by flash column chromatography using a CH_2Cl_2/MeOH mixture as eluent. A solution of the purified compound in 50 mL MeOH was added to 5 g of Amberylst 21 and the suspension was stirred at room temperature for 30 min. The solution was recovered by filtration and the resin beads were further washed with MeOH (3 × 50 mL),

and the unified solution was concentrated under reduced pressure and the recovered yellow-orange solid (quant. yield) was used for the coupling reaction.

***R*f** (CH_2Cl_2/CH_3OH 10:1) = 0.500; **^{1}H NMR (MeOD-*d$_4$*) δ*$_H$*, ppm:** 8.61 (1H, s, (*Coum*)O-C-C-C$\underline{H}$-C-(C=O)), 7.53 (1H, d, (*Coum*)O-C-C-C$\underline{H}$-CH-C-N(CH_2-CH_3)$_2$, $^3J_{H,H}$ = 9.1 Hz), 6.80 (1H, dd, (*Coum*)O-C-C-CH-C$\underline{H}$-C-N(CH_2-CH_3)$_2$, $^3J_{H,H}$ = 9.1 Hz, $^4J_{H,H}$ = 2.4 Hz), 6.53 (1H, d, (*Coum*)O-C-C$\underline{H}$-C-N(CH_2CH_3)$_2$, $^4J_{H,H}$ = 2.3 Hz), 3.53 (2H, t, (C=O)-NH-C$\underline{H_2}$-(CH_2)$_2$-NH_2, $^3J_{H,H}$ = 6.6 Hz), 3.51 (4H, qvart, N(C$\underline{H_2}$-CH_3)$_2$, $^3J_{H,H}$ = 7.1 Hz), 3.01 (2H, t, (C=O)-NH-(CH_2)$_2$-C$\underline{H_2}$-NH_2, $^3J_{H,H}$ = 7.2 Hz), 1.98 (2H, qvin, (C=O)-NH-CH_2-C$\underline{H_2}$-CH_2-NH_2, $^3J_{H,H}$ = 7.0 Hz), 1.23 (6H, t, N(CH_2-C$\underline{H_3}$)$_2$, $^3J_{H,H}$ = 7.1 Hz); **^{13}C NMR (MeOD-*d$_4$*) δ*$_C$*, ppm:** 166.3 (1C, (*Coum*)O-C-C-CH-C-($\underline{C}$=O)-NH), 163.9 (1C, (*Coum*)O-C-C-CH-C-($\underline{C}$=O)), 163.5 (1C, CF_3-$\underline{C}O_2H$, $^2J_{C,F}$ = 35 Hz), 159.2 (1C, (*Coum*)O-$\underline{C}$-C-CH-C-(C=O)), 154.7 (1C, (*Ar*)$\underline{C}$-N(CH_2-CH_3), 149.4 (1C, (*Coum*)O-C-C-$\underline{C}$H-C-(C=O)), 132.7 (1C, (*Coum*)O-C-C-$\underline{C}$H-CH-C-N(CH_2-CH_3)$_2$), 117.9 (1C, $\underline{C}F_3$-CO_2H, $^1J_{C,F}$ = 290 Hz), 111.8 (1C, (*Coum*)O-C-C-CH-$\underline{C}$H-C-N(CH_2-CH_3), 109.5 (1C, (*Coum*)O-C-$\underline{C}$-CH-C-(C=O)), 109.4 (1C, (*Coum*)O-C-C-CH-$\underline{C}$-(C=O)), 97.2 (1C, (*Coum*)O-C-$\underline{C}$H-C-N(CH_2-CH_3)$_2$), 46.0 (2C, N($\underline{C}H_2$-CH_3)$_2$), 38.3 (1C, (C=O)-NH-(CH_2)$_2$-$\underline{C}H_2$-NH_2), 37.1 (1C, (C=O)-NH-$\underline{C}H_2$-(CH_2)$_2$-NH_2), 28.9 (1C, (C=O)-NH-CH_2-$\underline{C}H_2$-CH_2-NH_2), 12.7 (2C, N(CH_2-$\underline{C}H_3$)$_2$); **^{19}F NMR (MeOD-*d$_4$*) δ*$_F$*, ppm:** -77.19 (3F, C$\underline{F_3}$$CO_2H$); **HRMS (ESI(+)):** *m/z* calcd for $C_{17}H_{24}N_3O_3^+$: 318.1812 [M-$CF_3CO_2^-$]$^+$; found: 318.1801 (the isotopic pattern corresponds well to the calculated one); **elemental analysis** calcd (%) for $C_{17}H_{23}N_3O_3 \cdot 11CF_3CO_2H \cdot 0.5CH_2Cl_2 \cdot 30H_2O$: C 22.02, H 4.44, N 1.95; found C 22.04, H 4.09, N 1.94.

Synthesis of 4-(7-(diethylamino)-2-oxo-2*H*-chromene-3-carboxamido)butan-1-aminium-2,2,2-trifluoroacetate (9)

TFA (5 mL) was dropwise added to a stirred solution of **6** (0.743 g, 1.722 mmol) in 15 mL dry CH_2Cl_2 at room temperature and the reaction mixture was stirred at room temperature for 2 h in the absence of light. The reaction evolution was verified by TLC, the solvent was removed under reduced pressure and the crude was resolubilized in MeOH (3 × 50 mL) and reconcentrated. To a stirred solution of the crude in MeOH (50 mL) Na_2CO_3 (2 g) was added in small portions, the solution was recovered by filtration and concentrated under reduced pressure. Purification by flash column chromatography using a CH_2Cl_2/MeOH mixture as eluent. A solution of the isolated purified compound in 50 mL MeOH was added to 5 g of Amberylst 21 and the suspension was stirred at room temperature for 30 min. The solution was recovered by filtration and the resin beads were further washed with MeOH (3 × 50 mL), and the unified solution was concentrated under reduced pressure and the recovered yellow-orange solid (0.692 g, 1.616 mmol, yield 94%) was used for the coupling reaction.

***R*f** (CH_2Cl_2/CH_3OH 10:1) = 0.516; **^{1}H NMR (MeOD-*d$_4$*) δ*$_H$*, ppm:** 8.60 (1H, s, (*Coum*)O-C-C-C$\underline{H}$-C-(C=O)), 7.53 (1H, d, (*Coum*)O-C-C-C$\underline{H}$-CH-C-N(CH_2-CH_3)$_2$, $^3J_{H,H}$ = 9.0 Hz), 6.81 (1H, dd, (*Coum*)O-C-C-CH-C$\underline{H}$-C-N(CH_2-CH_3)$_2$, $^3J_{H,H}$ = 9.0 Hz, $^4J_{H,H}$ = 2.4 Hz), 6.54 (1H, d, (*Coum*)O-C-C$\underline{H}$-C-N(CH_2-CH_3)$_2$, $^4J_{H,H}$ = 2.3 Hz), 3.52 (4H, qvart, N(C$\underline{H_2}$-CH_3)$_2$, $^3J_{H,H}$ = 7.1 Hz), 3.46 (2H, t, (C=O)-NH-(CH_2)$_3$-C$\underline{H_2}$-NH_2, $^3J_{H,H}$ = 6.6 Hz), 2.99 (2H, t, (C=O)-NH-C$\underline{H_2}$-(CH_2)$_3$-NH_2, $^3J_{H,H}$ = 7.1 Hz), 1.66-1.79 (4H, m, (C=O)-NH-CH_2-C$\underline{H_2}$-(CH_2)$_2$-NHBoc), (C=O)-NH-(CH_2)$_2$-C$\underline{H_2}$-CH_2-NHBoc), 1.23 (6H, t, N(CH_2-C$\underline{H_3}$)$_2$, $^3J_{H,H}$ = 7.1 Hz); **^{13}C NMR (MeOD-*d$_4$*) δ*$_C$*, ppm:** 165.6 (1C, (*Coum*)O-C-C-CH-C-($\underline{C}$=O)-NH), 164.0 (1C, (*Coum*)O-C-C-CH-C-($\underline{C}$=O)-O), 163.5 (1C, CF_3-$\underline{C}O_2H$, $^2J_{C,F}$ = 35 Hz), 159.1 (1C, (*Coum*)O-$\underline{C}$-C-CH-C-(C=O)), 154.7 (1C, (*Ar*)$\underline{C}$-N(CH_2-CH_3), 149.3 (1C, (*Coum*)O-C-C-$\underline{C}$H-C-(C=O)), 132.6 (1C, (*Coum*)O-C-C-$\underline{C}$H-CH-C-N(CH_2-CH_3)$_2$), 118.0 (1C, $\underline{C}F_3$-CO_2H, $^1J_{C,F}$ = 290 Hz), 111.7 (1C, (*Coum*)O-C-C-CH-$\underline{C}$H-C-N(CH_2-CH_3), 109.9 (1C,

(*Coum*)O-C-$\underline{C}$-CH-C-(C=O)), 109.4 (1C, (*Coum*)O-C-C-CH-$\underline{C}$-(C=O)), 97.2 (1C, (*Coum*)O-C-$\underline{C}$H-C-N(CH$_2$-CH$_3$)$_2$), 46.0 (2C, N($\underline{C}$H$_2$-CH$_3$)$_2$), 40.4 (1C, (C=O)-NH-(CH$_2$)$_3$-$\underline{C}$H$_2$-NH$_2$), 39.7 (1C, (C=O)-NH-$\underline{C}$H$_2$-(CH$_2$)$_3$-NH$_2$), 27.5 (1C, (C=O)-NH-(CH$_2$)$_2$-$\underline{C}$H$_2$-CH$_2$-NH$_2$), 26.0 (1C, (C=O)-NH-CH$_2$-$\underline{C}$H$_2$-(CH$_2$)$_2$-NH$_2$), 12.7 (2C, N(CH$_2$-$\underline{C}$H$_3$)$_2$); **^{19}F NMR (MeOD-*d$_4$*) δ_F, ppm:** -77.19 (3F, C$\underline{F_3}$CO$_2$H); **HRMS (ESI(+)):** *m/z* calcd for $C_{18}H_{26}N_3O_3^+$: 332.1969 [M-$CF_3CO_2^-$]$^+$; found: 332.1952 (the isotopic pattern corresponds well to the calculated one); **elemental analysis** calcd (%) for $C_{18}H_{25}N_3O_3\cdot 12CF_3CO_2H\cdot CH_3OH\cdot 10H_2O$: C 27.01, H 3.22, N, 2.20; found C 27.15, H 3.38, N 2.18.

Synthesis of 4-(11-oxo-2,3,6,7-tetrahydro-1*H*,5*H*,11*H*-pyrano[2,3-*f*]pyrido[3,2,1-*ij*]quinoline-10-carboxamido)butan-1-aminium-2,2,2-trifluoroacetate (10)

TFA (5 mL) was dropwise added to a stirred solution of **7** (0.486 g, 1.068 mmol) in 15 mL dry CH_2Cl_2 at room temperature and the reaction mixture was stirred at room temperature for 2 h in the absence of light. The reaction evolution was verified by TLC, the solvent was removed under reduced pressure and the crude was resolubilized in MeOH (3 × 50 mL) and reconcentrated. To a stirred solution of the crude in MeOH (50 mL) Na_2CO_3 (2 g) was added in small portions, the solution was recovered by filtration and concentrated under reduced pressure. Purification by flash column chromatography using a CH_2Cl_2/MeOH mixture as eluent. A solution of the purified compound in 50 mL MeOH was added to 5 g of Amberylst 21 and the suspension was stirred at room temperature for 30 min. The solution was recovered by filtration and the resin beads were further washed with MeOH (3 × 50 mL), and the unified solution was concentrated under reduced pressure and the recovered yellow-orange solid (0.415 g, 0.915 mmols, yield 86%) was used for the coupling reaction.

***R*f** (CH_2Cl_2/CH_3OH 10:1) = 0.424; **^{1}H NMR (MeOD-*d$_4$*) δ_H, ppm:** 8.50 (1H, s, (*Coum*)O-C-C-C$\underline{H}$-C-(C=O)), 7.12 (1H, s, (*Coum*)O-C-C-C$\underline{H}$-C-CH$_2$), 3.46 (4H, t, C(*Coum*)-(C=O)-NH-C$\underline{H_2}$-(CH$_2$)$_3$-NH$_2$, $^3J_{H,H}$ = 6.6 Hz), 3.36-3.41 (4H, m, (*Coum*)O-C-C-CH-C-(CH$_2$)$_2$-C$\underline{H_2}$-N, (*Coum*)O-C-C-(CH$_2$)$_2$-C$\underline{H_2}$-N), 2.99 (2H, t, C(*Coum*)-(C=O)-NH-(CH$_2$)$_3$-C$\underline{H_2}$-NH$_2$, $^3J_{H,H}$ = 7.4 Hz), 2.83 (2H, t, (*Coum*)O-C-C-CH-C-C$\underline{H_2}$-(CH$_2$)$_2$-N, $^3J_{H,H}$ = 6.5 Hz), 2.78 (2H, t, (*Coum*)O-C-C-C$\underline{H_2}$-(CH$_2$)$_2$-N, $^3J_{H,H}$ = 6.2 Hz), 1.92-2.01 (4H, m, (*Coum*)O-C-C-CH-C-CH$_2$-C$\underline{H_2}$-CH$_2$-N, (*Coum*)O-C-C-CH$_2$-C$\underline{H_2}$-CH$_2$-N), 1.65-1.78 (4H, m, C(*Coum*)-(C=O)-NH-CH$_2$-C$\underline{H_2}$-(CH$_2$)$_2$-NH$_2$, C(*Coum*)-(C=O)-NH-(CH$_2$)$_2$-C$\underline{H_2}$-CH$_2$-NH$_2$); **^{13}C NMR (MeOD-*d$_4$*) δ_C, ppm:** 166.1 (1C, (*Coum*)O-C-C-CH-C-($\underline{C}$=O)-NH), 164.4 (1C, (*Coum*)O-C-C-CH-C-($\underline{C}$=O)), 163.6 (1C, CF$_3$-$\underline{C}$O$_2$H, $^2J_{C,F}$ = 35 Hz), 154.0 (1C, (*Coum*)O-$\underline{C}$-C-CH-C-(C=O)), 150.3 (1C, (*Ar*)$\underline{C}$-N-CH$_2$-CH$_2$), 149.2 (1C, (*Coum*)O-C-C-$\underline{C}$H-C-(C=O)), 128.5 (1C, (*Coum*)O-C-C-$\underline{C}$H-C-(CH$_2$)$_3$-N), 121.9 (1C, (*Coum*)O-C-$\underline{C}$-(CH$_2$)$_3$-N), 117.9 (1C, $\underline{C}$F$_3$-CO$_2$H, $^1J_{C,F}$ = 290 Hz), 109.3 (1C, (*Coum*)O-C-C-CH-$\underline{C}$-(C=O)-NH), 108.0 (1C, (*Coum*)O-C-$\underline{C}$-CH-C-(C=O)-NH), 106.5 (1C, (*Coum*)O-C-C-CH-$\underline{C}$-(CH$_2$)$_3$-N), 51.3 (1C, (*Coum*)O-C-C-CH-C-(CH$_2$)$_2$-$\underline{C}$H$_2$-N), 50.8 (1C, (*Coum*)O-C-C-(CH$_2$)$_2$-$\underline{C}$H$_2$-N), 40.4 (1C, C(*Coum*)-(C=O)-NH-(CH$_2$)$_3$-$\underline{C}$H$_2$-NH$_2$), 39.7 (1C, C(*Coum*)-(C=O)-NH-$\underline{C}$H$_2$-(CH$_2$)$_3$-NH$_2$), 28.4 (1C, (*Coum*)O-C-C-CH-C-$\underline{C}$H$_2$-(CH$_2$)$_2$-N), 27.6 (1C, C(*Coum*)-(C=O)-NH-(CH$_2$)$_2$-$\underline{C}$H$_2$-CH$_2$-NH$_2$), 26.0 (1C, C(*Coum*)-(C=O)-NH-CH$_2$-$\underline{C}$H$_2$-(CH$_2$)$_2$-NH$_2$), 22.1 (1C, (*Coum*)O-C-C-CH-C-CH$_2$-$\underline{C}$H$_2$-CH$_2$-N), 21.2 (1C, (*Coum*)O-C-C-CH$_2$-$\underline{C}$H$_2$-CH$_2$-N), 21.0 (1C, (*Coum*)O-C-C-$\underline{C}$H$_2$-(CH$_2$)$_2$-N); **^{19}F NMR (MeOD-*d$_4$*) δ_F, ppm:** -77.22 (3F, C$\underline{F_3}$CO$_2$H); **HRMS (ESI(+)):** *m/z* calcd for $C_{20}H_{26}N_3O_3^+$: 356.1969 [M-$CF_3CO_2^-$]$^+$; found: 356.1953 (the isotopic pattern corresponds well to the calculated one); **elemental analysis** calcd (%) for $C_{20}H_{25}N_3O_3\cdot 6CF_3CO_2H\cdot 11H_2O$: C 31.05, H 4.32, N 3.39; found: C 31.26, H 4.28, N 3.40.

Synthesis of [(η^6-*p*-MeC$_6$H$_4$^{i}Pr)$_2$Ru$_2$(SCH$_2$C$_6$H$_4$^{t}Bu)$_2$(SC$_6$H$_4$-*p*-OR)]Cl (R = 7-(diethylamino)-2-oxo-2*H*-chromene-3-carboxylate) (11)

To a solution of **Dye1-CO$_2$H** (0.070 g, 0.253 mmol, 1.25 equiv) in dry CH_2Cl_2 (100 mL) were added successively EDCI (0.049 g, 0.253 mmol, 1.25 equiv), **2a** (0.200 g, 0.202 mmol, 1 equiv), and DMAP (0.006 g, 0.051 mmol, 0.25 equiv). The reaction mixture was stirred at room temperature for 24 h, the reaction evolution was verified by ^{1}H NMR ($CDCl_3$) and TLC, the mixture was concentrated to dryness under reduced pressure. Purification by flash column chromatography using a CH_2Cl_2/MeOH mixture as eluent afforded **11** as an orange solid (0.247 g, 0.201 mmol, yield 99%). ***R*$_f$** (CH_2Cl_2/MeOH 10:1) = 0.483; **^{1}H-NMR ($CDCl_3$) δ_H, ppm:** 8.67 (1H, s, (*Coum*)O-C-C-C$\underline{H}$-C-(C=O)), 7.79 (2H, d, 2xS-(*Ar*)C-C$\underline{H}$-CH-C-O, $^3J_{H,H}$ = 8.7 Hz), 7.40-7.49 (9H, m, 4xCH$_2$-(*Ar*)C-C$\underline{H}$-CH-C-C(CH$_3$)$_3$, 4xCH$_2$-(*Ar*)C-CH-C$\underline{H}$-C-C(CH$_3$)$_3$, (*Coum*)O-C-C-C$\underline{H}$-CH-C-N(CH$_2$-CH$_3$)$_2$), 7.19 (2H, d, 2xS-(*Ar*)C-CH-C$\underline{H}$-C-O, $^3J_{H,H}$ = 8.7 Hz), 6.67 (1H, dd, (*Coum*)O-C-C-CH-C$\underline{H}$-C-N(CH$_2$-CH$_3$)$_2$, $^3J_{H,H}$ = 9.0 Hz, $^4J_{H,H}$ = 2.4 Hz), 6.49 (1H, d, (*Coum*)O-C-C$\underline{H}$-C-N(CH$_2$-CH$_3$)$_2$, $^4J_{H,H}$ = 2.3 Hz), 5.11 (2H, d, 2xCH$_3$-(*Ar*)C-CH-C$\underline{H}$-C, $^3J_{H,H}$ = 5.7 Hz), 5.01 (2H, d, 2xCH$_3$-(*Ar*)C-CH-C$\underline{H}$-C, $^3J_{H,H}$ = 5.8 Hz), 4.89 (2H, d, 2xCH$_3$-(*Ar*)C-C$\underline{H}$-CH-C, $^3J_{H,H}$ = 5.8 Hz), 4.64 (2H, d, 2xCH$_3$-(*Ar*)C-C$\underline{H}$-CH-C, $^3J_{H,H}$ = 5.8 Hz), 3.61 (2H, s, S-C$\underline{H_2}$-(*Ar*)C-CH-CH-C-C(CH$_3$)$_3$), 3.47 (4H, qvart, N(C$\underline{H_2}$-CH$_3$)$_2$, $^3J_{H,H}$ = 7.1 Hz), 3.43 (2H, s, S-C$\underline{H_2}$-(*Ar*)C-CH-CH-C-C(CH$_3$)$_3$), 1.93 (2H, sept, 2x(*Ar*)C-CH-CH-C-C$\underline{H}$(CH$_3$)$_2$, $^3J_{H,H}$ = 6.9 Hz), 1.77 (6H, s, 2xC$\underline{H_3}$-(*Ar*)C-CH-CH-C), 1.36 (9H, s, S-CH$_2$-(*Ar*)C-CH-CH-C-C(C$\underline{H_3}$)$_3$), 1.33 (9H, s, S-CH$_2$-(*Ar*)C-CH-CH-C-C(C$\underline{H_3}$)$_3$), 1.25 (6H, t, N(CH$_2$-C$\underline{H_3}$)$_2$, $^3J_{H,H}$ = 7.1 Hz), 0.97 (6H, d, (*Ar*)C-CH-CH-C-CH(C$\underline{H_3}$)$_2$, $^3J_{H,H}$ = 6.9 Hz), 0.91 (6H, d, (*Ar*)C-CH-CH-C-CH(C$\underline{H_3}$)$_2$, $^3J_{H,H}$ = 6.9 Hz); **^{13}C-NMR ($CDCl_3$) δ_C, ppm:** 162.2 (1C, S-(*Ar*)C-CH-CH-C-O-($\underline{C}$=O)), 159.0 (1C, (*Coum*)O-$\underline{C}$-C-CH-C-(C=O)), 158.4 (1C, (*Coum*)O-C-C-CH-C-($\underline{C}$=O)), 153.7 (1C, (*Ar*)$\underline{C}$-N(CH$_2$-CH$_3$), 151.9, 151.8 (2C, 2xS-CH$_2$-(*Ar*)C-CH-CH-$\underline{C}$-C(CH$_3$)$_3$), 151.4 (1C, S-(*Ar*)C-CH-CH-$\underline{C}$-O-(C=O)), 150.6 (1C, (*Coum*)O-C-C-$\underline{C}$H-C-(C=O)), 136.8, 136.7 (2C, 2xS-CH$_2$-(*Ar*)$\underline{C}$-CH-CH-C-C(CH$_3$)$_3$), 134.9 (1C, S-(*Ar*)$\underline{C}$-CH-CH-C-O-(C=O)), 133.6 (2C, 2xS-(*Ar*)C-$\underline{C}$H-CH-C-O-(C=O)), 131.9 (1C, (*Coum*)O-C-C-$\underline{C}$H-CH-C-N(CH$_2$-CH$_3$)$_2$), 129.4, 129.2 (4C, 4xS-CH$_2$-(*Ar*)C-$\underline{C}$H-CH-C-C(CH$_3$)$_3$), 125.7, 125.6 (4C, 4xS-CH$_2$-(*Ar*)C-CH-$\underline{C}$H-C-C(CH$_3$)$_3$), 122.8 (2C, 2xS-(*Ar*)C-CH-$\underline{C}$H-C-O-(C=O), 110.2 (1C, (*Coum*)O-C-C-CH-$\underline{C}$H-C-N(CH$_2$-CH$_3$), 108.0 (1C, (*Coum*)O-C-$\underline{C}$-CH-C-(C=O)), 107.5 (2C, 2xCH$_3$-(*Ar*)C-CH-CH-$\underline{C}$), 106.8 (1C, (*Coum*)O-C-C-CH-$\underline{C}$-(C=O)), 100.5 (2C, 2xCH$_3$-(*Ar*)$\underline{C}$-CH-CH-C), 96.8 (1C, (*Coum*)O-C-$\underline{C}$H-C-N(CH$_2$CH$_3$)$_2$), 84.0 (4C, 4xCH$_3$-(*Ar*)C-CH-$\underline{C}$H-C), 83.9 (2C, 2xCH$_3$-(*Ar*)C-$\underline{C}$H-CH-C), 82.6 (2C, 2xCH$_3$-(*Ar*)C-$\underline{C}$H-CH-C), 45.4 (2C, N($\underline{C}$H$_2$-CH$_3$)$_2$), 40.2 (1C, S-$\underline{C}$H$_2$-(*Ar*)C-CH-CH-C-C(CH$_3$)$_3$), 39.6 (1C, S-$\underline{C}$H$_2$-(*Ar*)C-CH-CH-C-C(CH$_3$)$_3$), 34.93 (1C, S-CH$_2$-(*Ar*)C-CH-CH-C-$\underline{C}$(CH$_3$)$_3$), 34.89 (1C, S-CH$_2$-(*Ar*)C-CH-CH-C-$\underline{C}$(CH$_3$)$_3$), 31.57 (3C, S-CH$_2$-(*Ar*)C-CH-CH-C-C($\underline{C}$H$_3$)$_3$), 31.54 (3C, S-CH$_2$-(*Ar*)C-CH-CH-C-C($\underline{C}$H$_3$)$_3$), 31.0 (2C, 2x(*Ar*)CH-CH-C-$\underline{C}$H(CH$_3$)$_2$), 23.2 (2C, (*Ar*)CH-CH-C-CH($\underline{C}$H$_3$)$_2$), 22.8 (2C, (*Ar*)CH-CH-C-CH($\underline{C}$H$_3$)$_2$), 18.3 (2C, 2x$\underline{C}$H$_3$-(*Ar*)C-CH-CH), 12.6 (2C, N(CH$_2$-$\underline{C}$H$_3$)$_2$); **HRMS (ESI(+)):** *m/z* calcd for $C_{62}H_{76}NO_4Ru_2S_3^+$: 1198.3018 $[M-Cl]^+$; found: 1198.3052 (the isotopic pattern corresponds well to the calculated one); **elemental analysis** calcd (%) for $C_{62}H_{76}ClNO_4Ru_2S_3 \cdot 3CH_3OH$: C 58.74, H 6.67, N 1.05; found C 58.81, H 6.68, N 1.10.

Synthesis of [(η^6-*p*-MeC$_6$H$_4$^{i}Pr)$_2$Ru$_2$(SCH$_2$C$_6$H$_4$^{t}Bu)$_2$(SC$_6$H$_4$-*p*-OR)]Cl (R = 11-oxo-2,3,6,7-tetrahydro-1*H*,5*H*,11*H*-pyrano[2,3-*f*]pyrido[3,2,1-*ij*]quinoline-10-carboxylate) (12a)

To a solution of **Dye2-CO$_2$H** (0.072 g, 0.253 mmol, 1.25 equiv) in dry CH_2Cl_2 (100 mL) were added successively EDCI (0.050 g, 0.263 mmol, 1.30 equiv), **2a** (0.200 g, 0.202 mmol, 1 equiv), and DMAP (0.030 g, 0.242 mmol, 0.20 equiv). The reaction mixture was stirred at room temperature for 24 h, the reaction evolution was verified by ^{1}H NMR ($CDCl_3$) and TLC and then the mixture was concentrated to dryness under reduced pressure. Purification by flash column chromatography using

a CH_2Cl_2/MeOH mixture as eluent afforded the product as an orange solid (0.195 g, 0.155 mmol, yield 77%).

***R*f** (CH_2Cl_2/MeOH 10:1) = 295; **^{1}H-NMR ($CDCl_3$) δ_H, ppm:** 8.54 (1H, s, (*Coum*)O-C-C-C$\underline{H}$-C-(C=O)), 7.78 (2H, d, 2xS-(*Ar*)C-C$\underline{H}$-CH-C-O, $^3J_{H,H}$ = 8.7 Hz), 7.38-7.48 (8H, m, 4xCH$_2$-(*Ar*)C-C$\underline{H}$-CH-C-C(CH$_3$)$_3$, 4xCH$_2$-(*Ar*)C-CH-C$\underline{H}$-C-C(CH$_3$)$_3$), 7.18 (2H, d, 2xS-(*Ar*)C-CH-C$\underline{H}$-C-O, $^3J_{H,H}$ = 8.7 Hz), 7.03 (1H, s, (*Coum*)O-C-C-C$\underline{H}$-C-CH$_2$), 5.10 (2H, d, 2xCH$_3$-(*Ar*)C-CH-C$\underline{H}$-C, $^3J_{H,H}$ = 5.8 Hz), 5.00 (2H, d, 2xCH$_3$-(*Ar*)C-CH-C$\underline{H}$-C, $^3J_{H,H}$ = 5.8 Hz), 4.87 (2H, d, 2xCH$_3$-(*Ar*)C-C$\underline{H}$-CH-C, $^3J_{H,H}$ = 5.8 Hz), 4.65 (2H, d, 2xCH$_3$-(*Ar*)C-C$\underline{H}$-CH-C, $^3J_{H,H}$ = 5.8 Hz), 3.60 (2H, s, S-C$\underline{H_2}$-(*Ar*)C-CH-CH-C-C(CH$_3$)$_3$), 3.42 (2H, s, S-C$\underline{H_2}$-(*Ar*)C-CH-CH-C-C(CH$_3$)$_3$), 3.37 (2H, t, (*Coum*)O-C-C-CH-C-(CH$_2$)$_2$-C$\underline{H_2}$-N, $^3J_{H,H}$ = 5.7 Hz), 3.36 (2H, t, (*Coum*)O-C-C-(CH$_2$)$_2$-C$\underline{H_2}$-N), $^3J_{H,H}$ = 5.6 Hz), 2.89 (2H, t, (*Coum*)O-C-C-CH-C-C$\underline{H_2}$-(CH$_2$)$_2$-N, $^3J_{H,H}$ = 6.3 Hz), 2.78 (2H, t, (*Coum*)O-C-C-C$\underline{H_2}$-(CH$_2$)$_2$-N, $^3J_{H,H}$ = 6.2 Hz), 1.93-2.01 (4H, m, (*Coum*)O-C-C-CH-C-CH$_2$-C$\underline{H_2}$-CH$_2$-N, (*Coum*)O-C-C-CH$_2$-C$\underline{H_2}$-CH$_2$-N), 1.94 (2H, sept, 2x(*Ar*)C-CH-CH-C-C$\underline{H}$(CH$_3$)$_2$, $^3J_{H,H}$ = 6.9 Hz), 1.76 (6H, s, 2xC$\underline{H_3}$-(*Ar*)C-CH-CH-C), 1.36 (9H, s, S-CH$_2$-(*Ar*)C-CH-CH-C-C(C$\underline{H_3}$)$_3$), 1.33 (9H, s, S-CH$_2$-(*Ar*)C-CH-CH-C-C(C$\underline{H_3}$)$_3$), 0.97 (6H, d, (*Ar*)C-CH-CH-C-CH(C$\underline{H_3}$)$_2$, $^3J_{H,H}$ = 6.9 Hz), 0.91 (6H, d, (*Ar*)C-CH-CH-C-CH(C$\underline{H_3}$)$_2$, $^3J_{H,H}$ = 6.9 Hz); **^{13}C-NMR ($CDCl_3$) δ_C, ppm:** 162.5 (1C, S-(*Ar*)C-CH-CH-C-O-($\underline{C}$=O)), 158.8 (1C, (*Coum*)O-C-C-CH-C-($\underline{C}$=O)), 154.0 (1C, (*Coum*)O-$\underline{C}$-C-CH-C-(C=O)), 151.9, 151.8 (2C, 2xS-CH$_2$-(*Ar*)C-CH-CH-$\underline{C}$-C(CH$_3$)$_3$), 151.6 (1C, (*Ar*)$\underline{C}$-N-CH$_2$-CH$_2$), 150.3 (1C, (*Coum*)O-C-C-$\underline{C}$H-C-(C=O)), 149.5 (1C, S-(*Ar*)C-CH-CH-$\underline{C}$-O-C(C=O)), 136.8, 136.7 (2C, 2xS-CH$_2$-(*Ar*)$\underline{C}$-CH-CH-C-C(CH$_3$)$_3$), 134.7 (1C, S-(*Ar*)$\underline{C}$-CH-CH-C-O-(C=O)), 133.5 (2C, 2xS-(*Ar*)C-$\underline{C}$H-CH-C-O-(C=O)), 129.4, 129.2 (4C, 4xS-CH$_2$-(*Ar*)C-$\underline{C}$H-CH-C-C(CH$_3$)$_3$), 127.7 (1C, (*Coum*)O-C-C-$\underline{C}$H-C-(CH$_2$)$_3$-N), 125.7, 125.6 (4C, 4xS-CH$_2$-(*Ar*)C-CH-$\underline{C}$H-C-C(CH$_3$)$_3$), 122.9 (2C, 2xS-(*Ar*)C-CH-C$\underline{H}$-C-O-(C=O))), 119.8 (1C, (*Coum*)O-C-$\underline{C}$-(CH$_2$)$_3$-N), 107.8 (1C, (*Coum*)O-C-C-CH-$\underline{C}$-(C=O)-O), 107.5 (2C, 2xCH$_3$-(*Ar*)C-CH-CH-$\underline{C}$), 105.9 (1C, (*Coum*)O-C-$\underline{C}$-CH-C-(C=O)-O), 105.2 (1C, (*Coum*)O-C-C-CH-$\underline{C}$-(CH$_2$)$_3$-N), 100.5 (2C, 2xCH$_3$-(*Ar*)$\underline{C}$-CH-CH-C), 84.0 (2C, 2xCH$_3$-(*Ar*)C-CH-C$\underline{H}$-C), 83.92 (2C, 2xCH$_3$-(*Ar*)C-CH-$\underline{C}$H-C), 83.89 (2C, 2xCH$_3$-(*Ar*)C-$\underline{C}$H-CH-C), 82.5 (2C, 2xCH$_3$-(*Ar*)C-$\underline{C}$H-CH-C), 50.6 (1C, (*Coum*)O-C-C-CH-C-(CH$_2$)$_2$-$\underline{C}$H$_2$-N), 50.1 (1C, (*Coum*)O-C-C-(CH$_2$)$_2$-$\underline{C}$H$_2$-N), 40.1 (1C, S-$\underline{C}$H$_2$-(*Ar*)C-CH-CH-C-C(CH$_3$)$_3$), 39.6 (1C, S-$\underline{C}$H$_2$-(*Ar*)C-CH-CH-C-C(CH$_3$)$_3$), 34.93 (1C, S-CH$_2$-(*Ar*)C-CH-CH-C-$\underline{C}$(CH$_3$)$_3$), 34.89 (1C, S-CH$_2$-(*Ar*)C-CH-CH-C-$\underline{C}$(CH$_3$)$_3$), 31.56 (3C, S-CH$_2$-(*Ar*)C-CH-CH-C-C($\underline{C}$H$_3$)$_3$), 31.54 (3C, S-CH$_2$-(*Ar*)C-CH-CH-C-C($\underline{C}$H$_3$)$_3$), 31.0 (2C, 2x(*Ar*)CH-CH-C-$\underline{C}$H(CH$_3$)$_2$), 27.5 (1C, (*Coum*)O-C-C-CH-C-$\underline{C}$H$_2$-(CH$_2$)$_2$-N), 23.2 (2C, (*Ar*)CH-CH-C-CH($\underline{C}$H$_3$)$_2$), 22.8 (2C, (*Ar*)CH-CH-C-CH($\underline{C}$H$_3$)$_2$), 21.1 (1C, (*Coum*)O-C-C-CH-C-CH$_2$-$\underline{C}$H$_2$-CH$_2$-N), 20.23 (1C, (*Coum*)O-C-C-CH$_2$-$\underline{C}$H$_2$-CH$_2$-N), 20.19 (1C, (*Coum*)O-C-C-$\underline{C}$H$_2$-(CH$_2$)$_2$-N), 18.3 (2C, 2x$\underline{C}$H$_3$-(*Ar*)C-CH-CH); **HRMS (ESI(+)):** *m/z* calcd for $C_{64}H_{76}NO_4Ru_2S_3^+$: 1222.3018 [M-Cl]$^+$; found: 1222.3010 (the isotopic pattern corresponds well to the calculated one); **elemental analysis** calcd (%) for $C_{64}H_{76}ClNO_4Ru_2S_3\cdot 0.5CH_2Cl_2\cdot CH_3OH$: C 59.08, H 6.13, N 1.05; found: C 59.14, H 6.04, N 0.97.

Synthesis of [(η^6-*p*-MeC$_6$H$_4$Pr^i)$_2$Ru$_2$(μ_2-SCH$_2$C$_6$H$_4$-*p*-CF$_3$)$_2$(μ_2-SC$_6$H$_4$-*p*-OR)]Cl (R = 11-oxo-2,3,6,7-tetrahydro-1*H*,5*H*,11*H*-pyrano[2,3-*f*]pyrido[3,2,1-*ij*]quinoline-10-carboxylate) (12b)

To a solution of **Dye2-CO$_2$H** (0.113 g, 0.397 mmol, 1.3 equiv) in dry CH_2Cl_2 (100 mL) were added successively EDCI (0.076 g, 0.397 mmol, 1.3 equiv), **2b** (0.310 g, 0.306 mmol, 1 equiv), and DMAP (0.011 g, 0.092 mmol, 0.3 equiv). The reaction mixture was stirred at room temperature for 24 h, the reaction evolution was verified by ^{1}H NMR ($CDCl_3$) and TLC and then the mixture was concentrated to dryness under reduced pressure. Purification by flash column chromatography using a CH_2Cl_2/MeOH mixture as eluent afforded **12b** as an orange solid (0.257 g, 0.201 mmol, yield 65%).

***R*f** (CH_2Cl_2/CH_3OH 9.5:0.5) = 0.279; **^{1}H-NMR ($CDCl_3$) δ_H, ppm:** 8.50 (1H, s, (*Coum*)O-C-C-C*H*-C-(C=O)), 7.78 (2H, d, 2xS-(*Ar*)C-C*H*-CH-C-O, $^3J_{H,H}$ = 8.6 Hz), 7.71 (4H, qvart, 4xS-CH_2-(*Ar*)C-CH-C*H*-C-CF_3, $^3J_{H,H}$ = 8.2 Hz, $^3J_{H,F}$ = 8.3 Hz), 7.64 (4H, d, 4xS-CH_2-(*Ar*)C-C*H*-CH-C-CF_3, $^3J_{H,H}$ = 8.2 Hz), 7.16 (2H, d, 2xS-(*Ar*)C-CH-C*H*-C-O, $^3J_{H,H}$ = 8.6 Hz), 6.99 (1H, s, (*Coum*)O-C-C-C*H*-C-CH_2), 5.23 (2H, d, 2xCH_3-(*Ar*)C-CH-C*H*-C, $^3J_{H,H}$ = 5.8 Hz), 5.20 (2H, d, 2xCH_3-(*Ar*)C-CH-C*H*-C, $^3J_{H,H}$ = 5.8 Hz), 5.00 (2H, d, 2xCH_3-(*Ar*)C-C*H*-CH-C, $^3J_{H,H}$ = 5.8 Hz), 4.83 (2H, d, 2xCH_3-(*Ar*)C-C*H*-CH-C, $^3J_{H,H}$ = 5.8 Hz), 3.77 (2H, s, S-C*H_2*-(*Ar*)C-CH-CH-C-CF_3), 3.65 (2H, s, S-C*H_2*-(*Ar*)C-CH-CH-C-CF_3), 3.36 (2H, t, (*Coum*)O-C-C-CH-C-$(CH_2)_2$-C*H_2*-N, $^3J_{H,H}$ = 6.0 Hz), 3.34 (2H, t, (*Coum*)O-C-C-$(CH_2)_2$-C*H_2*-N, $^3J_{H,H}$ = 6.0 Hz), 2.84 (2H, t, (*Coum*)O-C-C-CH-C-C*H_2*-$(CH_2)_2$-N, $^3J_{H,H}$ = 6.4 Hz), 2.75 (2H, t, (*Coum*)O-C-C-C*H_2*-$(CH_2)_2$-N, $^3J_{H,H}$ = 6.2 Hz), 1.93-2.00 (4H, m, (*Coum*)O-C-C-CH-C-CH_2-C*H_2*-CH_2-N, (*Coum*)O-C-C-CH_2-C*H_2*-CH_2-N), 1.87 (2H, sept, 2x(*Ar*)C-CH-CH-C-C*H*$(CH_3)_2$, $^3J_{H,H}$ = 6.9 Hz), 1.74 (6H, s, 2xC*H_3*-(*Ar*)C-CH-CH-C), 0.96 (6H, d, (*Ar*)C-CH-CH-C-CH(C*H_3*)$_2$, $^3J_{H,H}$ = 6.9 Hz), 0.88 (6H, d, (*Ar*)C-CH-CH-C-CH(C*H_3*)$_2$, $^3J_{H,H}$ = 6.9 Hz); **^{13}C-NMR ($CDCl_3$) δ_C, ppm:** 162.4 (1C, S-(*Ar*)C-CH-CH-C-O-(*C*=O)), 158.6 (1C, (*Coum*)O-C-C-CH-C-(*C*=O)), 153.9 (1C, (*Coum*)O-*C*-C-CH-C-(C=O)), 151.6 (1C, S-(*Ar*)C-CH-CH-*C*-O), 150.2 (1C, (*Coum*)O-C-C-*C*H-C-(C=O)), 149.5 (1C, (*Ar*)*C*-N-CH_2-CH_2), 144.2, 144.1 (2C, 2xS-CH_2-(*Ar*)*C*-CH-CH-C-CF_3), 134.3 (1C, S-(*Ar*)*C*-CH-CH-C-O), 133.6 (2C, 2xS-(*Ar*)C-*C*H-CH-C-O), 130.4 (1C, m, S-CH_2-(*Ar*)C-CH-CH-*C*-CF_3), 130.1 (1C, m, S-CH_2-(*Ar*)C-CH-CH-*C*-CF_3), 130.2, 129.9 (4C, 4xS-CH_2-(*Ar*)C-*C*H-CH-C-CF_3), 127.5 (1C, (*Coum*)O-C-C-*C*H-C-$(CH_2)_3$-N), 125.7 (2C, m, 2xS-CH_2-(*Ar*)C-CH-*C*H-C-CF_3, $^3J_{C,F}$ = 4 Hz), 125.5 (2C, m, 2xS-CH_2-(*Ar*)C-CH-*C*H-C-CF_3, $^3J_{C,F}$ = 4 Hz), 124.0 (2C, qvart, 2xS-CH_2-(*Ar*)C-CH-CH-C-*C*F_3, $^1J_{C,F}$ = 272 Hz), 122.8 (2C, 2xS-(*Ar*)C-CH-*C*H-C-O), 119.8 (1C, (*Coum*)O-C-*C*-$(CH_2)_3$-N), 107.8 (2C, 2xCH_3-(*Ar*)C-CH-CH-*C*), 107.7 (1C, (*Coum*)O-C-C-CH-*C*-(C=O)-O), 105.7 (1C, (*Coum*)O-C-*C*-CH-C-(C=O)-O), 104.9 (1C, (*Coum*)O-C-C-CH-*C*-$(CH_2)_3$-N), 100.4 (2C, 2xCH_3-(*Ar*)*C*-CH-CH-C), 84.2 (2C, 2xCH_3-(*Ar*)C-CH-*C*H-C), 84.1 (2C, 2xCH_3-(*Ar*)C-*C*H-CH-C), 83.9 (2C, 2xCH_3-(*Ar*)C-CH-*C*H-C), 82.4 (2C, 2xCH_3-(*Ar*)C-*C*H-CH-C), 50.5 (1C, (*Coum*)O-C-C-CH-C-$(CH_2)_2$-*C*H_2-N), 50.0 (1C, (*Coum*)O-C-C-$(CH_2)_2$-*C*H_2-N), 40.1 (1C, S-*C*H_2-(*Ar*)C-CH-CH-C-CF_3), 39.8 (1C, S-*C*H_2-(*Ar*)C-CH-CH-C-CF_3), 30.9 (2C, 2x(*Ar*)CH-CH-C-*C*H$(CH_3)_2$), 27.4 (1C, (*Coum*)O-C-C-CH-C-*C*H_2-$(CH_2)_2$-N), 23.1 (2C, (*Ar*)CH-CH-C-CH(*C*H_3)$_2$), 22.5 (2C, (*Ar*)CH-CH-C-CH(*C*H_3)$_2$), 21.1 (1C, (*Coum*)O-C-C-CH-C-CH_2-*C*H_2-CH_2-N), 20.1 (2C, (*Coum*)O-C-C-CH_2-*C*H_2-CH_2-N, (*Coum*)O-C-C-*C*H_2-$(CH_2)_2$-N), 18.1 (2C, 2x*C*H_3-(*Ar*)C-CH-CH); **^{19}F-NMR ($CDCl_3$) δ_F, ppm:** -62.30, -62.33 (6F, 2xC*F_3*); **HRMS (ESI(+)):** *m/z* calcd for $C_{58}H_{58}F_6NO_4Ru_2S_3^+$: 1246.1514 $[M-Cl]^+$; found: 1246.1556 (the isotopic pattern corresponds well to the calculated one); **elemental analysis** calcd (%) for $C_{58}H_{58}ClF_6NO_4Ru_2S_3 \cdot CH_3OH$: C 53.98; H 4.76; N 1.07; found C 53.96; H 4.70; N 4.44.

Synthesis of [(η^6-*p*-$MeC_6H_4{}^i Pr)_2Ru_2(SCH_2C_6H_4{}^t Bu)_2(SC_6H_4$-*p*-NHR)]Cl (R = 7-(diethylamino)-2-oxo-2*H*-chromene-3-carboxylate) (13)

To a solution of **Dye1-CO_2H** (0.056 g, 0.202 mmol, 1 equiv) in dry CH_2Cl_2 (100 mL) were added were added HOBt·H_2O (0.082 g, 0.485 mmol, 2.4 equiv) and DIPEA (0.09 mL, 0.505 mmol, 2.5 equiv). After 10 min were added successively EDCI (0.116 g, 0.606 mmol, 3 equiv), **3a** (0.200 g, 0.202 mmol, 1 equiv), and DIPEA (0.09 mL, 0.505 mmol, 2.5 equiv). The reaction mixture was stirred at room temperature for 24 h, the reaction evolution was verified by ^{1}H NMR ($CDCl_3$) and TLC and then the mixture was concentrated to dryness under reduced pressure. Purification by flash column chromatography using a CH_2Cl_2/MeOH mixture as eluent afforded **13** as an orange solid (0.225 g, 0.186 mmol, yield 91%).

***R*f** (CH_2Cl_2/MeOH 10:1) = 0.450; **^{1}H-NMR ($CDCl_3$) δ_H, ppm:** 11.05 (1H, s, N*H*-(C=O), 8.80 (1H, s, (*Coum*)O-C-C-C*H*-C-(C=O)), 7.70-7.75 (4H, m, 2xS-(*Ar*)C-C*H*-CH-C-NH-(C=O), 2xS-(*Ar*)C-CH-C*H*-C-NH-(C=O)), 7.41-7.52 (9H, m, 4xCH_2-(*Ar*)C-C*H*-CH-C-C(CH_3)$_3$, 4xCH_2-(*Ar*)C-CH-C*H*-C-C(CH_3)$_3$, (*Coum*)O-C-C-C*H*-CH-C-N(CH_2-CH_3)$_2$), 6.71 (1H, dd, (*Coum*)O-C-C-CH-C*H*-C-N(CH_2-CH_3)$_2$, $^3J_{H,H}$ = 9.0 Hz, $^4J_{H,H}$ = 2.4 Hz), 6.55 (1H, d, (*Coum*)O-C-C*H*-C-N(CH_2-CH_3)$_2$, $^4J_{H,H}$ = 2.3 Hz), 5.09 (2H, d, 2xCH_3-(*Ar*)C-CH-C*H*-C, $^3J_{H,H}$ = 5.7 Hz), 5.03 (2H, d, 2xCH_3-(*Ar*)C-CH-C*H*-C, $^3J_{H,H}$ = 5.8 Hz), 4.86 (2H, d, 2xCH_3-(*Ar*)C-C*H*-CH-C, $^3J_{H,H}$ = 5.8 Hz), 4.66 (2H, d, 2xCH_3-(*Ar*)C-C*H*-CH-C, $^3J_{H,H}$ = 5.8 Hz), 3.63 (2H, s, S-C*H$_2$*-(*Ar*)C-CH-CH-C-C(CH_3)$_3$), 3.49 (4H, qvart, N(C*H$_2$*-CH_3)$_2$, $^3J_{H,H}$ = 7.2 Hz), 3.44 (2H, s, S-C*H$_2$*-(*Ar*)C-CH-CH-C-C(CH_3)$_3$), 1.96 (2H, sept, 2x(*Ar*)C-CH-CH-C-C*H*(CH_3)$_2$, $^3J_{H,H}$ = 6.9 Hz), 1.77 (6H, s, 2xC*H$_3$*-(*Ar*)C-CH-CH-C), 1.37 (9H, s, S-CH_2-(*Ar*)C-CH-CH-C-C(C*H$_3$*)$_3$), 1.34 (9H, s, S-CH_2-(*Ar*)C-CH-CH-C-C(C*H$_3$*)$_3$), 1.27 (6H, t, N(CH_2-C*H$_3$*)$_2$, $^3J_{H,H}$ = 7.2 Hz), 0.97 (6H, d, (*Ar*)C-CH-CH-C-CH(C*H$_3$*)$_2$, $^3J_{H,H}$ = 6.9 Hz), 0.92 (6H, d, (*Ar*)C-CH-CH-C-CH(C*H$_3$*)$_2$, $^3J_{H,H}$ = 6.9 Hz); **^{13}C-NMR ($CDCl_3$) δ_C, ppm:** 163.3 (1C, (*Coum*)O-C-C-CH-C-(C=O)-NH), 161.5 (1C, (*Coum*)O-C-C-CH-C-(C=O)), 158.0 (1C, (*Coum*)O-C-C-CH-C-(C=O)), 153.2 (1C, (*Ar*)C-N(CH_2-CH_3), 151.9, 151.8 (2C, 2xS-CH_2-(*Ar*)C-CH-CH-C-C(CH_3)$_3$), 148.8 (1C, (*Coum*)O-C-C-CH-C-(C=O)), 139.1 (1C, S-(*Ar*)C-CH-CH-C-NH-(C=O)), 136.81, 136.75 (2C, 2xS-CH_2-(*Ar*)C-CH-CH-C-C(CH_3)$_3$), 133.5 (2C, 2xS-(*Ar*)C-CH-CH-C-NH-(C=O)), 132.6 (1C, S-(*Ar*)C-CH-CH-C-NH-(C=O)), 131.6 (1C, (*Coum*)O-C-C-CH-CH-C-N(CH_2CH_3)$_2$), 129.5, 129.2 (4C, 4xS-CH_2-(*Ar*)C-CH-CH-C-C(CH_3)$_3$), 125.7, 125.6 (4C, 4xS-CH_2-(*Ar*)C-CH-CH-C-C(CH_3)$_3$), 120.4 (2C, 2xS-(*Ar*)C-CH-CH-C-NH-(C=O)), 110.6 (1C, (*Coum*)O-C-C-CH-C-(C=O)), 109.7 (1C, (*Coum*)O-C-C-CH-CH-C-N(CH_2-CH_3), 108.7 (1C, (*Coum*)O-C-C-CH-C-(C=O)), 107.5 (2C, 2xCH_3-(*Ar*)C-CH-CH-C), 100.4 (2C, 2xCH_3-(*Ar*)C-CH-CH-C), 96.8 (1C, (*Coum*)O-C-CH-C-N(CH_2CH_3)$_2$), 84.1 (2C, 2xCH_3-(*Ar*)C-CH-CH-C), 83.9 (2C, 2xCH_3-(*Ar*)C-CH-CH-C), 83.7 (2C, 2xCH_3-(*Ar*)C-CH-CH-C), 82.5 (2C, 2xCH_3-(*Ar*)C-CH-CH-C), 45.4 (2C, N(CH_2-CH_3)$_2$), 40.1 (1C, S-CH_2-(*Ar*)C-CH-CH-C-C(CH_3)$_3$), 39.6 (1C, S-CH_2-(*Ar*)C-CH-CH-C-C(CH_3)$_3$), 34.95 (1C, S-CH_2-(*Ar*)C-CH-CH-C-C(CH_3)$_3$), 34.90 (1C, S-CH_2-(*Ar*)C-CH-CH-C-C(CH_3)$_3$), 31.57 (3C, S-CH_2-(*Ar*)C-CH-CH-C-C(CH_3)$_3$), 31.56 (3C, S-CH_2-(*Ar*)C-CH-CH-C-C(CH_3)$_3$), 31.0 (2C, 2x(*Ar*)CH-CH-C-CH(CH_3)$_2$), 23.2 (2C, (*Ar*)CH-CH-C-CH(CH_3)$_2$), 22.9 (2C, (*Ar*)CH-CH-C-CH(CH_3)$_2$), 18.3 (2C, 2xCH_3-(*Ar*)C-CH-CH), 12.6 (2C, N(CH_2-CH_3)$_2$); **HRMS (ESI(+)):** *m*/*z* calcd for $C_{62}H_{77}N_2O_3Ru_2S_3^+$: 1197.3178 [M-Cl]$^+$; found: 1197.3159 (the isotopic pattern corresponds well to the calculated one); **elemental analysis** calcd (%) for $C_{62}H_{77}ClN_2O_3Ru_2S_3\cdot 0.5CH_2Cl_2\cdot CH_3OH$: C 58.37, H 6.33, N 2.14; found C 58.60, H 6.65, N 2.14.

Synthesis of [(η^6-*p*-MeC$_6$H$_4$^{i}Pr)$_2$Ru$_2$(SCH$_2$C$_6$H$_4$^{t}Bu)$_2$(SC$_6$H$_4$-*p*-NHR)]Cl (R = 11-oxo-2,3,6,7-tetrahydro-1*H*,5*H*,11*H*-pyrano[2,3-*f*]pyrido[3,2,1-*ij*]quinoline-10-carboxylate) (14a)

To a solution of **Dye2-CO$_2$H** (0.058 g, 0.202 mmol, 1 equiv) in dry CH_2Cl_2 (100 mL) were added were added HOBt·H_2O (0.082 g,0.485 mmol, 2.4 equiv) and DIPEA (0.09 mL, 0.505 mmol, 2.5 equiv). After 10 min were added successively EDCI (0.116 g, 0.606 mmol, 3 equiv), **3a** (0.200 g, 0.202 mmol, 1 equiv), and DIPEA (0.09 mL, 0.505 mmol, 2.5 equiv). The reaction mixture was stirred at room temperature for 24 h, the reaction evolution was verified by ^{1}H NMR ($CDCl_3$) and TLC and then the mixture was concentrated to dryness under reduced pressure. Purification by flash column chromatography using a CH_2Cl_2/MeOH mixture as eluent afforded **14a** as an orange solid (0.209 g, 0.167 mmol, yield 82%).

***R*f** (CH_2Cl_2/MeOH 10:1) = 0.400; **^{1}H-NMR ($CDCl_3$) δ_H, ppm:** 11.13 (1H, s, N*H*), 7.69 (1H, s, (*Coum*)O-C-C-C*H*-C-(C=O)), 7.68-7.74 (4H, m, 2xS-(*Ar*)C-C*H*-CH-C-NH, 2xS-(*Ar*)C-CH-C*H*-C-NH), 7.40-7.49 (8H, m, 4xCH_2-(*Ar*)C-C*H*-CH-C-C(CH_3)$_3$, 4xCH_2-(*Ar*)C-CH-C*H*-C-C(CH_3)$_3$), 7.06

(1H, s, (*Coum*)O-C-C-C*H*-C-CH$_2$), 5.07 (2H, d, 2xCH$_3$-(*Ar*)C-CH-C*H*-C, $^3J_{H,H}$ = 5.7 Hz), 5.01 (2H, d, 2xCH$_3$-(*Ar*)C-CH-C*H*-C, $^3J_{H,H}$ = 5.8 Hz), 4.84 (2H, d, 2xCH$_3$-(*Ar*)C-C*H*-CH-C, $^3J_{H,H}$ = 5.8 Hz), 4.65 (2H, d, 2xCH$_3$-(*Ar*)C-C*H*-CH-C, $^3J_{H,H}$ = 5.8 Hz), 3.61 (2H, s, S-C*H$_2$*-(*Ar*)C-CH-CH-C-C(CH$_3$)$_3$), 3.43 (2H, s, S-C*H$_2$*-(*Ar*)C-CH-CH-C-C(CH$_3$)$_3$), 3.34-3.41 (4H, m, (*Coum*)O-C-C-CH-C-(CH$_2$)$_2$-C*H$_2$*-N, (*Coum*)O-C-C-(CH$_2$)$_2$-C*H$_2$*-N), 2.92 (2H, t, (*Coum*)O-C-C-CH-C-C*H$_2$*-(CH$_2$)$_2$-N, $^3J_{H,H}$ = 6.4 Hz), 2.80 (2H, t, (*Coum*)O-C-C-C*H$_2$*-(CH$_2$)$_2$-N, $^3J_{H,H}$ = 6.2 Hz), 1.94-2.04 (4H, m, (*Coum*)O-C-C-CH-C-CH$_2$-C*H$_2$*-CH$_2$-N, (*Coum*)O-C-C-CH$_2$-C*H$_2$*-CH$_2$-N), 1.95 (2H, sept, 2x(*Ar*)C-CH-CH-C-C*H*(CH$_3$)$_2$, $^3J_{H,H}$ = 6.9 Hz), 1.76 (6H, s, 2xC*H$_3$*-(*Ar*)C-CH-CH-C), 1.36 (9H, s, S-CH$_2$-(*Ar*)C-CH-CH-C-C(C*H$_3$*)$_3$), 1.34 (9H, s, S-CH$_2$-(*Ar*)C-CH-CH-C-C(C*H$_3$*)$_3$), 0.96 (6H, d, (*Ar*)C-CH-CH-C-CH(C*H$_3$*)$_2$, $^3J_{H,H}$ = 6.9 Hz), 0.91 (6H, d, (*Ar*)C-CH-CH-C-CH(C*H$_3$*)$_2$, $^3J_{H,H}$ = 6.9 Hz); **^{13}C-NMR (CDCl$_3$) δ_C, ppm:** 163.6 (1C, (*Coum*)O-C-C-CH-C-(*C*=O)-NH), 162.0 (1C, (*Coum*)O-C-C-CH-C-(*C*=O)), 153.0 (1C, (*Coum*)O-*C*-C-CH-C-(C=O)), 151.9, 151.8 (2C, 2xS-CH$_2$-(*Ar*)C-CH-CH-*C*-C(CH$_3$)$_3$), 149.0 (1C, (*Ar*)*C*-N-CH$_2$-CH$_2$), 148.6 (1C, (*Coum*)O-C-C-*C*H-C-(C=O)), 139.3 (1C, S-(*Ar*)C-CH-CH-*C*-NH), 136.8, 136.7 (2C, 2xS-CH$_2$-(*Ar*)*C*-CH-CH-C-C(CH$_3$)$_3$), 133.4 (2C, 2xS-(*Ar*)C-*C*H-CH-C-NH), 132.3 (1C, S-(*Ar*)*C*-CH-CH-C-NH), 129.4, 129.2 (4C, 4xS-CH$_2$-(*Ar*)C-*C*H-CH-C-C(CH$_3$)$_3$), 128.5 (1C, (*Coum*)O-C-C-*C*H-C-(CH$_2$)$_3$-N), 125.7, 125.6 (4C, 4xS-CH$_2$-(*Ar*)C-CH-*C*H-C-C(CH$_3$)$_3$), 120.4 (2C, 2xS-(*Ar*)C-CH-*C*H-C-NH), 120.3 (1C, (*Coum*)O-C-*C*-(CH$_2$)$_3$-N), 108.6 (1C, (*Coum*)O-C-C-CH-*C*-(C=O)-NH), 108.2 (1C, (*Coum*)O-C-*C*-CH-C-(C=O)-NH), 107.5 (2C, 2xCH$_3$-(*Ar*)C-CH-CH-*C*), 105.9 (1C, (*Coum*)O-C-C-CH-*C*-(CH$_2$)$_3$-N), 100.4 (2C, 2xCH$_3$-(*Ar*)*C*-CH-CH-C), 84.0 (2C, 2xCH$_3$-(*Ar*)C-CH-*C*H-C), 83.9 (2C, 2xCH$_3$-(*Ar*)C-*C*H-CH-C), 83.7 (2C, 2xCH$_3$-(*Ar*)C-CH-*C*H-C), 82.4 (2C, 2xCH$_3$-(*Ar*)C-*C*H-CH-C), 50.5 (1C, (*Coum*)O-C-C-CH-C-(CH$_2$)$_2$-*C*H$_2$-N), 50.1 (1C, (*Coum*)O-C-C-(CH$_2$)$_2$-*C*H$_2$-N), 40.1 (1C, S-*C*H$_2$-(*Ar*)C-CH-CH-C-C(CH$_3$)$_3$), 39.5 (1C, S-*C*H$_2$-(*Ar*)C-CH-CH-C-C(CH$_3$)$_3$), 34.92 (1C, S-CH$_2$-(*Ar*)C-CH-CH-C-*C*(CH$_3$)$_3$), 34.88 (1C, S-CH$_2$-(*Ar*)C-CH-CH-C-*C*(CH$_3$)$_3$), 31.55 (3C, S-CH$_2$-(*Ar*)C-CH-CH-C-C(*C*H$_3$)$_3$), 31.54 (3C, S-CH$_2$-(*Ar*)C-CH-CH-C-C(*C*H$_3$)$_3$), 31.0 (2C, 2x(*Ar*)CH-CH-C-*C*H(CH$_3$)$_2$), 27.6 (1C, (*Coum*)O-C-C-CH-C-*C*H$_2$-(CH$_2$)$_2$-N), 23.2 (2C, (*Ar*)CH-CH-C-CH(*C*H$_3$)$_2$), 22.9 (2C, (*Ar*)CH-CH-C-CH(*C*H$_3$)$_2$), 21.2 (1C, (*Coum*)O-C-C-CH-C-CH$_2$-*C*H$_2$-CH$_2$-N), 20.2 (2C, (*Coum*)O-C-C-*C*H$_2$-(CH$_2$)$_2$-N, (*Coum*)O-C-C-CH$_2$-*C*H$_2$-CH$_2$-N), 18.3 (2C, 2x*C*H$_3$-(*Ar*)C-CH-CH); **HRMS (ESI(+)):** *m*/*z* calcd for $C_{64}H_{77}N_2O_3Ru_2S_3^+$: 1221.3178 [M-Cl]$^+$; found: 1221.3192 (the isotopic pattern corresponds well to the calculated one); **elemental analysis** calcd (%) for $C_{64}H_{77}ClN_2O_3Ru_2S_3\cdot 0.5CH_2Cl_2\cdot CH_3OH$: C 59.12, H 6.21, N 2.11; found C 59.29, H 6.18, N 2.19.

Synthesis of [(η^6-*p*-MeC$_6$H$_4$Pr^i)$_2$Ru$_2$(μ_2-SCH$_2$C$_6$H$_4$-*p*-CF$_3$)$_2$(μ_2-SC$_6$H$_4$-*p*-NR)]Cl (R = 11-oxo-2,3,6,7-tetrahydro-1*H*,5*H*,11*H*-pyrano[2,3-*f*]pyrido[3,2,1-*ij*]quinoline-10-carboxylate) (14b)

To a solution of **Dye2-CO$_2$H** (0.095 g, 0.334 mmol, 1 equiv) in dry CH_2Cl_2 (100 mL) were added were added HOBt·H_2O (0.084 g, 0.617 mmol, 2.4 equiv) and DIPEA (0.112 mL, 0.643 mmol, 2.5 equiv). After 10 min were added successively EDCI (0.148 g, 0.771 mmol, 3 equiv), **3b** (0.260 g, 0.257 mmol, 1 equiv), and DIPEA (0.112 mL, 0.643 mmol, 2.5 equiv). The reaction mixture was stirred at room temperature for 24 h, the reaction evolution was verified by ^{1}H NMR (CDCl$_3$) and TLC and then the mixture was concentrated to dryness under reduced pressure. Purification by flash column chromatography using a CH_2Cl_2/MeOH mixture as eluent afforded **14b** as an orange solid (0.269 g, 0.210 mmol, yield 82%).

***R*$_f$** (CH_2Cl_2/CH_3OH 9.5:0.5) = 0.313; **^{1}H-NMR (CDCl$_3$) δ_H, ppm:** 11.13 (1H, s, N*H*), 8.65 (1H, s, (*Coum*)O-C-C-C*H*-C-(C=O)), 7.65-7.77 (12H, m, 2xS-(*Ar*)C-C*H*-CH-C-NH, 2xS-(*Ar*)C-CH-C*H*-C-NH, 4xS-CH$_2$-(*Ar*)C-C*H*-CH-C-CF$_3$, 4xS-CH$_2$-(*Ar*)C-CH-C*H*-C-CF$_3$), 7.04 (1H, s, (*Coum*)O-C-C-C*H*-C-CH$_2$), 5.21 (4H, d, 4xCH$_3$-(*Ar*)C-CH-C*H*-C, $^3J_{H,H}$ = 5.9 Hz), 4.99 (2H, d, 2xCH$_3$-(*Ar*)C-C*H*-

CH-C, $^3J_{H,H}$ = 5.7 Hz), 4.85 (2H, d, 2xCH$_3$-(*Ar*)C-C$\underline{H}$-CH-C, $^3J_{H,H}$ = 5.8 Hz), 3.81 (2H, s, S-C$\underline{H_2}$-(*Ar*)C-CH-CH-C-CF$_3$), 3.66 (2H, s, S-C$\underline{H_2}$-(*Ar*)C-CH-CH-C-CF$_3$), 3.32-3.41 (4H, m, (*Coum*)O-C-C-CH-C-(CH$_2$)$_2$-C$\underline{H_2}$-N, (*Coum*)O-C-C-(CH$_2$)$_2$-C$\underline{H_2}$-N), 2.90 (2H, t, (*Coum*)O-C-C-CH-C-C$\underline{H_2}$-(CH$_2$)$_2$-N, $^3J_{H,H}$ = 6.4 Hz), 2.78 (2H, t, (*Coum*)O-C-C-C$\underline{H_2}$-(CH$_2$)$_2$-N, $^3J_{H,H}$ = 6.2 Hz), 1.94-2.01 (4H, m, (*Coum*)O-C-C-CH-C-CH$_2$-C$\underline{H_2}$-CH$_2$-N, (*Coum*)O-C-C-CH$_2$-C$\underline{H_2}$-CH$_2$-N), 1.90 (2H, sept, 2x(*Ar*)C-CH-CH-C-C$\underline{H}$(CH$_3$)$_2$, $^3J_{H,H}$ = 6.8 Hz), 1.75 (6H, s, 2xC$\underline{H_3}$-(*Ar*)C-CH-CH-C), 0.98 (6H, d, (*Ar*)C-CH-CH-C-CH(C$\underline{H_3}$)$_2$, $^3J_{H,H}$ = 6.8 Hz), 0.90 (6H, d, (*Ar*)C-CH-CH-C-CH(C$\underline{H_3}$)$_2$, $^3J_{H,H}$ = 6.8 Hz); **^{13}C-NMR (CDCl$_3$) δ_C, ppm:** 163.6 (1C, (*Coum*)O-C-C-CH-C-($\underline{C}$=O)-NH), 162.0 (1C, (*Coum*)O-C-C-CH-C-($\underline{C}$=O)), 153.0 (1C, (*Coum*)O-$\underline{C}$-C-CH-C-(C=O)), 149.0 (1C, (*Ar*)$\underline{C}$-N-CH$_2$-CH$_2$), 148.6 (1C, (*Coum*)O-C-C-$\underline{C}$H-C-(C=O)), 144.3 (2C, 2xS-CH$_2$-(*Ar*)$\underline{C}$-CH-CH-C-CF$_3$), 139.4 (1C, S-(*Ar*)C-CH-CH-$\underline{C}$-NH), 133.5 (2C, 2xS-(*Ar*)C-$\underline{C}$H-CH-C-NH), 131.9 (1C, S-(*Ar*)$\underline{C}$-CH-CH-C-NH), 130.5 (1C, m, S-CH$_2$-(*Ar*)C-CH-CH-$\underline{C}$-CF$_3$), 130.2 (1C, m, S-CH$_2$-(*Ar*)C-CH-CH-$\underline{C}$-CF$_3$), 130.4, 129.9 (4C, 4xS-CH$_2$-(*Ar*)C-$\underline{C}$H-CH-C-CF$_3$), 127.4 (1C, (*Coum*)O-C-C-$\underline{C}$H-C-(CH$_2$)$_3$-N), 125.7 (2C, m, 2xS-CH$_2$-(*Ar*)C-CH-$\underline{C}$H-C-CF$_3$, $^3J_{C,F}$ = 4 Hz), 125.6 (2C, m, 2xS-CH$_2$-(*Ar*)C-CH-$\underline{C}$H-C-CF$_3$, $^3J_{C,F}$ = 4 Hz), 124.1 (2C, qvart, 2xS-CH$_2$-(*Ar*)C-CH-CH-C-$\underline{C}$F$_3$, $^1J_{C,F}$ = 272 Hz), 120.4 (2C, 2xS-(*Ar*)C-CH-$\underline{C}$H-C-NH), 120.3 (1C, (*Coum*)O-C-$\underline{C}$-(CH$_2$)$_3$-N), 108.6 (1C, (*Coum*)O-C-C-CH-$\underline{C}$-(C=O)-NH), 108.1 (1C, (*Coum*)O-C-$\underline{C}$-CH-C-(C=O)-NH), 108.0 (2C, 2xCH$_3$-(*Ar*)C-CH-CH-$\underline{C}$), 105.8 (1C, (*Coum*)O-C-C-CH-$\underline{C}$-(CH$_2$)$_3$-N), 100.3 (2C, 2xCH$_3$-(*Ar*)$\underline{C}$-CH-CH-C), 84.4 (2C, 2xCH$_3$-(*Ar*)C-CH-$\underline{C}$H-C), 84.3 (2C, 2xCH$_3$-(*Ar*)C-$\underline{C}$H-CH-C), 83.6 (2C, 2xCH$_3$-(*Ar*)C-CH-$\underline{C}$H-C), 82.3 (2C, 2xCH$_3$-(*Ar*)C-$\underline{C}$H-CH-C), 50.5 (1C, (*Coum*)O-C-C-CH-C-(CH$_2$)$_2$-$\underline{C}$H$_2$-N), 50.0 (1C, (*Coum*)O-C-C-(CH$_2$)$_2$-$\underline{C}$H$_2$-N), 40.1 (1C, S-$\underline{C}$H$_2$-(*Ar*)C-CH-CH-C-CF$_3$), 39.8 (1C, S-$\underline{C}$H$_2$-(*Ar*)C-CH-CH-C-CF$_3$), 31.0 (2C, 2x(*Ar*)CH-CH-C-$\underline{C}$H(CH$_3$)$_2$), 27.6 (1C, (*Coum*)O-C-C-CH-C-$\underline{C}$H$_2$-(CH$_2$)$_2$-N), 23.1 (2C, (*Ar*)CH-CH-C-CH($\underline{C}$H$_3$)$_2$), 22.7 (2C, (*Ar*)CH-CH-C-CH($\underline{C}$H$_3$)$_2$), 21.1 (1C, (*Coum*)O-C-C-CH-C-CH$_2$-$\underline{C}$H$_2$-CH$_2$-N), 20.2 (2C, (*Coum*)O-C-C-$\underline{C}$H$_2$-(CH$_2$)$_2$-N, (*Coum*)O-C-C-CH$_2$-$\underline{C}$H$_2$-CH$_2$-N), 18.2 (2C, 2x$\underline{C}$H$_3$-(*Ar*)C-CH-CH); **^{19}F-NMR (CDCl$_3$) δ_F, ppm:** -62.32, -62.34 (6F, 2xC$\underline{F_3}$); **HRMS (ESI(+)):** *m/z* calcd for $C_{58}H_{59}F_6N_2O_3Ru_2S_3^+$: 1245.1674 [M-Cl]$^+$; found: 1245.1711 (the isotopic pattern corresponds well to the calculated one); **elemental analysis** calcd (%) for $C_{58}H_{59}ClF_6N_2O_3Ru_2S_3\cdot CH_3OH$: C 54.02, H 4.84, N 2.14; found C 53.94, H 4.88, N 5.61.

Synthesis of [(η^6-*p*-MeC$_6$H$_4$^{i}Pr)$_2$Ru$_2$(SCH$_2$C$_6$H$_4$^{t}Bu)$_2$(SC$_6$H$_4$-*p*-CH$_2$CO$_2$NH(CH$_2$)$_3$NHR)]Cl (R = 7-(diethylamino)-2-oxo-2*H*-chromene-3-carboxylate) (15)

To a solution of **4a** (0.250 g, 0.242 mmol, 1 equiv), in dry CH$_2$Cl$_2$ (50 mL) were added successively EDCI (0.139 g, 0.726 mmol, 3 equiv), HOBt·H$_2$O (0.081 g, 0.581 mmol, 2.4 equiv) and DIPEA (0.2 mL, 1.210 mmol, 5 equiv). After 10 min was added **8** (0.230 g, 0.726 mmol, 3 equiv). The reaction mixture was stirred at room temperature for 72 h, the reaction evolution was verified by ^{1}H NMR (CDCl$_3$) and TLC and then the mixture was concentrated to dryness under reduced pressure. Purification by flash column chromatography using a CH$_2$Cl$_2$/MeOH mixture as eluent afforded the product as an orange solid (0.120 g, 0.090 mmol, yield 37%).

***R*f** (CH$_2$Cl$_2$/MeOH 10:1) = 0.537; **^{1}H-NMR (CDCl$_3$) δ_H, ppm:** 8.88 (1H, t br, S-(*Ar*)C-CH-CH-C-CH$_2$-(C=O)-N$\underline{H}$, $^3J_{H,H}$ = 5.9 Hz), 8.65 (1H, s, (*Coum*)O-C-C-C$\underline{H}$-C-(C=O)), 8.51 (1H, t br, NH-(CH$_2$)$_2$-CH$_2$-N$\underline{H}$-(C=O)-C(*Coum*), $^3J_{H,H}$ = 5.7 Hz), 7.63 (2H, d, 2xS-(*Ar*)C-CH-C$\underline{H}$-C-CH$_2$-(C=O)-NH, $^3J_{H,H}$ = 8.2 Hz), 7.51 (2H, d, 2xS-(*Ar*)C-C$\underline{H}$-CH-C-CH$_2$-(C=O)-NH, $^3J_{H,H}$ = 8.2 Hz), 7.38-7.49 (9H, m, 4xCH$_2$-(*Ar*)C-C$\underline{H}$-CH-C-C(CH$_3$)$_3$, 4xCH$_2$-(*Ar*)C-CH-C$\underline{H}$-C-C(CH$_3$)$_3$, (*Coum*)O-C-C-C$\underline{H}$-CH-C-N(CH$_2$-CH$_3$)$_2$), 6.62 (1H, dd, (*Coum*)O-C-C-CH-C$\underline{H}$-C-N(CH$_2$-CH$_3$)$_2$, $^3J_{H,H}$ = 9.0 Hz, $^4J_{H,H}$ = 2.4 Hz), 6.45 (1H, d, (*Coum*)O-C-C$\underline{H}$-C-N(CH$_2$-CH$_3$)$_2$, $^4J_{H,H}$ = 2.3 Hz), 5.02 (2H, d, 2xCH$_3$-(*Ar*)C-CH-C$\underline{H}$-C, $^3J_{H,H}$ = 5.8 Hz), 4.90 (2H, d, 2xCH$_3$-(*Ar*)C-CH-C$\underline{H}$-C, $^3J_{H,H}$ = 5.8 Hz), 4.80 (2H, d,

2xCH$_3$-(*Ar*)C-C*H*-CH-C, $^3J_{H,H}$ = 5.7 Hz), 4.59 (2H, d, 2xCH$_3$-(*Ar*)C-C*H*-CH-C, $^3J_{H,H}$ = 5.7 Hz), 3.72 (2H, s, S-(*Ar*)C-CH-CH-C-C*H*$_2$-(C=O)-NH), 3.57 (2H, s, S-C*H*$_2$-(*Ar*)C-CH-CH-C-C(CH$_3$)$_3$), 3.38-3.48 (2H, m, NH-C*H*$_2$-(CH$_2$)$_2$-NH-(C=O)-C(*Coum*)), 3.42 (4H, qvart, N(C*H*$_2$-CH$_3$)$_2$, $^3J_{H,H}$ = 7.1 Hz), 3.37 (2H, s, S-C*H*$_2$-(*Ar*)C-CH-CH-C-C(CH$_3$)$_3$), 3.31 (2H, qvart, NH-(CH$_2$)$_2$-C*H*$_2$-NH-(C=O)-C(*Coum*), $^3J_{H,H}$ = 6.3 Hz), 1.92 (2H, sept, 2x(*Ar*)C-CH-CH-C-C*H*(CH$_3$)$_2$, $^3J_{H,H}$ = 6.9 Hz), 1.83 (2H, qvin, NH-CH$_2$-C*H*$_2$-CH$_2$-NH, $^3J_{H,H}$ = 6.6 Hz), 1.68 (6H, s, 2xC*H*$_3$-(*Ar*)C-CH-CH-C), 1.35 (9H, s, S-CH$_2$-(*Ar*)C-CH-CH-C-C(C*H*$_3$)$_3$), 1.32 (9H, s, S-CH$_2$-(*Ar*)C-CH-CH-C-C(C*H*$_3$)$_3$), 1.21 (6H, t, N(CH$_2$-C*H*$_3$)$_2$, $^3J_{H,H}$ = 7.1 Hz), 0.94 (6H, d, (*Ar*)C-CH-CH-C-CH(C*H*$_3$)$_2$, $^3J_{H,H}$ = 6.9 Hz), 0.89 (6H, d, (*Ar*)C-CH-CH-C-CH(C*H*$_3$)$_2$, $^3J_{H,H}$ = 6.9 Hz); **^{13}C-NMR (CDCl$_3$) δ_C, ppm:** 171.4 (1C, S-(*Ar*)C-CH-CH-C-CH$_2$-(*C*=O)-NH), 163.6 (1C, (*Coum*)O-C-C-CH-C-(*C*=O)-NH), 162.6 (1C, (*Coum*)O-C-C-CH-C-(*C*=O)-O), 157.7 (1C, (*Coum*)O-*C*-C-CH-C-(C=O)), 152.6 (1C, (*Ar*)*C*-N(CH$_2$-CH$_3$), 151.94, 151.87 (2C, 2xS-CH$_2$-(*Ar*)C-CH-CH-*C*-C(CH$_3$)$_3$), 148.0 (1C, (*Coum*)O-C-C-*C*H-C-(C=O)), 138.6 (1C, S-(*Ar*)C-CH-CH-*C*-CH$_2$-(C=O)-NH), 136.8, 136.6 (2C, 2xS-CH$_2$-(*Ar*)*C*-CH-CH-C-C(CH$_3$)$_3$), 135.0 (1C, S-(*Ar*)*C*-CH-CH-C-CH$_2$-(C=O)-NH), 132.4 (2C, 2xS-(*Ar*)C-CH-*C*H-C-CH$_2$-(C=O)-NH), 131.2 (1C, (*Coum*)O-C-C-*C*H-CH-C-N(CH$_2$-CH$_3$)$_2$), 130.5 (2C, 2xS-(*Ar*)C-*C*H-CH-C-CH$_2$-(C=O)-NH), 129.4, 129.1 (4C, 4xS-CH$_2$-(*Ar*)C-*C*H-CH-C-C(CH$_3$)$_3$), 125.7, 125.6 (4C, 4xS-CH$_2$-(*Ar*)C-CH-*C*H-C-C(CH$_3$)$_3$), 110.6 (1C, (*Coum*)O-C-*C*-CH-C-(C=O)), 110.0 (1C, (*Coum*)O-C-C-CH-*C*H-C-N(CH$_2$-CH$_3$), 108.5 (1C, (*Coum*)O-C-C-CH-*C*-(C=O)), 107.4 (2C, 2xCH$_3$-(*Ar*)C-CH-CH-*C*), 100.5 (2C, 2xCH$_3$-(*Ar*)*C*-CH-CH-C), 96.7 (1C, (*Coum*)O-C-*C*H-C-N(CH$_2$CH$_3$)$_2$), 84.0 (2C, 2xCH$_3$-(*Ar*)C-CH-*C*H-C), 83.76 (2C, 2xCH$_3$-(*Ar*)C-CH-*C*H-C), 83.72 (2C, 2xCH$_3$-(*Ar*)C-*C*H-C*H*-C), 82.4 (2C, 2xCH$_3$-(*Ar*)C-*C*H-CH-C), 45.2 (2C, N(*C*H$_2$-CH$_3$)$_2$), 43.3 (1C, S-(*Ar*)C-CH-CH-C-*C*H$_2$-(C=O)-NH), 40.0 (1C, S-*C*H$_2$-(*Ar*)C-CH-CH-C-C(CH$_3$)$_3$), 39.4 (1C, S-*C*H$_2$-(*Ar*)C-CH-CH-C-C(CH$_3$)$_3$), 37.2 (1C, NH-*C*H$_2$-(CH$_2$)$_2$-NH-(C=O)-C(*Coum*)), 36.8 (1C, NH-(CH$_2$)$_2$-*C*H$_2$-NH-(C=O)-C(*Coum*)), 34.92 (1C, S-CH$_2$-(*Ar*)C-CH-CH-C-*C*(CH$_3$)$_3$), 34.88 (1C, S-CH$_2$-(*Ar*)C-CH-CH-C-*C*(CH$_3$)$_3$), 31.55 (3C, S-CH$_2$-(*Ar*)C-CH-CH-C-C(*C*H$_3$)$_3$), 31.53 (3C, S-CH$_2$-(*Ar*)C-CH-CH-C-C(*C*H$_3$)$_3$), 31.0 (2C, 2x(*Ar*)CH-CH-C-*C*H(CH$_3$)$_2$), 29.8 (1C, NH-CH$_2$-*C*H$_2$-CH$_2$-NH-(C=O)-C(*Coum*)), 23.2 (2C, (*Ar*)CH-CH-C-CH(*C*H$_3$)$_2$), 22.8 (2C, (*Ar*)CH-CH-C-CH(*C*H$_3$)$_2$), 18.2 (2C, 2x*C*H$_3$-(*Ar*)C-CH-CH), 12.6 (2C, N(CH$_2$-*C*H$_3$)$_2$); **HRMS (ESI(+)):** *m/z* calcd for $C_{67}H_{86}N3O_4Ru_2S_3^+$: 1296.3862 [M-Cl]$^+$; found: 1296.3842 (the isotopic pattern corresponds well to the calculated one); **elemental analysis** calcd (%) for $C_{67}H_{86}ClN_3O_4Ru_2S_3\cdot 2CH_3OH$: C 59.40, H 6.79, N 3.01; found C 59.45, H 7.28, N 3.01.

Synthesis of [(η^6-*p*-MeC$_6$H$_4$^{i}Pr)$_2$Ru$_2$(SCH$_2$C$_6$H$_4$^{t}Bu)$_2$(SC$_6$H$_4$-*p*-CH$_2$CO$_2$NH(CH$_2$)$_4$NHR)]Cl (R = 7-(diethylamino)-2-oxo-2*H*-chromene-3-carboxylate) (16a)

To a solution of **4a** (0.250 g, 0.242 mmol, 1 equiv), in dry CH_2Cl_2 (50 mL) were added successively EDCI (0.139 g, 0.726 mmol, 3 equiv), HOBt·H_2O (0.081 g, 0.581 mmol, 2.4 equiv) and DIPEA (0.2 mL, 1.210 mmol, 5 equiv). After 10 min was added **9** (0.241 g, 0.726 mmol, 3 equiv). The reaction mixture was stirred at room temperature for 72 h, the reaction evolution was verified by ^{1}H NMR ($CDCl_3$) and TLC and then the mixture was concentrated to dryness under reduced pressure. Purification by flash column chromatography using a CH_2Cl_2/MeOH mixture as eluent afforded the product as an orange solid (0.143 g, 0.106 mmol, yield 44%).

***R*$_f$** (CH_2Cl_2/MeOH 10:1) = 0.500; **^{1}H-NMR (CDCl$_3$) δ_H, ppm:** 8.75 (1H, t br, S-(*Ar*)C-CH-CH-C-CH$_2$-(C=O)-N*H*, $^3J_{H,H}$ = 5.6 Hz), 8.67 (1H, s, (*Coum*)O-C-C-C*H*-C-(C=O)), 8.63 (1H, t br, (*Coum*)O-C-C-CH-C-(C=O)-N*H*, $^3J_{H,H}$ = 6.3 Hz), 7.61 (2H, d, 2xS-(*Ar*)C-CH-C*H*-C-CH$_2$-(C=O)-NH, $^3J_{H,H}$ = 8.2 Hz), 7.54 (2H, d, 2xS-(*Ar*)C-C*H*-CH-C-CH$_2$-(C=O)-NH, $^3J_{H,H}$ = 8.2 Hz), 7.38-7.48 (9H,

m, 4xCH$_2$-(*Ar*)C-C*H*-CH-C-C(CH$_3$)$_3$, 4xCH$_2$-(*Ar*)C-CH-C*H*-C-C(CH$_3$)$_3$, (*Coum*)O-C-C-C*H*-CH-C-N(CH$_2$-CH$_3$)$_2$), 6.61 (1H, dd, (*Coum*)O-C-C-CH-C*H*-C-N(CH$_2$-CH$_3$)$_2$, $^3J_{H,H}$ = 9.0 Hz, $^4J_{H,H}$ = 2.4 Hz), 6.44 (1H, d, (*Coum*)O-C-C*H*-C-N(CH$_2$-CH$_3$)$_2$, $^4J_{H,H}$ = 2.1 Hz), 5.01 (2H, d, 2xCH$_3$-(*Ar*)C-CH-C*H*-C, $^3J_{H,H}$ = 5.7 Hz), 4.89 (2H, d, 2xCH$_3$-(*Ar*)C-CH-C*H*-C, $^3J_{H,H}$ = 5.8 Hz), 4.80 (2H, d, 2xCH$_3$-(*Ar*)C-C*H*-CH-C, $^3J_{H,H}$ = 5.7 Hz), 4.58 (2H, d, 2xCH$_3$-(*Ar*)C-C*H*-CH-C, $^3J_{H,H}$ = 5.8 Hz), 3.75 (2H, s, S-(*Ar*)C-CH-CH-C-C*H$_2$*-(C=O)-NH), 3.56 (2H, s, S-C*H$_2$*-(*Ar*)C-CH-CH-C-C(CH$_3$)$_3$), 3.42 (6H, qvart, N(C*H$_2$*-CH$_3$)$_2$, NH-(CH$_2$)$_3$-C*H$_2$*-NH-(C=O)-C(*Coum*), $^3J_{H,H}$ = 7.0 Hz), 3.36 (2H, s, S-C*H$_2$*-(*Ar*)C-CH-CH-C-C(CH$_3$)$_3$), 3.28 (2H, qvart, NH-C*H$_2$*-(CH$_2$)$_3$-NH-(C=O)-C(*Coum*), $^3J_{H,H}$ = 5.7 Hz), 1.92 (2H, sept, 2x(*Ar*)C-CH-CH-C-C*H*(CH$_3$)$_2$, $^3J_{H,H}$ = 6.9 Hz), 1.68 (6H, s, 2xC*H$_3$*-(*Ar*)C-CH-CH-C), 1.59-1.72 (4H, m, NH-CH$_2$-C*H$_2$*-(CH$_2$)$_2$-NH-(C=O)-C(*Coum*), NH-(CH$_2$)$_2$-C*H$_2$*-CH$_2$-NH-(C=O)-C(*Coum*), 1.36 (9H, s, S-CH$_2$-(*Ar*)C-CH-CH-C-C(C*H$_3$*)$_3$), 1.32 (9H, s, S-CH$_2$-(*Ar*)C-CH-CH-C-C(C*H$_3$*)$_3$), 1.20 (6H, t, N(CH$_2$-C*H$_3$*)$_2$, $^3J_{H,H}$ = 7.1 Hz), 0.94 (6H, d, (*Ar*)C-CH-CH-C-CH(C*H$_3$*)$_2$, $^3J_{H,H}$ = 6.9 Hz), 0.89 (6H, d, (*Ar*)C-CH-CH-C-CH(C*H$_3$*)$_2$, $^3J_{H,H}$ = 6.9 Hz); **^{13}C-NMR (CDCl$_3$) δ_C, ppm:** 171.4 (1C, S-(*Ar*)C-CH-CH-C-CH$_2$-($\underline{C}$=O)-NH), 163.1 (1C, (*Coum*)O-C-C-CH-C-($\underline{C}$=O)-NH), 162.7 (1C, (*Coum*)O-C-C-CH-C-($\underline{C}$=O)-O), 157.7 (1C, (*Coum*)O-$\underline{C}$-C-CH-C-(C=O)), 152.5 (1C, (*Ar*)$\underline{C}$-N(CH$_2$-CH$_3$), 151.93, 151.88 (2C, 2xS-CH$_2$-(*Ar*)C-CH-CH-$\underline{C}$-C(CH$_3$)$_3$), 148.0 (1C, (*Coum*)O-C-C-$\underline{C}$H-C-(C=O)), 138.6 (1C, S-(*Ar*)C-CH-CH-$\underline{C}$-CH$_2$-(C=O)-NH), 136.8, 136.6 (2C, 2xS-CH$_2$-(*Ar*)$\underline{C}$-CH-CH-C-C(CH$_3$)$_3$), 134.7 (1C, S-(*Ar*)$\underline{C}$-CH-CH-C-CH$_2$-(C=O)-NH), 132.3 (2C, 2xS-(*Ar*)C-CH-$\underline{C}$H-C-CH$_2$-(C=O)-NH), 131.2 (1C, (*Coum*)O-C-C-$\underline{C}$H-CH-C-N(CH$_2$-CH$_3$)$_2$), 130.6 (2C, 2xS-(*Ar*)C-$\underline{C}$H-CH-C-CH$_2$-(C=O)-NH), 129.3, 129.1 (4C, 4xS-CH$_2$-(*Ar*)C-$\underline{C}$H-CH-C-C(CH$_3$)$_3$), 125.7, 125.6 (4C, 4xS-CH$_2$-(*Ar*)C-CH-$\underline{C}$H-C-C(CH$_3$)$_3$), 110.6 (1C, (*Coum*)O-C-C-CH-$\underline{C}$-(C=O)), 110.0 (1C, (*Coum*)O-C-C-CH-$\underline{C}$H-C-N(CH$_2$CH$_3$), 108.5 (1C, (*Coum*)O-C-$\underline{C}$-CH-C-(C=O)), 107.4 (2C, 2xCH$_3$-(*Ar*)C-CH-CH-$\underline{C}$), 100.5 (2C, 2xCH$_3$-(*Ar*)$\underline{C}$-CH-CH-C), 96.6 (1C, (*Coum*)O-C-$\underline{C}$H-C-N(CH$_2$-CH$_3$)$_2$), 83.9 (2C, 2xCH$_3$-(*Ar*)C-CH-$\underline{C}$H-C), 83.8 (2C, 2xCH$_3$-(*Ar*)C-$\underline{C}$H-CH-C), 83.7 (2C, 2xCH$_3$-(*Ar*)C-CH-$\underline{C}$H-C), 82.4 (2C, 2xCH$_3$-(*Ar*)C-$\underline{C}$H-CH-C), 45.2 (2C, N($\underline{C}$H$_2$-CH$_3$)$_2$), 43.1 (1C, S-(*Ar*)C-CH-CH-C-$\underline{C}$H$_2$-(C=O)-NH), 40.0 (1C, S-$\underline{C}$H$_2$-(*Ar*)C-CH-CH-C-C(CH$_3$)$_3$), 39.6 (1C, NH-$\underline{C}$H$_2$-(CH$_2$)$_3$-NH-(C=O)-C(*Coum*)), 39.34 (1C, S-$\underline{C}$H$_2$-(*Ar*)C-CH-CH-C-C(CH$_3$)$_3$), 39.27 (1C, NH-(CH$_2$)$_3$-$\underline{C}$H$_2$-NH-(C=O)-C(*Coum*)), 34.90 (1C, S-CH$_2$-(*Ar*)C-CH-CH-C-$\underline{C}$(CH$_3$)$_3$), 34.87 (1C, S-CH$_2$-(*Ar*)C-CH-CH-C-$\underline{C}$(CH$_3$)$_3$), 31.53 (3C, S-CH$_2$-(*Ar*)C-CH-CH-C-C($\underline{C}$H$_3$)$_3$), 31.52 (3C, S-CH$_2$-(*Ar*)C-CH-CH-C-C($\underline{C}$H$_3$)$_3$), 31.0 (2C, 2x(*Ar*)CH-CH-C-$\underline{C}$H(CH$_3$)$_2$), 27.3 (1C, NH-(CH$_2$)$_2$-$\underline{C}$H$_2$-CH$_2$-NH-(C=O)-C(*Coum*)), 27.0 (1C, NH-CH$_2$-$\underline{C}$H$_2$-(CH$_2$)$_2$-NH-(C=O)-C(*Coum*)), 23.1 (2C, (*Ar*)CH-CH-C-CH($\underline{C}$H$_3$)$_2$), 22.8 (2C, (*Ar*)CH-CH-C-CH($\underline{C}$H$_3$)$_2$), 18.1 (2C, 2x$\underline{C}$H$_3$-(*Ar*)C-CH-CH), 12.5 (2C, N(CH$_2$-$\underline{C}$H$_3$)$_2$); **HRMS (ESI(+)):** *m*/*z* calcd for $C_{67}H_{86}N3O_4Ru_2S_3^+$: 1310.4018 [M-Cl]$^+$; found: 1310.3981 (the isotopic pattern corresponds well to the calculated pattern); **elemental analysis** calcd (%) for $C_{68}H_{88}ClN_3O_4Ru_2S_3\cdot 2CH_3OH$: C 59.66, H 6.87, N 2.98, found C 59.63, H 7.57, N 2.96.

Synthesis of [(η^6-*p*-MeC$_6$H$_4$^{i}Pr)$_2$Ru$_2$(SCH$_2$C$_6$H$_4$^{t}Bu)$_2$(SC$_6$H$_4$-*p*-CH$_2$CO$_2$NH(CH$_2$)$_4$NHR)]Cl (R = 7-(diethylamino)-2-oxo-2*H*-chromene-3-carboxylate) (16b)

To a solution of **4b** (0.150 g, 0.142 mmol, 1 equiv), in dry CH_2Cl_2 (50 mL) were added were added HOBt·H_2O (0.060 g, 0.355 mmol, 2.5 equiv) and DIPEA (0.062 mL, 0.355 mmol, 2.5 equiv). After 10 min were added successively EDCI (0.081 g, 0.426 mmol, 3 equiv), **9** (0.094 g, 0.284 mmol, 2 equiv), and DIPEA (0.062 mL, 0.355 mmol, 2.5 equiv). The reaction mixture was stirred at room temperature for 24 h, the reaction evolution was verified by ^{1}H NMR ($CDCl_3$) and TLC and then the mixture was concentrated to dryness under reduced pressure. Purification by flash column chromatography using a CH_2Cl_2/MeOH mixture as eluent afforded **16b** as an orange-yellow solid (0.072 g, 0.052 mmol, yield 37 %).

***R*f** (CH_2Cl_2/CH_3OH 10:1) = 0.433; **^{1}H-NMR ($CDCl_3$) δ_H, ppm:** 8.77 (1H, t br, S-(*Ar*)C-CH-CH-C-CH_2-(C=O)-N*H*, $^3J_{H,H}$ = 5.6 Hz), 8.66 (1H, s, (*Coum*)O-C-C-C*H*-C-(C=O)), 8.16-8.25 (1H, m, (*Coum*)O-C-C-CH-C-(C=O)-N*H*), 7.66-7.76 (8H, m, 4xCH_2-(*Ar*)C-C*H*-CH-C-CF_3, 4xCH_2-(*Ar*)C-CH-C*H*-C-CF_3), 7.65 (2H, d, 2xS-(*Ar*)C-CH-C*H*-C-CH_2-(C=O), $^3J_{H,H}$ = 7.4 Hz), 7.53 (2H, d, 2xS-(*Ar*)C-C*H*-CH-C-CH_2-(C=O), $^3J_{H,H}$ = 7.4 Hz), 7.40 (1H, d, (*Coum*)O-C-C-C*H*-CH-C-N(CH_2-CH_3)$_2$, $^3J_{H,H}$ = 8.9 Hz), 6.62 (1H, dd, (*Coum*)O-C-C-CH-C*H*-C-N(CH_2-CH_3)$_2$, $^3J_{H,H}$ = 8.9 Hz, $^4J_{H,H}$ = 2.0 Hz), 6.45 (1H, d, (*Coum*)O-C-C*H*-C-N(CH_2-CH_3)$_2$, $^4J_{H,H}$ = 1.8 Hz), 5.13 (2H, d, 2xCH_3-(*Ar*)C-CH-C*H*-C, $^3J_{H,H}$ = 4.6 Hz), 5.05 (2H, d, 2xCH_3-(*Ar*)C-CH-C*H*-C, $^3J_{H,H}$ = 5.0 Hz), 4.93 (2H, d, 2xCH_3-(*Ar*)C-C*H*-CH-C, $^3J_{H,H}$ = 4.6 Hz), 4.79 (2H, d, 2xCH_3-(*Ar*)C-C*H*-CH-C, $^3J_{H,H}$ = 4.9 Hz), 3.73 (2H, s, S-C*H$_2$*-(*Ar*)C-CH-CH-C-CF_3), 3.71 (2H, s, S-(*Ar*)C-CH-CH-C-C*H$_2$*-(C=O)-NH), 3.63 (2H, s, S-C*H$_2$*-(*Ar*)C-CH-CH-C-CF_3), 3.43 (6H, qvart, N(C*H$_2$*-CH_3)$_2$, NH-(CH_2)$_3$-C*H$_2$*-NH-(C=O)-C(*Coum*), $^3J_{H,H}$ = 7.1 Hz), 3.24-3.31 (2H, m, NH-C*H$_2$*-(CH_2)$_3$-NH-(C=O)-C(*Coum*)), 1.88 (2H, sept, 2x(*Ar*)C-CH-CH-C-C*H*(CH_3)$_2$, $^3J_{H,H}$ = 6.7 Hz), 1.70 (6H, s, 2xC*H$_3$*-(*Ar*)C-CH-CH-C), 1.62-1.67 (4H, m, NH-CH_2-C*H$_2$*-(CH_2)$_2$-NH-(C=O)-C(*Coum*), NH-(CH_2)$_2$-C*H$_2$*-CH_2-NH-(C=O)-C(*Coum*)), 1.21 (6H, t, N(CH_2-C*H$_3$*)$_2$, $^3J_{H,H}$ = 7.1 Hz), 0.98 (6H, d, (*Ar*)C-CH-CH-C-CH(C*H$_3$*)$_2$, $^3J_{H,H}$ = 6.7 Hz), 0.91 (6H, d, (*Ar*)C-CH-CH-C-CH(C*H$_3$*)$_2$, $^3J_{H,H}$ = 6.7 Hz); **^{13}C-NMR ($CDCl_3$) δ_C, ppm:** 171.2 (1C, S-(*Ar*)C-CH-CH-C-CH_2-(*C*=O)-NH), 163.3 (1C, (*Coum*)O-C-C-CH-C-(*C*=O)-NH), 162.8 (1C, (*Coum*)O-C-C-CH-C-(*C*=O)-O), 157.7 (1C, (*Coum*)O-*C*-C-CH-C-(C=O)), 152.6 (1C, (*Ar*)*C*-N(CH_2-CH_3), 148.1 (1C, (*Coum*)O-C-C-*C*H-C-(C=O)), 144.0, 143.9 (2C, 2xS-CH_2-(*Ar*)*C*-CH-CH-C-CF_3), 138.6 (1C, S-(*Ar*)C-CH-CH-*C*-CH_2-(C=O)-NH), 134.5 (1C, S-(*Ar*)*C*-CH-CH-C-CH_2-(C=O)-NH), 132.6 (2C, 2xS-(*Ar*)C-CH-*C*H-C-CH_2-(C=O)-NH), 131.2 (1C, (*Coum*)O-C-C-*C*H-CH-C-N(CH_2-CH_3)$_2$), 130.6 (2C, 2xS-(*Ar*)C-*C*H-CH-C-CH_2-(C=O)-NH), 130.8 (1C, m, S-CH_2-(*Ar*)C-CH-CH-*C*-CF_3), 130.4 (1C, m, S-CH_2-(*Ar*)C-CH-CH-*C*-CF_3), 130.1, 129.8 (4C, 4xS-CH_2-(*Ar*)C-*C*H-CH-C-CF_3), 125.7-126.0 (4C, m, 4xS-CH_2-(*Ar*)C-CH-*C*H-C-CF_3, $^3J_{C,F}$ = 4 Hz), 124.0 (2C, qvart, 2xS-CH_2-(*Ar*)C-CH-CH-C-*C*F_3, $^1J_{C,F}$ = 272 Hz), 110.6 (1C, (*Coum*)O-C-C-CH-*C*-(C=O)), 110.1 (1C, (*Coum*)O-C-C-CH-*C*H-C-N(CH_2-CH_3), 108.5 (1C, (*Coum*)O-C-*C*-CH-C-(C=O)), 107.9 (2C, 2xCH_3-(*Ar*)C-CH-CH-*C*), 100.6 (2C, 2xCH_3-(*Ar*)*C*-CH-CH-C), 96.7 (1C, (*Coum*)O-C-*C*H-C-N(CH_2-CH_3)$_2$), 84.3 (2C, 2xCH_3-(*Ar*)C-CH-*C*H-C), 84.1 (2C, 2xCH_3-(*Ar*)C-*C*H-CH-C), 83.7 (2C, 2xCH_3-(*Ar*)C-CH-*C*H-C), 82.3 (2C, 2xCH_3-(*Ar*)C-*C*H-CH-C), 45.2 (2C, N(*C*H_2-CH_3)$_2$), 43.2 (1C, S-(*Ar*)C-CH-CH-C-*C*H_2-(C=O)-NH), 40.0 (1C, S-*C*H_2-(*Ar*)C-CH-CH-C-CF_3), 39.7 (1C, S-*C*H_2-(*Ar*)C-CH-CH-C-CF_3), 39.5 (1C, NH-(CH_2)$_3$-*C*H_2-NH-(C=O)-C(*Coum*)), 39.4 (1C, NH-*C*H_2-(CH_2)$_3$-NH-(C=O)-C(*Coum*)), 31.1 (2C, 2x(*Ar*)CH-CH-C-*C*H(CH_3)$_2$), 27.4 (1C, NH-(CH_2)$_2$-*C*H_2-CH_2-NH-(C=O)-C(*Coum*)), 26.9 (1C, NH-CH_2-*C*H_2-(CH_2)$_2$-NH-(C=O)-C(*Coum*)), 23.2 (2C, (*Ar*)CH-CH-C-CH(*C*H_3)$_2$), 22.7 (2C, (*Ar*)CH-CH-C-CH(*C*H_3)$_2$), 18.1 (2C, 2x*C*H_3-(*Ar*)C-CH-CH), 12.6 (2C, N(CH_2-*C*H_3)$_2$); **^{19}F-NMR ($CDCl_3$) δ_F, ppm:** -62.33, -62.39 (6F, 2xC*F$_3$*); **HRMS (ESI(+)):** *m/z* calcd for $C_{62}H_{70}F_6N_3O_4Ru_2S_3^+$: 1334.2514 [M-Cl]$^+$, and for $C_{62}H_{71}F_6N_3O_4Ru_2S_3^{+2}$: 667.6294 [M-Cl+H]$^{+2}$; found: 1334.2494, 667.6280 (the isotopic pattern corresponds well to the calculated one); **elemental analysis** calcd (%) for $C_{62}H_{70}ClF_6N_3O_4Ru_2S_3 \cdot 2C_6H_{14}$: C 57.66, H 6.41, N 2.73; found C 57.66, H 6.44, N 6.37.

Synthesis of [(η^6-*p*-MeC$_6$H$_4$^{i}Pr)$_2$Ru$_2$(SCH$_2$C$_6$H$_4$^{t}Bu)$_2$(SC$_6$H$_4$-*p*-CH$_2$CO$_2$NH(CH$_2$)$_4$NHR)]Cl (R = 11-oxo-2,3,6,7-tetrahydro-1*H*,5*H*,11*H*-pyrano[2,3-*f*]pyrido[3,2,1-*ij*]quinoline-10-carboxylate) (17a)

To a solution of **4a** (0.250 g, 0.242 mmol, 1 equiv), in dry CH_2Cl_2 (50 mL) were added successively EDCI (0.139 g, 0.726 mmol, 3 equiv), HOBt·H_2O (0.081 g, 0.581 mmol, 2.4 equiv) and DIPEA (0.2 mL, 1.210 mmol, 5 equiv). After 10 min was added **10** (0.258 g, 0.726 mmol, 3 equiv). The

reaction mixture was stirred at room temperature for 72 h, the reaction evolution was verified by ^{1}H NMR ($CDCl_3$) and TLC and then the mixture was concentrated to dryness under reduced pressure. Purification by flash column chromatography using a CH_2Cl_2/MeOH mixture as eluent afforded the product as an orange solid (0.230 g, 0.168 mmol, yield 69%).

***R*f** (CH_2Cl_2/MeOH 10:1) = 0.491; **^{1}H-NMR ($CDCl_3$) δ*H*, ppm:** 8.83 (1H, t br, S-(*Ar*)C-CH-CH-C-CH_2-(C=O)-N*H*, $^{3}J_{H,H}$ = 5.8 Hz), 8.62-8.70 (1H, m br, O-C-C-CH-C-(C=O)-N*H*), 8.59 (1H, s, (*Coum*)O-C-C-C*H*-C-(C=O)), 7.62 (2H, d, 2xS-(*Ar*)C-CH-C*H*-C-CH_2-(C=O)-NH, $^{3}J_{H,H}$ = 8.2 Hz), 7.57 (2H, d, 2xS-(*Ar*)C-C*H*-CH-C-CH_2-(C=O)-NH, $^{3}J_{H,H}$ = 8.2 Hz), 7.38-7.50 (8H, m, 4xCH_2-(*Ar*)C-C*H*-CH-C-C$(CH_3)_3$, 4xCH_2-(*Ar*)C-CH-C*H*-C-C$(CH_3)_3$), 6.97 (1H, s, (*Coum*)O-C-C-C*H*-C-CH_2), 5.02 (2H, d, 2xCH_3-(*Ar*)C-CH-C*H*-C, $^{3}J_{H,H}$ = 5.7 Hz), 4.90 (2H, d, 2xCH_3-(*Ar*)C-CH-C*H*-C, $^{3}J_{H,H}$ = 5.8 Hz), 4.80 (2H, d, 2xCH_3-(*Ar*)C-C*H*-CH-C, $^{3}J_{H,H}$ = 5.7 Hz), 4.59 (2H, d, 2xCH_3-(*Ar*)C-C*H*-CH-C, $^{3}J_{H,H}$ = 5.8 Hz), 3.76 (2H, s, S-(*Ar*)C-CH-CH-C-C*H_2*-(C=O)-NH), 3.57 (2H, s, S-C*H_2*-(*Ar*)C-CH-CH-C-C$(CH_3)_3$), 3.40-3.45 (2H, qvart br, NH-$(CH_2)_3$-C*H_2*-NH-(C=O)-C(*Coum*), $^{3}J_{H,H}$ = 5.4 Hz), 3.37 (2H, s, S-C*H_2*-(*Ar*)C-CH-CH-C-C$(CH_3)_3$), 3.28-3.32 (6H, m, (*Coum*)O-C-C-CH-C-$(CH_2)_2$-C*H_2*-N, (*Coum*)O-C-C-$(CH_2)_2$-C*H_2*-N, NH-C*H_2*-$(CH_2)_3$-NH-(C=O)-C(*Coum*), $^{3}J_{H,H}$ = 5.6 Hz), 2.84 (2H, t, (*Coum*)O-C-C-CH-C-C*H_2*-$(CH_2)_2$-N, $^{3}J_{H,H}$ = 6.4 Hz), 2.74 (2H, t, (*Coum*)O-C-C-C*H_2*-$(CH_2)_2$-N, $^{3}J_{H,H}$ = 6.2 Hz), 1.90-2.01 (4H, m, (*Coum*)O-C-C-CH-C-CH_2-C*H_2*-CH_2-N, (*Coum*)O-C-C-CH_2-C*H_2*-CH_2-N), 1.93 (2H, sept, 2x(*Ar*)C-CH-CH-C-C*H*$(CH_3)_2$, $^{3}J_{H,H}$ = 6.9 Hz), 1.68 (6H, s, 2xC*H_3*-(*Ar*)C-CH-CH-C), 1.59-1.75 (4H, m, C(*Coum*)-(C=O)-NH-CH_2-C*H_2*-$(CH_2)_2$-NH_2, C(*Coum*)-(C=O)-NH-$(CH_2)_2$-C*H_2*-CH_2-NH_2), 1.37 (9H, s, S-CH_2-(*Ar*)C-CH-CH-C-C(C*H_3*)$_3$), 1.33 (9H, s, S-CH_2-(*Ar*)C-CH-CH-C-C(C*H_3*)$_3$), 0.94 (6H, d, (*Ar*)C-CH-CH-C-CH(C*H_3*)$_2$, $^{3}J_{H,H}$ = 6.9 Hz), 0.90 (6H, d, (*Ar*)C-CH-CH-C-CH(C*H_3*)$_2$, $^{3}J_{H,H}$ = 6.9 Hz); **^{13}C-NMR ($CDCl_3$) δ*C*, ppm:** 171.5 (1C, S-(*Ar*)C-CH-CH-C-CH_2-(*C*=O)-NH), 163.6 (1C, (*Coum*)O-C-C-CH-C-(*C*=O)-NH), 163.0 (1C, (*Coum*)O-C-C-CH-C-(*C*=O)), 152.7 (1C, (*Coum*)O-*C*-C-CH-C-(C=O)), 151.96, 151.92 (2C, 2xS-CH_2-(*Ar*)C-CH-CH-*C*-C$(CH_3)_3$), 148.1 (2C, (*Coum*)O-C-C-*C*H-C-(C=O)), (*Ar*)*C*-N-CH_2-CH_2), 138.7 (1C, S-(*Ar*)C-CH-CH-*C*-CH_2-(C=O)-NH), 136.8, 136.6 (2C, 2xS-CH_2-(*Ar*)*C*-CH-CH-C-C$(CH_3)_3$), 134.7 (1C, S-(*Ar*)*C*-CH-CH-C-CH_2-(C=O)-NH), 132.4 (2C, 2xS-(*Ar*)C-*C*H-CH-C-CH_2-(C=O)-NH), 130.6 (2C, 2xS-(*Ar*)C-CH-*C*H-C-CH_2-(C=O)-NH), 129.4, 129.1 (4C, 4xS-CH_2-(*Ar*)C-*C*H-CH-C-C$(CH_3)_3$), 127.1 (1C, (*Coum*)O-C-C-*C*H-C-$(CH_2)_3$-N), 125.7, 125.6 (4C, 4xS-CH_2-(*Ar*)C-CH-*C*H-C-C$(CH_3)_3$), 119.6 (1C, (*Coum*)O-C-*C*-$(CH_2)_3$-N), 109.4 (1C, (*Coum*)O-C-C-CH-*C*-(C=O)-NH), 108.4 (1C, (*Coum*)O-C-*C*-CH-C-(C=O)-NH), 107.4 (2C, 2xCH_3-(*Ar*)C-CH-CH-*C*), 105.7 (1C, (*Coum*)O-C-C-CH-*C*-$(CH_2)_3$-N), 100.5 (2C, 2xCH_3-(*Ar*)*C*-CH-CH-C), 84.0 (2C, 2xCH_3-(*Ar*)C-CH-*C*H-C), 83.76 (2C, 2xCH_3-(*Ar*)C-CH-*C*H-C), 83.73 (2C, 2xCH_3-(*Ar*)C-*C*H-CH-C, 82.4 (2C, 2xCH_3-(*Ar*)C-*C*H-CH-C), 50.4 (1C, (*Coum*)O-C-C-CH-C-$(CH_2)_2$-*C*H_2-N), 49.9 (1C, (*Coum*)O-C-C-$(CH_2)_2$-*C*H_2-N), 43.2 (1C, S-(*Ar*)C-CH-CH-C-*C*H_2-(C=O)-NH), 40.0 (1C, S-*C*H_2-(*Ar*)C-CH-CH-C-C$(CH_3)_3$), 39.5 (1C, NH-$(CH_2)_3$-*C*H_2-NH-(C=O)-C(*Coum*)), 39.4 (1C, S-*C*H_2-(*Ar*)C-CH-CH-C-C$(CH_3)_3$), 39.3 (1C, NH-*C*H_2-$(CH_2)_3$-NH-(C=O)-C(*Coum*)), 34.94 (1C, S-CH_2-(*Ar*)C-CH-CH-C-*C*$(CH_3)_3$), 34.90 (1C, S-CH_2-(*Ar*)C-CH-CH-C-*C*$(CH_3)_3$), 31.6 (6C, 2xS-CH_2-(*Ar*)C-CH-CH-C-C(*C*H_3)$_3$), 31.0 (2C, 2x(*Ar*)CH-CH-C-*C*H$(CH_3)_2$), 27.6 (1C, (*Coum*)O-C-C-CH-C-*C*H_2-$(CH_2)_2$-N), 27.4 (1C, NH-$(CH_2)_2$-*C*H_2-CH_2-NH-(C=O)-C(*Coum*)), 27.0 (1C, NH-CH_2-*C*H_2-$(CH_2)_2$-NH-(C=O)-C(*Coum*)), 23.2 (2C, (*Ar*)CH-CH-C-CH(*C*H_3)$_2$), 22.9 (2C, (*Ar*)CH-CH-C-CH(*C*H_3)$_2$), 21.3 (1C, (*Coum*)O-C-C-CH-C-CH_2-*C*H_2-CH_2-N), 20.4 (1C, (*Coum*)O-C-C-CH_2-*C*H_2-CH_2-N), 20.3 (1C, (*Coum*)O-C-C-*C*H_2-$(CH_2)_2$-N), 18.2 (2C, 2x*C*H_3-(*Ar*)C-CH-CH); **HRMS (ESI(+)):** *m/z* calcd for $C_{70}H_{88}N_3O_4Ru_2S_3^{+}$: 1334.4018 $[M-Cl]^{+}$, and for $C_{70}H_{89}N_3O_4Ru_2S_3^{+2}$: 667.7046 $[M-Cl+H]^{+2}$; found: 1334.3999, 667.2009 (the isotopic pattern corresponds well to the

calculated one); **elemental analysis** calcd (%) for $C_{70}H_{88}ClN_3O_4Ru_2S_3\cdot0.5CH_3OH$: C 61.13, H 6.55, N 3.03; found C 61.14, H 7.24, N 2.73.

Synthesis of [(η^6-*p*-MeC$_6$H$_4$^{i}Pr)$_2$Ru$_2$(SCH$_2$C$_6$H$_4$^{t}Bu)$_2$(SC$_6$H$_4$-*p*-CH$_2$CO$_2$NH(CH$_2$)$_4$NHR)]Cl (R = 11-oxo-2,3,6,7-tetrahydro-1*H*,5*H*,11*H*-pyrano[2,3-*f*]pyrido[3,2,1-*ij*]quinoline-10-carboxylate) (17b)

To a solution of **4b** (0.160 g, 0.152 mmol, 1 equiv), in dry CH_2Cl_2 (50 mL) were added were added HOBt·H_2O (0.062 g, 0.365 mmol, 2.4 equiv) and DIPEA (0.066 mL, 0.365 mmol, 2.4 equiv). After 10 min were added successively EDCI (0.087 g, 0.456 mmol, 3 equiv), **10** (0.070 g, 0.197 mmol, 1.3 equiv), and DIPEA (0.066 mL, 0.365 mmol, 2.5 equiv). The reaction mixture was stirred at room temperature for 24 h, the reaction evolution was verified by ^{1}H NMR ($CDCl_3$) and TLC and then the mixture was concentrated to dryness under reduced pressure. Purification by flash column chromatography using a CH_2Cl_2/MeOH mixture as eluent afforded **17b** as an orange solid (0.025 g, 0.018 mmol, yield 12%).

***R*f** (CH_2Cl_2/CH_3OH 9:1) = 0.400; **^{1}H-NMR ($CDCl_3$) δ_H, ppm:** 8.84 (1H, t br, S-(*Ar*)C-CH-CH-C-CH$_2$-(C=O)-N$\underline{H}$, $^3J_{H,H}$ = 5.6 Hz), 8.55 (1H, s, (*Coum*)O-C-C-C$\underline{H}$-C-(C=O)), 8.17-8.24 (1H, t br, O-C-C-CH-C-(C=O)-N$\underline{H}$), 7.67-7.77 (8H, m, 4xCH$_2$-(*Ar*)C-C$\underline{H}$-CH-C-CF$_3$, 4xCH$_2$-(*Ar*)C-CH-C$\underline{H}$-C-CF$_3$), 7.65 (2H, d, 2xS-(*Ar*)C-CH-C$\underline{H}$-C-CH$_2$-(C=O)-NH, $^3J_{H,H}$ = 8.0 Hz), 7.51 (2H, d, 2xS-(*Ar*)C-C$\underline{H}$-CH-C-CH$_2$-(C=O)-NH, $^3J_{H,H}$ = 8.0 Hz), 6.96 (1H, s, (*Coum*)O-C-C-C$\underline{H}$-C-CH$_2$), 5.14 (2H, d, 2xCH$_3$-(*Ar*)C-CH-C$\underline{H}$-C, $^3J_{H,H}$ = 5.7 Hz), 5.06 (2H, d, 2xCH$_3$-(*Ar*)C-C$\underline{H}$-CH-C, $^3J_{H,H}$ = 5.8 Hz), 4.94 (2H, d, 2xCH$_3$-(*Ar*)C-CH-C$\underline{H}$-C, $^3J_{H,H}$ = 5.7 Hz), 4.79 (2H, d, 2xCH$_3$-(*Ar*)C-C$\underline{H}$-CH-C, $^3J_{H,H}$ = 5.7 Hz), 3.73 (2H, s, S-C$\underline{H_2}$-(*Ar*)C-CH-CH-C-CF$_3$), 3.70 (2H, s, S-(*Ar*)C-CH-CH-C-C$\underline{H_2}$-(C=O)-NH), 3.56 (2H, s, S-C$\underline{H_2}$-(*Ar*)C-CH-CH-C-CF$_3$), 3.42 (4H, qvart br, NH-(CH$_2$)$_3$-C$\underline{H_2}$-NH-(C=O)-C(*Coum*), $^3J_{H,H}$ = 5.7 Hz), 3.25-3.33 (6H, m, NH-C$\underline{H_2}$-(CH$_2$)$_3$-NH-(C=O)-C(*Coum*), (*Coum*)O-C-C-CH-C-(CH$_2$)$_2$-C$\underline{H_2}$-N, (*Coum*)O-C-C-(CH$_2$)$_2$-C$\underline{H_2}$-N), $^3J_{H,H}$ = 5.7 Hz), 2.82 (2H, t, (*Coum*)O-C-C-CH-C-C$\underline{H_2}$-(CH$_2$)$_2$-N, $^3J_{H,H}$ = 6.4 Hz), 2.74 (2H, t, (*Coum*)O-C-C-C$\underline{H_2}$-(CH$_2$)$_2$-N, $^3J_{H,H}$ = 6.2 Hz), 1.91-2.02 (4H, m, (*Coum*)O-C-C-CH-C-CH$_2$-C$\underline{H_2}$-CH$_2$-N, (*Coum*)O-C-C-CH$_2$-C$\underline{H_2}$-CH$_2$-N), 1.89 (2H, sept, 2x(*Ar*)C-CH-CH-C-C$\underline{H}$(CH$_3$)$_2$, $^3J_{H,H}$ = 6.8 Hz), 1.70 (6H, s, 2xC$\underline{H_3}$-(*Ar*)C-CH-CH-C), 1.62-1.66 (4H, m, NH-CH$_2$-C$\underline{H_2}$-(CH$_2$)$_2$-NH-(C=O)-C(*Coum*), NH-(CH$_2$)$_2$-C$\underline{H_2}$-CH$_2$-NH-(C=O)- C(*Coum*)), 0.98 (6H, d, (*Ar*)C-CH-CH-C-CH(C$\underline{H_3}$)$_2$, $^3J_{H,H}$ = 6.8 Hz), 0.90 (6H, d, (*Ar*)C-CH-CH-C-CH(C$\underline{H_3}$)$_2$, $^3J_{H,H}$ = 6.8 Hz); **^{13}C-NMR ($CDCl_3$) δ_C, ppm:** 171.2 (1C, S-(*Ar*)C-CH-CH-C-CH$_2$-($\underline{C}$=O)-NH), 163.7 (1C, (*Coum*)O-C-C-CH-C-($\underline{C}$=O)-NH), 163.1 (1C, (*Coum*)O-C-C-CH-C-($\underline{C}$=O)), 152.7 (1C, (*Coum*)O-$\underline{C}$-C-CH-C-(C=O)), 148.2 (1C, (*Ar*)$\underline{C}$-N-CH$_2$-CH$_2$), 148.1 (1C, (*Coum*)O-C-C-$\underline{C}$H-C-(C=O)), 144.0, 143.9 (2C, 2xS-CH$_2$-(*Ar*)$\underline{C}$-CH-CH-C-CF$_3$), 138.4 (1C, S-(*Ar*)C-CH-CH-$\underline{C}$-CH$_2$-(C=O)-NH), 134.6 (1C, S-(*Ar*)$\underline{C}$-CH-CH-C-CH$_2$-(C=O)-NH), 132.6 (2C, 2xS-(*Ar*)C-CH-$\underline{C}$H-C-CH$_2$-(C=O)-NH), 130.6 (2C, 2xS-(*Ar*)C-$\underline{C}$H-CH-C-CH$_2$-(C=O)-NH), 130.7 (1C, m, S-CH$_2$-(*Ar*)C-CH-CH-$\underline{C}$-CF$_3$), 130.4 (1C, m, S-CH$_2$-(*Ar*)C-CH-CH-$\underline{C}$-CF$_3$), 130.1, 129.8 (4C, 4xS-CH$_2$-(*Ar*)C-$\underline{C}$H-CH-C-CF$_3$), 127.1 (1C, (*Coum*)O-C-C-$\underline{C}$H-C-(CH$_2$)$_3$-N), 125.9 (2C, m, 2xS-CH$_2$-(*Ar*)C-CH-$\underline{C}$H-C-CF$_3$, $^3J_{C,F}$ = 4 Hz), 125.7 (2C, m, 2xS-CH$_2$-(*Ar*)C-CH-$\underline{C}$H-C-CF$_3$, $^3J_{C,F}$ = 4 Hz), 124.0 (2C, qvart, 2xS-CH$_2$-(*Ar*)C-CH-CH-C-$\underline{C}$F$_3$, $^1J_{C,F}$ = 272 Hz), 119.7 (1C, (*Coum*)O-C-$\underline{C}$-(CH$_2$)$_3$-N), 109.2 (1C, (*Coum*)O-C-C-CH-$\underline{C}$-(C=O)-NH), 108.3 (1C, (*Coum*)O-C-$\underline{C}$-CH-C-(C=O)-NH), 107.9 (2C, 2xCH$_3$-(*Ar*)C-CH-CH-$\underline{C}$), 105.7 (1C, (*Coum*)O-C-C-CH-$\underline{C}$-(CH$_2$)$_3$-N), 100.6 (2C, 2xCH$_3$-(*Ar*)$\underline{C}$-CH-CH-C), 84.3 (2C, 2xCH$_3$-(*Ar*)C-CH-$\underline{C}$H-C), 84.1 (2C, 2xCH$_3$-(*Ar*)C-$\underline{C}$H-CH-C), 83.7 (2C, 2xCH$_3$-(*Ar*)C-CH-$\underline{C}$H-C), 82.3 (2C, 2xCH$_3$-(*Ar*)C-$\underline{C}$H-CH-C), 50.3 (1C, (*Coum*)O-C-C-CH-C-(CH$_2$)$_2$-$\underline{C}$H$_2$-N), 49.9 (1C, (*Coum*)O-C-C-(CH$_2$)$_2$-$\underline{C}$H$_2$-N), 43.2 (1C, S-

(*Ar*)C-CH-CH-C-$\underline{C}H_2$-(C=O)-NH), 40.0 (1C, S-$\underline{C}H_2$-(*Ar*)C-CH-CH-C-CF_3), 39.7 (1C, S-$\underline{C}H_2$-(*Ar*)C-CH-CH-C-CF_3), 39.4 (2C, NH-$(CH_2)_3$-$\underline{C}H_2$-NH-(C=O)-C(*Coum*), NH-$\underline{C}H_2$-$(CH_2)_3$-NH-(C=O)-C(*Coum*)), 31.1 (2C, 2x(*Ar*)CH-CH-C-$\underline{C}H(CH_3)_2$), 27.6 (1C, (1C, (*Coum*)O-C-C-CH-C-$\underline{C}H_2$-$(CH_2)_2$-N), 27.4 (1C, NH-$(CH_2)_2$-$\underline{C}H_2$-CH_2-NH-(C=O)-C(*Coum*)), 26.9 (1C, NH-CH_2-$\underline{C}H_2$-$(CH_2)_2$-NH-(C=O)-C(*Coum*)), 23.2 (2C, (*Ar*)CH-CH-C-CH($\underline{C}H_3)_2$), 22.7 (2C, (*Ar*)CH-CH-C-CH($\underline{C}H_3)_2$), 21.3 (1C, (*Coum*)O-C-C-CH-C-CH_2-$\underline{C}H_2$-CH_2-N), 20.3 (1C, (*Coum*)O-C-C-CH_2-$\underline{C}H_2$-CH_2-N), 20.2 (1C, (*Coum*)O-C-C-$\underline{C}H_2$-$(CH_2)_2$-N), 18.1 (2C, 2x$\underline{C}H_3$-(*Ar*)C-CH-CH); **^{19}F-NMR ($CDCl_3$) δ_F, ppm:** -62.34, -62.39 (6F, 2xC$\underline{F_3}$); **HRMS (ESI(+)):** *m/z* calcd for $C_{64}H_{70}F_6N_3O_4Ru_2S_3^+$: 1358.2514 [M-Cl]$^+$, and for $C_{64}H_{70}F_6N_3NaO_4Ru_2S_3^{+2}$: 690.6203 [M-Cl+Na]$^{+2}$; found 1358.2509, 690.6201 (the isotopic pattern corresponds well to the calculated one); **elemental analysis** calcd (%) for $C_{64}H_{70}ClF_6N_3O_4Ru_2S_3$·2.5$C_6H_{14}$·$CH_3OH$: C 58.57, H 6.70, N 2.56; found C 58.60, H 6.77, N 6.94.

Synthesis of [(η^6-*p*-$MeC_6H_4Pr^i)_2Ru_2(\mu_2$-$SC_6H_4$-*p*-OH$)_2]Cl_2$ (18)

To a solution of dimer ([Ru(η^6-*p*-$MeC_6H_4Pr^i$)Cl]$_2Cl_2$) (2.000 g, 3.266 mmol, 1 equiv) in EtOH (150 mL) at 0°C under inert atmosphere (N_2) was added dropwise a solution of 4-hydroxy-benzenethiol (0.824 g, 6.532 mmol, 2 equiv) in EtOH (20 mL). The reaction mixture was stirred at 0°C for further 4 h and then concentrated at 40°C under reduced pressure to dryness. Purification by column chromatography using CH_2Cl_2/CH_3OH mixture afforded **18** as an orange solid (1.292 g, 1.632 mmol, yield 50%).

***R*f** (CH_2Cl_2/CH_3OH 10:1) = 0.413; **^{1}H-NMR ($CDCl_3$) δ_H, ppm:** 7.74-7.91 (3H, m, 3x(*Ar*)C-C$\underline{H}$-CH-C-OH), 7.38-7.52 (1H, m, (*Ar*)C-C$\underline{H}$-CH-C-OH), 6.91-7.03 (4H, m, 4x(*Ar*)C-CH-C$\underline{H}$-C-OH), 5.11-5.24 (4H, m, 2xCH_3-(*Ar*)C-CH-C$\underline{H}$-C, 2xCH_3-(*Ar*)C-CH-C$\underline{H}$-C), 5.00-5.11 (2H, m, 2xCH_3-(*Ar*)C-C$\underline{H}$-CH-C), 4.86-5.00 (2H, m, 2xCH_3-(*Ar*)C-C$\underline{H}$-CH-C), 2.37 (2H, sept, 2x(*Ar*)C-CH-CH-C-C$\underline{H}(CH_3)_2$, $^3J_{H,H}$ = 6.7 Hz), 2.67 (6H, m, 2xC$\underline{H_3}$-(*Ar*)C-CH-CH-C), 1.00-1.15 (12H, m, 2x(*Ar*)C-CH-CH-C-CH(C$\underline{H_3})_2$); **^{13}C-NMR ($CDCl_3$) δ_C, ppm:** 158.9 (2C, 2xS-(*Ar*)C-CH-CH-$\underline{C}$-OH), 133.7 (2C, 2xS-(*Ar*)$\underline{C}$-CH-CH-C-OH), 133.1 (4C, 4xS-(*Ar*)C-$\underline{C}$H-CH-C-OH), 116.8 (4C, 4xS-(*Ar*)C-CH-$\underline{C}$H-C-OH), 106.1 (2C, 2xCH_3-(*Ar*)C-CH-CH-$\underline{C}$), 97.1 (2C, 2xCH_3-(*Ar*)$\underline{C}$-CH-CH-C), 84.2 (2C, 2xCH_3-(*Ar*)C-CH-$\underline{C}$H-C), 83.1 (2C, 2xCH_3-(*Ar*)C-$\underline{C}$H-CH-C), 82.2 (2C, 2xCH_3-(*Ar*)C-CH-$\underline{C}$H-C), 82.0 (2C, 2xCH_3-(*Ar*)C-$\underline{C}$H-CH-C), 30.8 (2C, 2x(*Ar*)CH-CH-C-$\underline{C}H(CH_3)_2$), 22.3 (4C, 2x(*Ar*)CH-CH-C-CH($\underline{C}H_3)_2$), 17.8 (2C, 2x$\underline{C}H_3$-(*Ar*)C-CH-CH); **HRMS (ESI(+)):** *m/z* calcd for $C_{32}H_{38}ClO_2Ru_2S_2^+$: 757.0083 [M-Cl]$^+$; found: 757.0077 (the isotopic pattern corresponds well to the calculated one).

Synthesis of [(η^6-*p*-$MeC_6H_4Pr^i)_2Ru_2(\mu_2$-$SCH_2C_6H_4$-*p*-$Bu^t)(\mu_2$-$SC_6H_4$-*p*-OH$)_2$]Cl (19)

The mixed trithiolto ruthenium(II)-arene complex **19** was prepared and purified by adapting a to formerly published protocol.[7]

To a solution of **18** (2.00 g, 2.22 mmol, 1 equiv) in refluxing EtOH (140 mL) was added dropwise under inert atmosphere (N_2) a solution of 4-*tert*-butylbenzenemethanethiol (0.809 g, 6.66 mmol, 3 equiv) in EtOH (10 mL). The reaction mixture was further heated at reflux under inert atmosphere (N_2) for 24 h. The reaction evolution was verified by TLC and then the reaction mixture was concentrated to dryness under reduced pressure. Purification by column chromatography using CH_2Cl_2/CH_3OH mixture as eluent afforded **8** as an orange solid (2.10 g, 2.132 mmol, yield 96%).

***R*f** (CH_2Cl_2/CH_3OH 10:1) = 0.364; **^{1}H-NMR ($CDCl_3$) δ_H, ppm:** 9.68 (2H, s br, 2xO$\underline{H}$), 7.65 (2H, d, 2xS-(*Ar*)C-C$\underline{H}$-CH-C-OH, $^3J_{H,H}$ = 8.6 Hz), 7.50 (2H, d, 2xS-(*Ar*)C-C$\underline{H}$-CH-C-OH, $^3J_{H,H}$ = 8.6 Hz), 7.41-7.46 (4H, m, 2xCH_2-(*Ar*)C-C$\underline{H}$-CH-C-C$(CH_3)_3$, 2xCH_2-(*Ar*)C-CH-C$\underline{H}$-C-C$(CH_3)_3$, $^3J_{H,H}$ = 8.7 Hz), 7.20 (2H, d, 2xS-(*Ar*)C-CH-C$\underline{H}$-C-OH, $^3J_{H,H}$ = 8.6 Hz), 7.15 (2H, d, 2xS-(*Ar*)C-CH-

C*H*-C-OH, $^{3}J_{H,H}$ = 8.6 Hz), 4.98 (2H, d, 2xCH$_3$-(*Ar*)C-CH-C*H*-C, $^{3}J_{H,H}$ = 5.8 Hz), 4.89 (2H, d, 2xCH$_3$-(*Ar*)C-CH-C*H*-C, $^{3}J_{H,H}$ = 5.5 Hz), 4.88 (2H, d, 2xCH$_3$-(*Ar*)C-C*H*-CH-C, $^{3}J_{H,H}$ = 5.5 Hz), 4.81 (2H, d, 2xCH$_3$-(*Ar*)C-C*H*-CH-C, $^{3}J_{H,H}$ = 5.8 Hz), 3.51 (2H, s, S-C*H$_2$*-(*Ar*)C-CH-CH-C-C(CH$_3$)$_3$), 1.95 (2H, sept, 2x(*Ar*)C-CH-CH-C-C*H*(CH$_3$)$_2$, $^{3}J_{H,H}$ = 6.8 Hz), 1.64 (6H, s, 2xC*H$_3$*-(*Ar*)C-CH-CH-C), 1.34 (9H, s, S-CH$_2$-(*Ar*)C-CH-CH-C-C(C*H$_3$*)$_3$), 0.92 (12H, d, 2x(*Ar*)C-CH-CH-C-CH(C*H$_3$*)$_2$, $^{3}J_{H,H}$ = 6.8 Hz); **^{13}C-NMR (CDCl$_3$) δ*$_C$*, ppm:** 159.4 (1C, S-(*Ar*)C-CH-CH-*C*-OH), 159.0 (1C, S-(*Ar*)C-CH-CH-*C*-OH), 151.7 (1C, S-CH$_2$-(*Ar*)C-CH-CH-*C*-C(CH$_3$)$_3$), 137.0 (1C, S-CH$_2$-(*Ar*)*C*-CH-CH-C-C(CH$_3$)$_3$), 133.6 (2C, 2xS-(*Ar*)C-*C*H-CH-C-OH), 133.5 (2C, 2xS-(*Ar*)C-*C*H-CH-C-OH), 129.4 (2C, 2xS-CH$_2$-(*Ar*)C-*C*H-CH-C-C(CH$_3$)$_3$), 126.5 (2C, 2xS-(*Ar*)*C*-CH-CH-C-OH), 125.6 (2C, 2xS-CH$_2$-(*Ar*)C-CH-*C*H-C-C(CH$_3$)$_3$), 117.1 (2C, 2xS-(*Ar*)C-CH-*C*H-C-OH), 116.9 (2C, 2xS-(*Ar*)C-CH-*C*H-C-OH), 107.4 (2C, 2xCH$_3$-(*Ar*)C-CH-CH-*C*), 99.6 (2C, 2xCH$_3$-(*Ar*)*C*-CH-CH-C), 84.8 (2C, 2xCH$_3$-(*Ar*)C-CH-*C*H-C), 84.1 (2C, 2xCH$_3$-(*Ar*)C-*C*H-CH-C), 84.0 (2C, 2xCH$_3$-(*Ar*)C-CH-*C*H-C), 82.6 (2C, 2xCH$_3$-(*Ar*)C-*C*H-CH-C), 38.4 (1C, S-*C*H$_2$-(*Ar*)C-CH-CH-C-C(CH$_3$)$_3$), 34.9 (1C, S-CH$_2$-(*Ar*)C-CH-CH-C-*C*(CH$_3$)$_3$), 31.6 (3C, S-CH$_2$-(*Ar*)C-CH-CH-C-C(*C*H$_3$)$_3$), 30.7 (2C, 2x(*Ar*)CH-CH-C-*C*H(CH$_3$)$_2$), 22.9 (2C, (*Ar*)CH-CH-C-CH(*C*H$_3$)$_2$), 22.7 (2C, (*Ar*)CH-CH-C-CH(*C*H$_3$)$_2$), 18.0 (2C, 2x*C*H$_3$-(*Ar*)C-CH-CH); **HRMS (ESI(+)):** *m/z* calcd for $C_{43}H_{53}O_2Ru_2S_3^+$: 901.1289 [M-Cl]$^+$; found: 901.1282 (the isotopic pattern corresponds well to the calculated one); **elemental analysis** calcd (%) for $C_{43}H_{53}ClO_2Ru_2S_3$·2CH$_3$OH: C 54.06, H 6.15; found C 54.17, H 6.54.

Synthesis of [(η^6-*p*-MeC$_6$H$_4$Pr^i)$_2$Ru$_2$(μ_2-SCH$_2$C$_6$H$_4$-*p*-Bu^t)(μ_2-SC$_6$H$_4$-*p*-O-R)$_2$]Cl (R = 7-(diethylamino)-2-oxo-2*H*-chromene-3-carboxylate) (20)

To a solution of **Dye2-CO$_2$H** (0.139 g, 0.505 mmol, 2.5 equiv) in dry CH$_2$Cl$_2$ (50 mL) was added EDCI (0.097 g, 0.505 mmol, 2.5 equiv) After 10 min were added successively **19** (0.200 g, 0.202 mmol, 1 equiv) and DMAP (0.015 g, 0.121 mmol, 0.6 equiv). The reaction mixture was stirred at room temperature for 24 h, the reaction evolution was verified by ^{1}H NMR (CDCl$_3$) and TLC and then the mixture was concentrated to dryness under reduced pressure. Purification by flash column chromatography using a CH$_2$Cl$_2$/MeOH mixture as eluent afforded **20** as an orange solid (0.215 g, 0.151 mmol, yield 75%).

***R*$_f$** (CH$_2$Cl$_2$/CH$_3$OH 10:1) = 0.429; **^{1}H-NMR (CDCl$_3$) δ*$_H$*, ppm:** 8.70 (1H, s, (*Coum*)O-C-C-C*H*-C-(C=O)), 8.66 (1H, s, (*Coum*)O-C-C-C*H*-C-(C=O)), 7.95 (2H, d, 2xS-(*Ar*)C-CH-C*H*-C-O, $^{3}J_{H,H}$ = 8.3 Hz), 7.80 (2H, d, 2xS-(*Ar*)C-CH-C*H*-C-O, $^{3}J_{H,H}$ = 8.3 Hz), 7.50 (1H, d, (*Coum*)O-C-C-C*H*-CH-C-N(CH$_2$-CH$_3$)$_2$, $^{3}J_{H,H}$ = 6.8 Hz), 7.48 (1H, d, (*Coum*)O-C-C-C*H*-CH-C-N(CH$_2$-CH$_3$)$_2$, $^{3}J_{H,H}$ = 6.8 Hz), 7.42-7.47 (4H, m, 2xS-CH$_2$-(*Ar*)C-CH-C*H*-C-C(CH$_3$)$_3$, 2xS-CH$_2$-(*Ar*)C-C*H*-CH-C-C(CH$_3$)$_3$), 7.27 (2H, d, 2xS-(*Ar*)C-C*H*-CH-C-O, $^{3}J_{H,H}$ = 8.3 Hz), 7.21 (2H, d, 2xS-(*Ar*)C-C*H*-CH-C-O, $^{3}J_{H,H}$ = 8.3 Hz), 6.67 (2H, dd, 2xO-C-C-CH-C*H*-C-N(CH$_2$-CH$_3$)$_2$, $^{3}J_{H,H}$ = 9.0 Hz), 6.49 (2H, m, 2x(*Coum*)O-C-C*H*-C-N(CH$_2$-CH$_3$)$_2$), 5.12 (4H, m, 2xCH$_3$-(*Ar*)C-CH-C*H*-C, 2xCH$_3$-(*Ar*)C-C*H*-CH-C), 5.07 (2H, d, 2xCH$_3$-(*Ar*)C-CH-C*H*-C, $^{3}J_{H,H}$ = 5.7 Hz), 4.93 (2H, d, 2xCH$_3$-(*Ar*)C-C*H*-CH-C, $^{3}J_{H,H}$ = 5.7 Hz), 3.62 (2H, s, S-C*H$_2$*-(*Ar*)C-CH-CH-C-C(CH$_3$)$_3$), 3.48 (8H, qvart, 2xN(C*H$_2$*-CH$_3$)$_2$, $^{3}J_{H,H}$ = 7.0 Hz), 1.92 (2H, sept, 2x(*Ar*)-C-CH-CH-C-C*H*(CH$_3$)$_2$, $^{3}J_{H,H}$ = 6.4 Hz), 1.72 (6H, s, 2xC*H$_3$*-(*Ar*)C-CH-CH-C), 1.34 (9H, s, S-CH$_2$-(*Ar*)C-CH-CH-C-C(C*H$_3$*)$_3$), 1.26 (12H, t, 2xN(CH$_2$-C*H$_3$*)$_2$, $^{3}J_{H,H}$ = 7.0 Hz), 0.92 (12H, d, 2x(*Ar*)C-CH-CH-C-CH(C*H$_3$*)$_2$, $^{3}J_{H,H}$ = 6.4 Hz); **^{13}C-NMR (CDCl$_3$) δ*$_C$*, ppm:** 162.28, 162.25 (2C, 2xS-(*Ar*)-C-CH-CH-C-O-(*C*=O)), 159.01, 158.99 (2C, 2x(*Coum*)O-*C*-C-CH-C-(C=O)), 158.4, 158.3 (2C, 2x(*Coum*)O-C-C-CH-C-(*C*=O)), 153.72, 153.69 (2C, 2x(*Ar*)-*C*-N(CH$_2$-CH$_3$)$_2$), 151.9 (1C, S-CH$_2$-(*Ar*)C-CH-CH-*C*-C(CH$_3$)$_3$), 151.6, 151.3 (2C, 2xS-(*Ar*)C-CH-CH-

C-O-(C=O)), 150.7, 150.6 (2C, 2x(*Coum*)O-C-C-CH-C-(C=O)), 136.7 (1C, S-CH$_2$-(*Ar*)C-CH-CH-C-C(CH$_3$)$_3$), 135.7, 134.9 (2C, 2xS-(*Ar*)C-CH-CH-C-O-(C=O)), 133.7, 133.5 (4C, 4xS-(*Ar*)C-CH-CH-C-O-(C=O)), 131.96, 131.91 (2C, 2x(*Coum*)O-C-C-CH-CH-C-N(CH$_2$-CH$_3$)$_2$), 129.5 (2C, 2xS-CH$_2$-(*Ar*)C-CH-CH-C-C(CH$_3$)$_3$), 125.7 (2C, 2xS-CH$_2$-(*Ar*)C-CH-CH-C-C(CH$_3$)$_3$), 123.0, 122.8 (4C, 4xS-(*Ar*)-C-CH-CH-C-O-(C=O)), 110.2 (2C, 2x(*Coum*)O-C-C-CH-CH-C-N(CH$_2$-CH$_3$)$_2$), 107.99, 107.95 (2C, 2x(*Coum*)O-C-C-CH-C-(C=O)), 107.8 (2C, 2xCH$_3$-(*Ar*)C-CH-CH-C), 106.9, 106.8 (2C, 2x(*Coum*)O-C-C-CH-C-(C=O)), 100.3 (2C, 2xCH$_3$-(*Ar*)C-CH-CH-C), 96.8 (2C, 2x(*Coum*)O-C-CH-C-N(CH$_2$-CH$_3$)$_2$), 84.9 (2C, 2xCH$_3$-(*Ar*)C-CH-CH-C), 85.57 (2C, 2xCH$_3$-(*Ar*)C-CH-CH-C), 85.53 (2C, 2xCH$_3$-(*Ar*)C-CH-CH-C), 82.9 (2C, 2xCH$_3$-(*Ar*)C-CH-CH-C), 45.4 (4C, 2xN(CH$_2$-CH$_3$)$_2$), 38.9 (1C, S-CH$_2$-(*Ar*)C-CH-CH-C-C(CH$_3$)$_3$), 34.9 (1C, S-CH$_2$-(*Ar*)C-CH-CH-C-C(CH$_3$)$_3$), 31.5 (3C, S-CH$_2$-(*Ar*)C-CH-CH-C-C(CH$_3$)$_3$), 29.8 (2C, 2x(*Ar*)C-CH-CH-C-CH(CH$_3$)$_2$), 22.8 (2C, (*Ar*)C-CH-CH-C-CH(CH$_3$)$_2$), 22.6 (2C, (*Ar*)C-CH-CH-C-CH(CH$_3$)$_2$), 18.1 (2C, 2xCH$_3$-(*Ar*)C-CH-CH), 12.6 (4C, 2xN(CH$_2$-CH$_3$)$_2$); **HRMS (ESI(+)):** *m/z* calcd for $C_{71}H_{79}N_2O_8Ru_2S_3^+$: 1387.3080 [M-Cl]$^+$, and for $C_{71}H_{79}N_2NaO_8Ru_2S_3^{+2}$: 705.1486 [M-Cl+Na]$^{+2}$; found 1387.3135, 705.1511 (the isotopic pattern corresponds well to the calculated one); **elemental analysis** calcd (%) for $C_{71}H_{79}ClN_2O_8Ru_2S_3 \cdot 0.25CH_2Cl_2 \cdot CH_3OH \cdot 0.25H_2O$: C 58.64, H 5.72, N 1.89; found C 58.61, H 5.75, N 4.19.

Synthesis of [(η^6-*p*-MeC$_6$H$_4$Pr^i)$_2$Ru$_2$(μ_2-SC$_6$H$_4$-*p*-OH)$_3$]Cl (21)

The tri-hydroxy intermediate **21** was obtained by adapting a previously reported procedure.[3]

To a solution of ([Ru(η^6-*p*-MeC$_6$H$_4$Pr^i)Cl]$_2$Cl$_2$) (1.00 g, 1.666 mmol, 1 equiv) in EtOH (150 mL) at reflux under inert atmosphere (N_2) was added dropwise a solution of 4-hydroxybenzenethiol (0.84 g, 6.666 mmol, 4 equiv) in EtOH (10 mL). After heating at reflux for 24 h, the reaction mixture was allowed to cool down to r.t. then concentrated under reduced pressure. Purification by column chromatography using CH_2Cl_2/CH_3OH mixture as eluent afforded **21** as an orange solid (1.47 g, 1.650 mmol, yield 98%).

***R*f** (CH_2Cl_2/CH_3OH 9:1) = 0.367; **^{1}H-NMR (MeOD-*d$_4$*) δ_H, ppm:** 7.76 (6H, m, 6xS-(*Ar*)C-CH-CH-C-OH, $^3J_{H,H}$ = 8.7 Hz), 6.80 (6H, m, 6xS-(*Ar*)C-CH-CH-C-OH, $^3J_{H,H}$ = 8.7 Hz), 5.40 (2H, d, 2xCH$_3$-(*Ar*)C-CH-CH-C, $^3J_{H,H}$ = 5.8 Hz), 5.33 (2H, d, 2xCH$_3$-(*Ar*)C-CH-CH-C, $^3J_{H,H}$ = 5.8 Hz), 5.21 (2H, d, 2xCH$_3$-(*Ar*)C-CH-CH-C, $^3J_{H,H}$ = 5.6 Hz), 5.17 (2H, d, 2xCH$_3$-(*Ar*)C-CH-CH-C, $^3J_{H,H}$ = 5.9 Hz), 1.99 (2H, sept, 2x(*Ar*)C-CH-CH-C-CH(CH$_3$)$_2$, $^3J_{H,H}$ = 6.9 Hz), 1.64 (6H, s, 2xCH$_3$-(*Ar*)C-CH-CH-C), 0.94 (6H, d, (*Ar*)C-CH-CH-C-CH(CH$_3$)$_2$, $^3J_{H,H}$ = 6.9 Hz), 0.85 (6H, d, (*Ar*)C-CH-CH-C-CH(CH$_3$)$_2$, $^3J_{H,H}$ = 6.9 Hz); **^{13}C-NMR (MeOD-*d$_4$*) δ_C, ppm:** 159.3 (3C, 3xS-(*Ar*)C-CH-CH-C-OH), 135.2 (6C, 6xS-(*Ar*)C-CH-CH-C-OH), 128.5 (3C, 3xS-(*Ar*)C-CH-CH-C-OH), 116.9 (6C, 6xS-(*Ar*)C-CH-CH-C-OH), 108.1 (2C, 2xCH$_3$-(*Ar*)C-CH-CH-C), 100.9 (2C, 2xCH$_3$-(*Ar*)C-CH-CH-C), 86.6 (2C, 2xCH$_3$-(*Ar*)C-CH-CH-C), 86.2 (2C, 2xCH$_3$-(*Ar*)C-CH-CH-C), 86.0 (2C, 2xCH$_3$-(*Ar*)C-CH-CH-C), 84.9 (2C, 2xCH$_3$-(*Ar*)C-CH-CH-C), 31.9 (2C, 2x(*Ar*)CH-CH-C-CH(CH$_3$)$_2$), 22.7 (2C, (*Ar*)CH-CH-C-CH(CH$_3$)$_2$), 22.5 (2C, (*Ar*)CH-CH-C-CH(CH$_3$)$_2$), 17.8 (2C, 2xCH$_3$-(*Ar*)C-CH-CH); **HRMS (ESI(+)):** *m/z* calcd for $C_{38}H_{43}O_3Ru_2S_3^+$: 847.0456 [M-Cl]$^+$; found: 847.0463 (the isotopic pattern corresponds well to the calculated one); **elemental analysis** calcd (%) for $C_{39}H_{47}ClO_4Ru_2S_3$ C 51.27, H 5.19; found C 51.27, H, 5.04.

Synthesis of ([(η^6-*p*-MeC$_6$H$_4$Pr^i)$_2$Ru$_2$(μ_2-SC$_6$H$_4$-*p*-O-R))$_3$]Cl) (R = 7-(diethylamino)-2-oxo-2*H*-chromene-3-carboxylate) (22)

To a solution of **Dye1-CO$_2$H** (0.296 g, 1.133 mmol, 4.0 equiv) in dry CH_2Cl_2 (60 mL) and dry DMF (5 mL) at r.t. under inert atmosphere was added EDCI (0.216 g, 1.130 mmol, 4.0 equiv), and the resulting mixture was stirred for 10 min. Then, **21** (0.250 g, 0.283 mmol, 1.0 equiv), HOBt·H_2O (0.172 g, 1.124 mmol, 4.0 equiv) and DIPEA (0.24 mL, 1.418 mmol, 5.0 equiv) were successively added and the resulting mixture was further stirred for 24 h. The reaction evolution was verified by TLC and the mixture was concentrated under reduced pressure. Purification by flash column chromatography using CH_2Cl_2/CH_3OH mixture as eluent afforded **22** as a yellow-orange solid (0.284 g, 0.173 mmol, yield 61%).

***R*$_f$** (CH_2Cl_2/CH_3OH 10:1) = 0.381; **^{1}H-NMR (CDCl$_3$) δ$_H$, ppm:** 8.68 (3H, s, 3x(*Coum*)O-C-C-C$\underline{H}$-C-(C=O)), 7.93 (6H, d, 6xS-(Ar)C-$\underline{C}$H-CH-C-O, $^3J_{H,H}$ = 8.4 Hz), 7.49 (3H, d, 3x(*Coum*)O-C-C-C$\underline{H}$-CH-C-N(CH$_2$-CH$_3$)$_2$, $^3J_{H,H}$ = 9.0 Hz), 7.28 (6H, d, 6xS-(Ar)C-CH-$\underline{C}$H-C-O, $^3J_{H,H}$ = 8.4 Hz), 6.69 (3H, dd, 3x(*Coum*)O-C-C-CH-C$\underline{H}$-C-N(CH$_2$-CH$_3$)$_2$, $^3J_{H,H}$ = 9.0 Hz, $^4J_{H,H}$ = 2.2 Hz), 6.51 (3H, d, 3x(*Coum*)O-C-C$\underline{H}$-C-N(CH$_2$-CH$_3$)$_2$, $^4J_{H,H}$ = 2.1 Hz), 5.41 (2H, d, 2xCH$_3$-(*Ar*)C-CH-C$\underline{H}$-C, $^3J_{H,H}$ = 5.4 Hz), 5.29 (2H, d, 2xCH$_3$-(*Ar*)C-CH-C$\underline{H}$-C, $^3J_{H,H}$ = 5.6 Hz), 5.17 (2H, d, 2xCH$_3$-(*Ar*)C-C$\underline{H}$-CH-C, $^3J_{H,H}$ = 5.5 Hz), 5.15 (2H, d, 2xCH$_3$-(*Ar*)C-C$\underline{H}$-CH-C, $^3J_{H,H}$ = 5.7 Hz), 3.48 (12H, qvart, 3xN(C$\underline{H_2}$-CH$_3$)$_2$, $^3J_{H,H}$ = 7.1 Hz), 2.02 (2H, sept, 2x(*Ar*)-C-CH-CH-C-C$\underline{H}$(CH$_3$)$_2$, $^3J_{H,H}$ = 6.8 Hz), 1.68 (6H, s, 2xC$\underline{H_3}$-(*Ar*)-C-CH-CH-C), 1.26 (18H, t, 3xN(CH$_2$-C$\underline{H_3}$)$_2$, $^3J_{H,H}$ = 7.1 Hz), 0.96 (6H, d, (*Ar*)C-CH-CH-C-CH(C$\underline{H_3}$)$_2$, $^3J_{H,H}$ = 6.8 Hz), 0.88 (6H, d, (*Ar*)C-CH-CH-C-CH(C$\underline{H_3}$)$_2$, $^3J_{H,H}$ = 6.8 Hz); **^{13}C-NMR (CDCl$_3$) δ$_C$, ppm:** 162.4 (3C, 3xS-(*Ar*)-C-CH-CH-C-O-($\underline{C}$=O)), 159.1 (3C, 3x(*Coum*)O-$\underline{C}$-C-CH-C-(C=O)), 158.4 (3C, 3x(*Coum*)O-C-C-CH-C-($\underline{C}$=O)), 153.7 (3C, 3x(*Ar*)-$\underline{C}$-N(CH$_2$-CH$_3$)$_2$), 151.6 (3C, 3xS-(*Ar*)C-CH-CH-$\underline{C}$-O-(C=O)), 150.7 (3C, 3x(*Coum*)O-C-C-$\underline{C}$H-C-(C=O)), 134.9 (3C, 3xS-(*Ar*)$\underline{C}$-CH-CH-C-O-(C=O)), 133.7 (6C, 6xS-(*Ar*)C-$\underline{C}$H-CH-C-O-(C=O)), 132.0 (3C, 3x(*Coum*)O-C-C-$\underline{C}$H-CH-C-N(CH$_2$-CH$_3$)$_2$), 123.1 (6C, 6xS-(*Ar*)-C-CH-$\underline{C}$H-C-O-(C=O)), 110.4 (3C, 3x(*Coum*)O-C-C-CH-$\underline{C}$H-C-N(CH$_2$-CH$_3$)$_2$), 108.3 (2C, 2xCH$_3$-(*Ar*)C-CH-CH-$\underline{C}$), 108.2 (3C, 3x(*Coum*)O-C-$\underline{C}$-CH-C-(C=O)), 107.1 (3C, 3x(*Coum*)O-C-C-CH-$\underline{C}$-(C=O)), 100.2 (2C, 2xCH$_3$-(*Ar*)$\underline{C}$-CH-CH-C), 97.1 (3C, 3x(*Coum*)O-C-$\underline{C}$H-C-N(CH$_2$-CH$_3$)$_2$), 86.0 (2C, 2xCH$_3$-(*Ar*)C-$\underline{C}$H-CH-C), 85.3 (2C, 2xCH$_3$-(*Ar*)C-CH-$\underline{C}$H-C), 85.0 (2C, 2xCH$_3$-(*Ar*)C-$\underline{C}$H-CH-C), 83.8 (2C, 2xCH$_3$-(*Ar*)C-CH-$\underline{C}$H-C), 45.6 (6C, 3xN($\underline{C}$H$_2$-CH$_3$)$_2$), 31.0 (2C, 2x(*Ar*)C-CH-CH-C-$\underline{C}$H(CH$_3$)$_2$), 22.8 (2C, (*Ar*)C-CH-CH-C-CH($\underline{C}$H$_3$)$_2$), 22.4 (2C, (*Ar*)C-CH-CH-C-CH($\underline{C}$H$_3$)$_2$), 18.0 (2C, 2x$\underline{C}$H$_3$-(*Ar*)C-CH-CH), 12.7 (6C, 3xN(CH$_2$-$\underline{C}$H$_3$)$_2$); **HRMS (ESI(+)):** *m/z* calcd for $C_{80}H_{82}N_3O_{12}Ru_2S_3^+$: 1576.3142 [M-Cl]$^+$, $C_{80}H_{82}N_3NaO_{12}Ru_2S_3^{+2}$: 799.6517 [M-Cl+Na]$^{+2}$, and for $C_{80}H_{83}N_3O_{12}Ru_2S_3^{+2}$: 788.6608 [M-Cl+H]$^{+2}$; found: 1576.3125, 799.6511, 788.6609 (the isotopic pattern corresponds well to the calculated one); **elemental analysis** calcd (%) for $C_{80}H_{82}ClN_3O_{12}Ru_2S_3$·$CH_3OH$·$H_2O$: C 58.56, H 5.34, N 2.53; found C 58.53, H 5.20, N 2.55.

Biology

(Immuno-)Fluorescence microscopy

Glass cover slips of 12 mm in diameter were placed in 24-well culture plate and sterilized by UV for 40 min. HFF in DMEM supplemented with 10% heat-inactivated, sterile, filtered FCS and 2% antibiotics (penicillin streptomycin) were seeded at 2×10^4 cells/mL and plates were allowed to grow for 3 days at 37°C with 5% CO_2. Culture medium was removed and replaced with fresh medium (1 mL/well) containing (i) 0.5%DMSO, (ii) 20 µM of **Dye2-CO_2H** or (iii) 20 µM of **12a**, and were then incubated for 1 h at 37°C / 5% CO_2. Subsequently the medium was discarded, coverslips were washed 3 times with sterile PBS, fixed with 2% paraformaldehyde (PFA) in PBS for 20 min, permeabilized (0.2 % Triton X-100 in PBS) for 5 min, and unspecific binding sites were blocked in blocking solution (3% BSA, 0.2% NaAcid in PBS) for 2 h at RT. Glass coverslips were then incubated for 30 min in monoclonal mouse anti-tubulin (clone B-5-1-2, Sigma) diluted 1:500 in PBS / 0.3% BSA. After washing with PBS, the secondary antibody conjugate (anti-mouse fluorescein isothiocyanate, FITC, Sigma) was applied at a dilution of 1:300 in PBS / 0.3% BSA. Cells were then stained with NucRed reagent (2 drops/mL) for 30 min at 37°C. Fluorescence microscopy was performed with a Nikon Eclipse E800 digital confocal fluorescence microscope. Images were acquired and processed with Openlab 5.5.2 software.

Structures of compounds

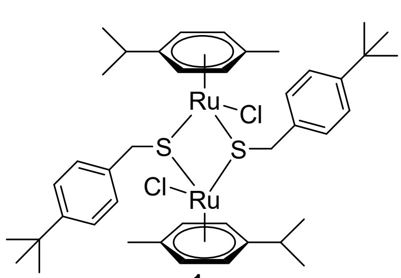

1a

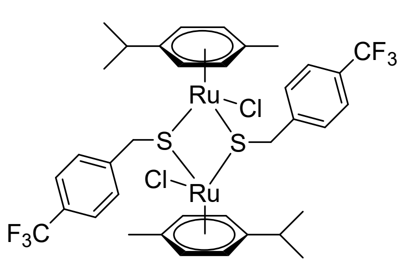

1b

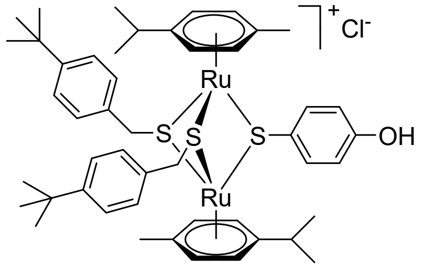

2a

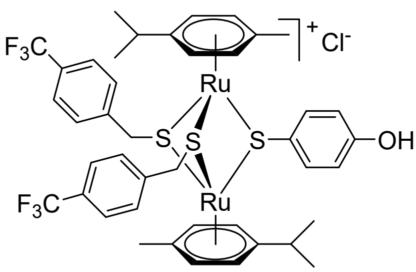

2b

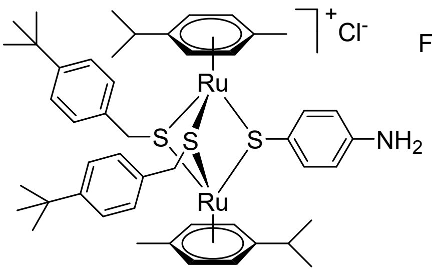

3a

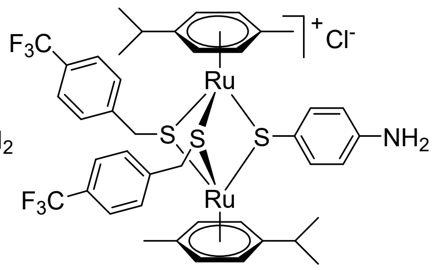

3b

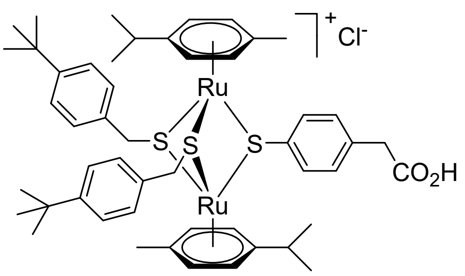

4a

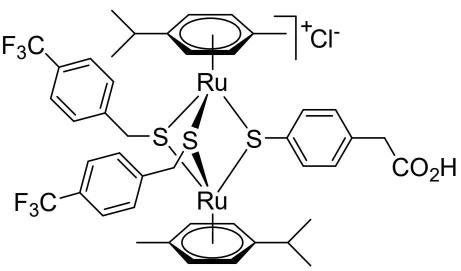

4b

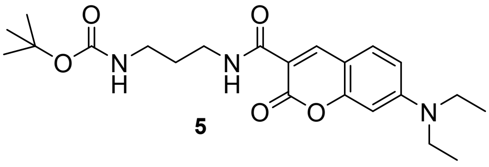

5

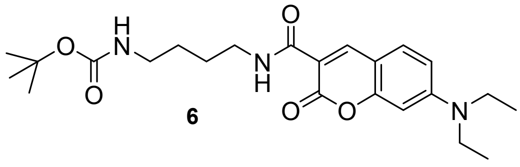

6

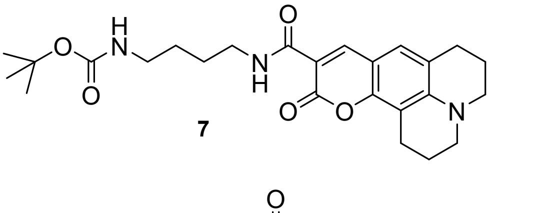

7

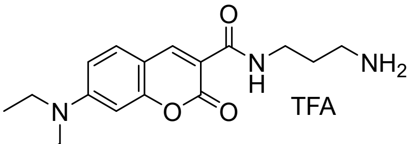

8

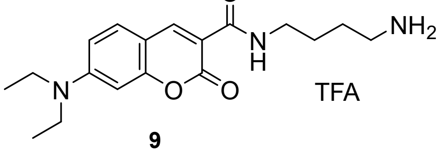

9

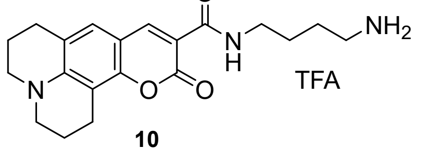

10

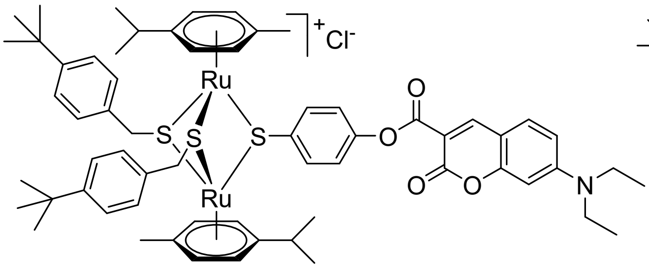

11

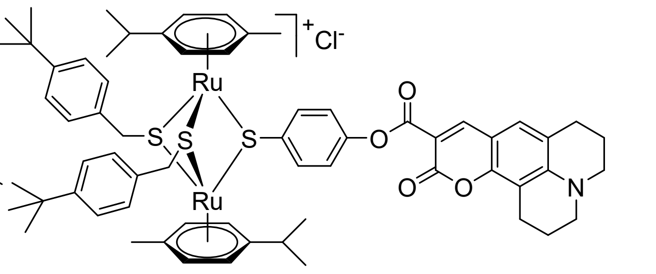

12a

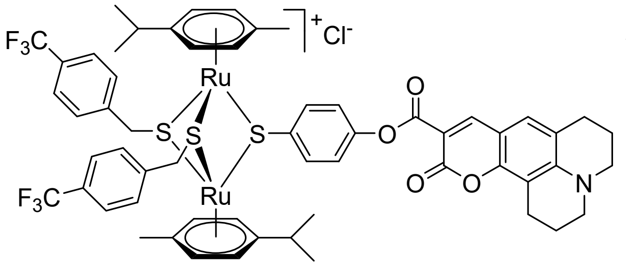

12b

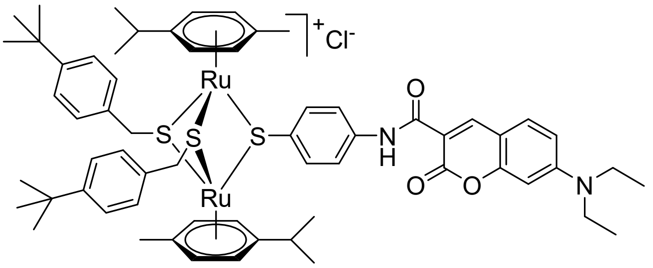

13

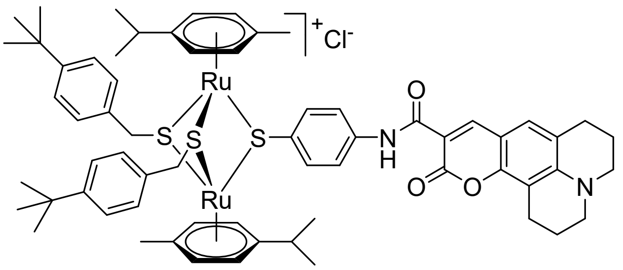

14a

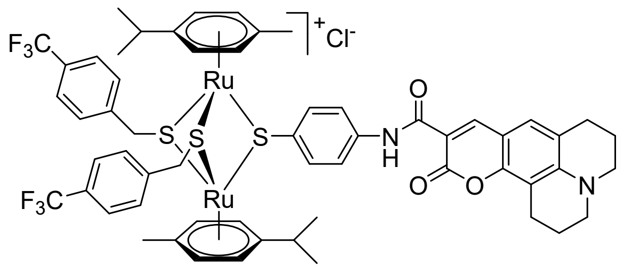

14b

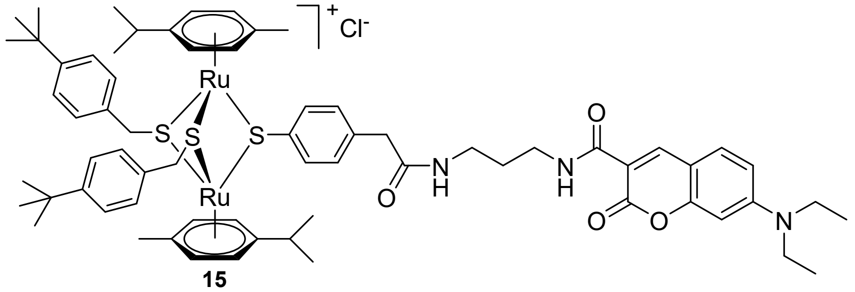

15

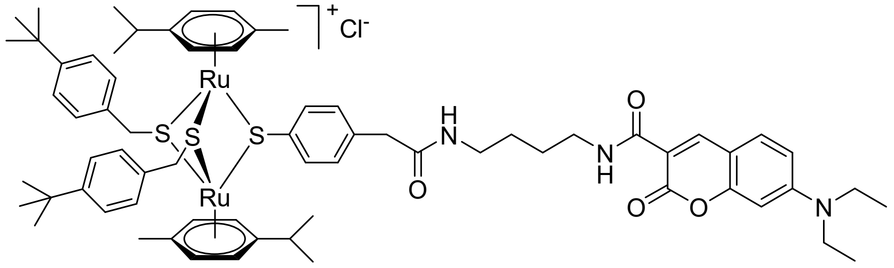

16a

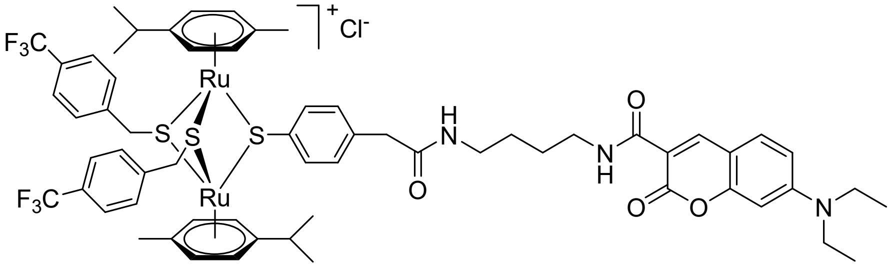

16b

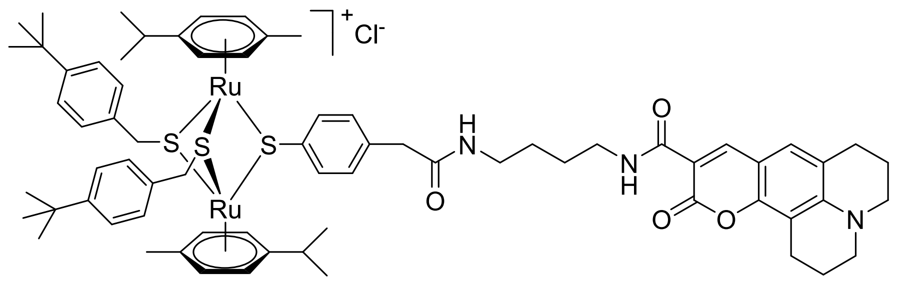

17a

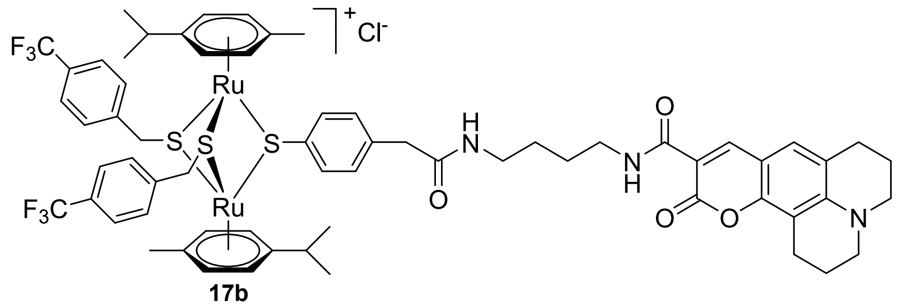

17b

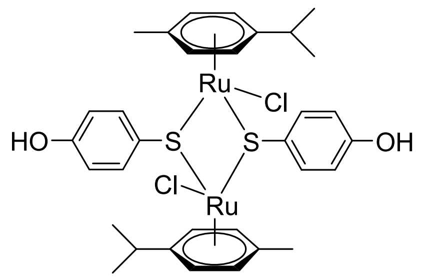

18

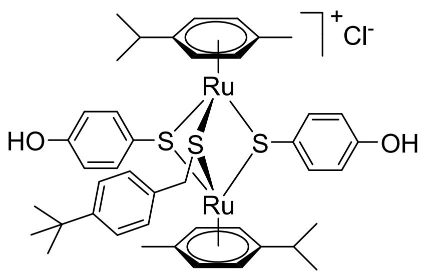

19

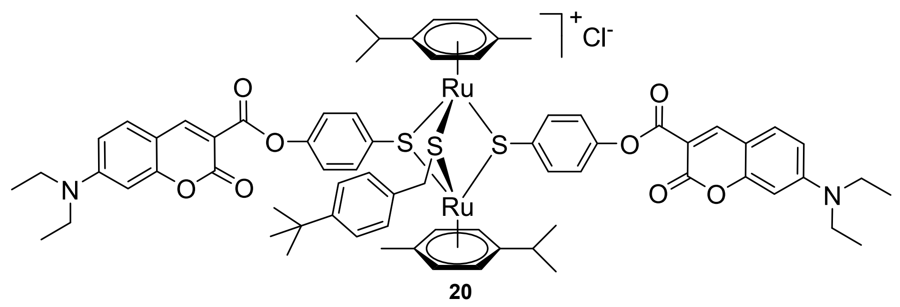

20

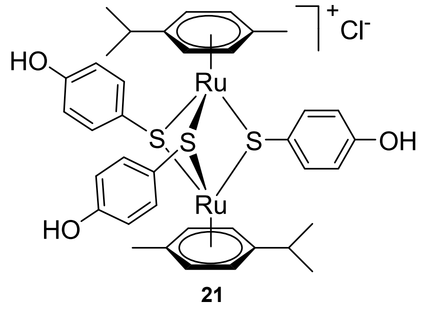

21

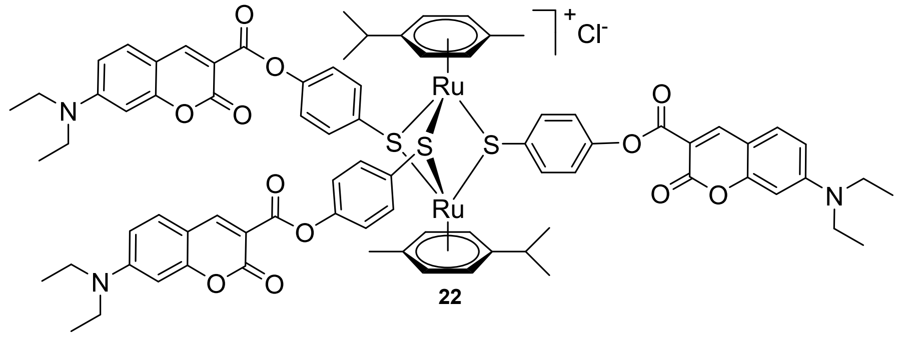

22

References

[1] M. Fischer, J. Georges, *Chem. Phys. Lett.* **1996**, *260*, 115-118.

[2] A. P. Basto, J. Muller, R. Rubbiani, D. Stibal, F. Giannini, G. Suss-Fink, V. Balmer, A. Hemphill, G. Gasser, J. Furrer, *Antimicrobial Agents and Chemotherapy* **2017**, *61*.

[3] F. Cherioux, C. M. Thomas, T. Monnier, G. Suss-Fink, *Polyhedron* **2003**, *22*, 543-548.

[4] M. A. Bennett, A. K. Smith, *J. Chem. Soc. Dalton Trans.* **1974**, 233-241.

[5] G. R. Fulmer, A. J. M. Miller, N. H. Sherden, H. E. Gottlieb, A. Nudelman, B. M. Stoltz, J. E. Bercaw, K. I. Goldberg, *Organometallics* **2010**, *29*, 2176-2179.

[6] A. F. Ibao, M. Gras, B. Therrien, G. Suss-Fink, O. Zava, P. J. Dyson, *Eur. J. Inorg. Chem.* **2012**, 1531-1535.

[7] F. Giannini, J. Furrer, G. Suss-Fink, C. M. Clavel, P. J. Dyson, *J. Organomet. Chem.* **2013**, *744*, 41-48.

[8] A. Cilibrizzi, M. Fedorova, J. Collins, R. Leatherbarrow, R. Woscholski, R. Vilar, *Dalton Trans.* **2017**, *46*, 6994-7004; N. Srinivasan, A. Yurek-George, A. Ganesan, *Mol. Diversity* **2005**, *9*, 291-293.
